# Disruption of Crystal Packing in Thieno[2,3-*b*]pyridines Improves Anti-Proliferative Activity

**DOI:** 10.3390/molecules27030836

**Published:** 2022-01-27

**Authors:** Natalie A. Haverkate, Euphemia Leung, Lisa I. Pilkington, David Barker

**Affiliations:** 1 School of Chemical Sciences, University of Auckland, Auckland 1010, New Zealand; natalie.haverkate@auckland.ac.nz (N.A.H.); lisa.pilkington@auckland.ac.nz (L.I.P.); 2 Auckland Cancer Society Research Centre, University of Auckland, Auckland 1023, New Zealand; e.leung@auckland.ac.nz; 3 Department of Molecular Medicine and Pathology, University of Auckland, Auckland 1023, New Zealand; 4 The MacDiarmid Institute for Advanced Materials and Nanotechnology, Victoria University of Wellington, Wellington 6012, New Zealand

**Keywords:** thieno[2,3-*b*]pyridines, esters, anti-proliferative, prodrugs

## Abstract

3-Amino-2-arylcarboxamido-thieno[2,3-*b*]pyridines have been shown to have anti-proliferative activity, but are also known to have poor solubility. This has been previously proposed to be due to their extensive planarity, which allows for intermolecular stacking and crystal packing. We herein report the synthesis of fifteen novel thieno[2,3-*b*]pyridines that have incorporated bulky, but easily cleavable, ester and carbonate functional groups in an effort to decrease crystal packing. The addition of these ‘prodrug-like’ moieties into the thieno[2,3-*b*]pyridine resulted in compounds with increased activity against HCT-116 colon cancer cells and the triple-negative breast cancer cell line MDA-MB-231.

## 1. Introduction

**Figure 1:**
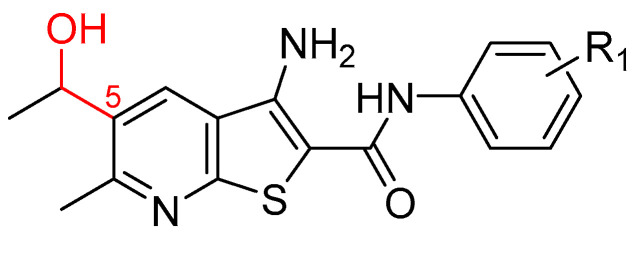
Known anti-proliferative 3-amino-5-(1-hydroxyethyl)-6-methyl-*N*-phenylthieno[2,3-*b*]pyridine-2-carboxamides.

The potent anti-proliferative activity of alcohol-containing thieno[2,3-*b*]pyridines, such as those shown in Figure 1, has been reported [1]. The parent thieno[2,3-*b*]pyridines were initially developed as PI-PLC inhibitors following their discovery through vHTS in 2009 [2], after which structure refinement led to the synthesis of various analogue series that have demonstrated excellent anti-proliferative activity against MDA-MB-231 and HCT-116 cancer cells, with the best compounds exhibiting IC_50_ values in the nM range [1,3].

The potent cytotoxic effect of this class of compounds is likely due to inhibition of the phosphoinositide phospholipase C (PI-PLC) enzyme, which has been found to be upregulated in many cancers [4]. The morphology of cancer cells treated with thienopyridines has been found to be altered, resulting in growth restriction, rounding and blebbing of the plasma membrane [5]. This was further confirmed by studies in which MDA-MB-231 cells that had PLC-δ_1_ and δ_3_ isoform knockdown also showed distorted cell morphology, similar to that described above [5]. Although it has been shown that PI-PLC is a valid target by the aforementioned studies, it must be considered that the thienopyridines could potentially exert some of their anti-proliferative activity through off-target interactions [5], with similar thienopyridines having been implicated with other enzymes, including tyrosyl-DNA phosphodiesterase I (TDP1), A_2A_ receptor antagonists (A_2A_AR), G-protein coupled receptors (GPCRs), copper-trafficking protein Atox, P2Y_12_ receptors, and tubulin [6,7,8,9,10,11,12].

While the anti-proliferative activity of the thieno[2,3-*b*]pyridines has been extensively studied, the potential incorporation of ‘prodrug-like’ moieties into the thieno[2,3-*b*]pyridines has not been explored, despite the proven effectiveness of prodrugs in various chemotherapy drugs [13,14]. The purpose of prodrugs is generally to optimize the absorption, distribution, metabolism, and excretion (ADME) of the parent compound, and a variety of different chemical moieties can be attached to the potential drug structure in order to improve these properties. As the thienopyridines have historically had poor solubility [2,15], it was thought that modification via addition of such a chemical moiety could aid absorption and cell penetration, after which the protecting group could be cleaved by intracellular esterases to allow the thienopyridines to exert their potent cytotoxic effect. In order to improve the pharmacokinetic profile of this class of anti-proliferatives, we sought to synthesise a range of related prodrugs and assess if addition of these moieties affected their activity.

It has been previously shown that thieno[2,3-*b*]pyridines that contain a methylene-hydroxyl group in the C-5 position (Figure 1) in both cyclic and acyclic derivatives have improved anti-proliferative activity versus their equivalent non-hydroxylated analogues, with 2,3-disubstitution (*ortho,meta*) on the phenyl carboxamide leading to excellent cell growth inhibition [3,9,16]. Therefore, investigation of derivatives of the alcohol-containing thieno[2,3-*b*]pyridines was of particular interest for further development.

It was hypothesised that the addition of a range of esters and carbonates could be added to the parent alcohol-containing thienopyridines. This initially seemed to be an unconventional method, as esters are generally added to make a compound more lipophilic and could reduce solubility, and this was supported by logP and logS values (the complete table is shown in the Supplementary Materials), which were calculated to be higher for the esters and carbonates when compared to their parent alcohols. However, in this case, adding groups to the alcohol was hypothesised to disrupt the tightly-packed crystal structure existing in these mainly planar compounds, by increasing the rotatable bonds and adding bulk to the structure. It was postulated that these thieno[2,3-*b*]pyridines were not lacking in aqueous solubility due to a lack of polar groups, but because of the high level of strong intermolecular interactions between the molecules. Thienopyridines are highly planar molecules and thought to pack tightly due to these intermolecular attractive forces, including hydrogen bonds and π-stacking interactions [15]. This is exhibited in the high melting points of the thienopyridines, with elevated melting points reflecting high crystal packing energy, which is known to be correlated with poor solubility [17,18]. Additionally, it was considered that introduction of ester/carbonate groups could assist in the cellular penetration of the molecules.

**Figure 2:**
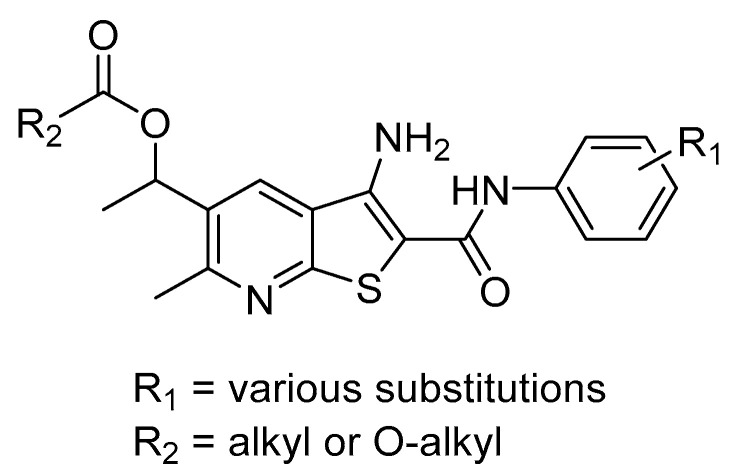
General structure of thieno[2,3-*b*]pyridine derivatives targeted in this work.

The suggested modifications of ester and carbonate moieties on the 5-CHOH group (Figure 2) are groups that have been utilised in many comparable FDA approved drugs. Examples of these include abiraterone acetate (Zytiga), which is used to treat metastatic castration-resistance prostate cancer (CYP17A1 inhibitor), and uridine triacetate (Xuriden), which is used to treat hereditary orotic aciduria (pyrimidine analogue for uridine replacement). These drugs both contain alcohol groups which have been converted to esters to facilitate absorption [17].

As these types of modifications were proven to be useful both in clinical practice and in the literature, it was decided to create and study a series of ‘prodrug-like’ thieno[2,3-*b*]pyridine analogues. Herein, we report the synthesis and anti-proliferative activity of fifteen novel thieno[2,3-*b*]pyridines that fulfil the modifications proposed in Figure 2.

## 2. Results and Discussion

### 2.1. Synthesis of Targeted Compounds

**Scheme 1:**
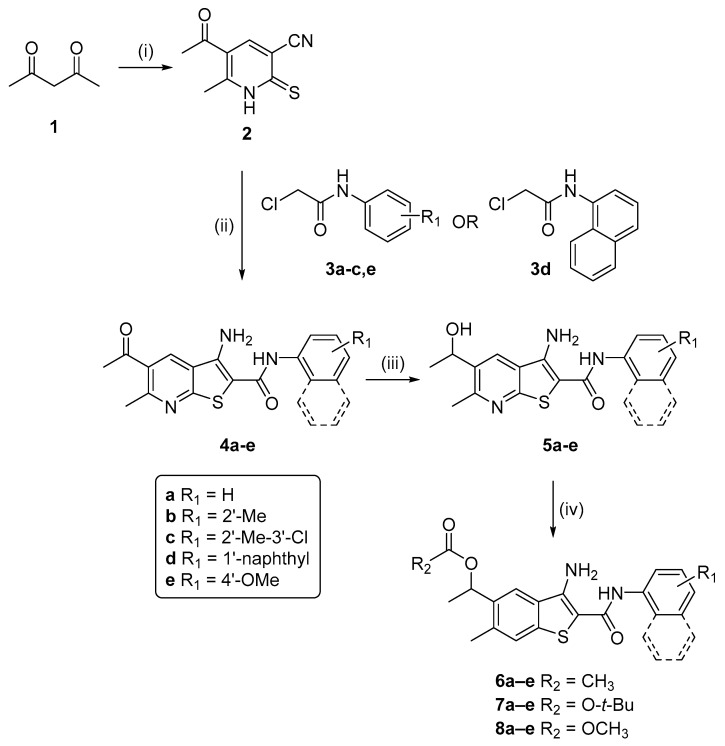
Synthesis of 1-(3-amino-6-methyl-2-(phenylcarbamoyl)thieno[2,3-*b*]pyridin-5-yl)ethyl acetates and carbonates **6a**–**e**, **7a**–**e** and **8a**–**e**. Reagents and conditions (i) acetylacetone **1** (1.0 equiv.), DMF.DMA (1.0 equiv.), dioxane, r.t., 24 h; Na (2.0 equiv.), cyanothioacetamide (1.0 equiv.), MeOH, r.t., 1 h; reflux, 4 h; 2 M HCl, **2** 97%; (ii) carbonitrile **2** (1.0 equiv.), chloroarylacetamides **3a**–**e** (1.0 equiv.), Na_2_CO_3_ (2.0 equiv.), abs. EtOH, reflux, 48 h, **4a**–**e** 47%-quant.; (iii) ketones **4a**–**e** (1.0 equiv.), NaBH_4_ (1.0 equiv.), MeOH, THF, r.t., 2 h; H_2_O, **5a**–**e** 50%-quant.; (iv) alcohols **5a**–**e** (1.0 equiv.), acetic anhydride, Boc anhydride (2.0 equiv.), or methyl chloroformate (1.0 equiv.), DMAP (cat.), pyridine, 10–20 min, r.t., **6a**–**e**, **7a**–**e** and **8a**–**e** 17–68%.

To prepare the required thienopyridine derivatives, previously employed synthetic methods were utilised [19]. The synthesis of carbonitrile **2**, chloroarylacetamides **3a**–**e**, thieno[2,3-*b*]pyridines **4a**–**e** and compounds **5a**–**e** were performed using previously published protocols (Scheme 1), and all detailed information regarding the synthesis of **5a**–**e** is present in the Supplementary Materials.

The aryl carboxamide substitution patterns R_1_ = H, 2′-Me, 2′-Me-3′-Cl, 1′-naphthyl, and 4′-OMe (hereafter collectively referred to as the ‘representative five’) were chosen as they typically give a range of activities. Analogues with 2′-Me-3′-Cl and 1′-naphthyl substitution patterns typically give the best anti-proliferative activity, 4′-OMe the worst, whilst H and 2′-Me have moderate activity. It was decided to use this consistent substitution pattern across the studies undertaken, because if new compounds exhibited atypical relative activities that did not match our previous results, this could indicate the compounds were interacting with off-target proteins.

Alcohols **5a**–**e** were then reacted with either acetic anhydride, di-*tert*-butyl dicarbonate, or methyl chloroformate in pyridine, using catalytic amounts of DMAP, to give **6a**–**e**, **7a**–**e**, and **8a**–**e**, respectively (Scheme 1). Reaction times were required to be kept very short (10–20 min) so as to avoid pyrimidinone formation [19] or double addition on both the alcohol and the primary amine, which are both known possible side reactions. It should be noted that compounds where the primary amine have been modified to amides, carbamates, or pyrimidinones have been shown to have no anti-proliferative activity, showing that these more robust functional group transformations render the derivatives inactive [19].

It was observed that the transformation of the alcohols to their ester and carbonate counterparts led to the expected decrease in melting points. Melting points ranged from much greater than 230 °C for the parent alcohols and decreased to a range of 187–230 °C for the fifteen new analogues, with only two exceeding 230 °C: the 4′-OMe substituted analogues **6e** and **8e**. This was likely due to the presence of another oxygen atom, which can act as a hydrogen bond acceptor, increasing intermolecular hydrogen bonding and the melting points of these two analogues. This was potentially not observed in **7e** as the increase in hydrogen bonding was thought to be countered by the presence of the bulky *tert*-butyl group, which could significantly disrupt crystal packing. For the remaining analogues, the decreased melting point was indicative of decreased crystal packing, and consequently, improved solubility. NMR experiments were performed with samples dissolved in DMSO-d6 so as to be consistent with previously obtained spectra for the thieno[2,3-*b*]pyridines [1,20].

### 2.2. Anti-Proliferative Activity

After synthesis of the targeted analogues, their anti-proliferative activities were tested against HCT-116 and MDA-MB-231 cancer cell lines. It was found that the majority of the synthesised derivatives **6a**–**e**, **7a**–**e**, and **8a**–**e** had notable anti-proliferative activity against both cancer cell lines (Table 1). The derivatives that had 2′-Me-3′-Cl and 1′-naphthyl substitution patterns on the aryl carboxamide predictably had the best anti-proliferative activity, whereas the derivatives that had 4′-OMe substitution had the poorest activity, which suggested the compounds were affecting the same intracellular targets as previous analogues. In particular, treatment of cells at 1 μM with compounds **6c**, **8c**, and **8d** demonstrated 99.8% growth inhibition and had IC_50_ values of 11, 15, and 24 nM, respectively, in HCT-116 cancer cells. Lower growth inhibition was observed for MDA-MB-231 cancer cells for these same compounds, but at 1 μM dosing, up to 95.4% cell growth inhibition was still observed, and subsequent testing gave IC_50_ values of 24, 21, and 32 nM for compounds **6c**, **8c**, and **8d**, respectively.

It is important to note that the parent alcohols and their derivatives do contain a chiral centre at the carbonyloxy substituent, however, the alcohols have been previously tested racemically, and molecular modelling studies have shown that both the (*R*)- and (*S*)-enantiomers are likely to bind to the PI–PLC active site [1]. The results of this study by Haverkate et al. showed that the most active 2′-Me-3′-Cl and 1′-naphthyl substituted alcohols had IC_50_ values of 72–171 nM, higher than their derivatives [1]. The activity of the novel ‘prodrug-like’ derivatives was therefore improved when compared to the parent alcohols.

For further comparison, the anti-cancer drugs paclitaxel and doxorubicin have published IC_50_ values of 0.3 µM [21] and 3.16 µM [22], respectively, in MDA-MB-231 cells, highlighting the potency of these novel thienopyridines.

### 2.3. Molecular Modelling Study

**Figure 3:**
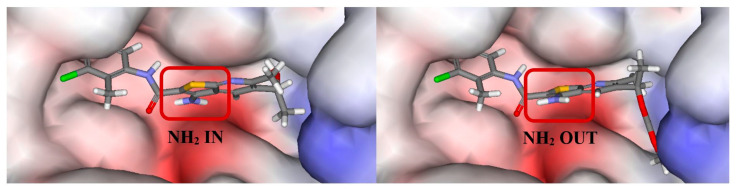
The docked configuration of **5c** (**left**) and **8c** (**right**) in the binding site of PLC-δ_1_ using ChemPLP. Compound **5c** is orientated so that the NH_2_ group points into the binding site and **8c** is orientated so that the NH_2_ group points out of the binding site. Red areas depict regions of partial negative charge, blue areas depict regions of partial positive charge, and grey areas are neutral/lipophilic.

Whilst there is no direct assay to determine PI-PLC activity, molecular modelling has been extensively used to investigate the interaction of compounds with the active site of the enzyme [9,23,24]. Parent alcohols, such as **5c**, are predicted to bind as shown in Figure 3, with important interactions between amino acids GLU341 and HIS311 and the primary amine, as well as the amide carboxyl group. In addition, the alcohol group is predicted to bind with ARG549 inside the active site. Molecular modelling of the derivatives (e.g., carbonate **8c**, Figure 3) predicts that modification of the alcohol significantly reduces the binding. The increased size due to addition of the carbonate does not allow the molecule to fit well within the binding site. This can be seen in Figure 3 with the primary amine group, which is pointed inwards for **5c** (H-bond with GLU341), but upon conversion to **8c**, the NH_2_ group is pointed outwards, eliminating the interaction with GLU3431. This molecule flipping also removes the interaction seen between the alcohol in **5c** with ARG549. This prediction of the flipping in the molecule is due to the increased size and results in a much lower binding efficiency, which would then correlate with a much lower activity [9,23,24,25]. However, the high biological activity of the new derivatives suggests that the added moieties were likely cleaved intracellularly, reforming the parent alcohols, and supported the idea that creating these modified moieties led to an enhanced intracellular concentration of the thieno[2,3-*b*]pyridine, as many (**6c**, **8c**, and **8d**) had better IC_50_ values when compared to their parent alcohols **5c** and **5d**.

### 2.4. Summary

This study considered a new approach for optimizing activity in bioactive thieno[2,3-*b*]pyridines, which was the incorporation of ‘prodrug-like’ moieties on the known anti-proliferative alcohol-containing thienopyridines. It was thought that the addition of ester and carbonate groups would aid solubility by disrupting the planar structure of the thienopyridines, creating a lower energy crystal structure, thereby increasing solubility and improving the intracellular concentrations of the compounds. The success of this was reflected in the lowered melting points of the novel products, and in the improved anti-proliferative activity of the derivative thienopyridines (**6c**, **8c** and **8d**) over their parent alcohols (**5c** and **5d**). Overall, fifteen novel and highly anti-proliferative compounds were successfully synthesised, many of which exhibited record new IC_50_ values. Compounds **6c** and **8c** in particular had IC_50_ values of 11 nM, and 15 nM, respectively, for HCT-116 cells, which are the lowest (and most potent) thienopyridines yet.

## 3. Materials and Methods

### 3.1. Synthesis of the Compounds

General Details: All reactions were carried out under a nitrogen atmosphere in dry, freshly distilled solvents unless otherwise noted. All NMR spectra were recorded on a Bruker Avance DRX 400 MHz spectrometer (Bruker Scientific Instruments, Billerica, MA, USA)at ambient temperatures. Chemical shifts are reported relative to the solvent peak of DMSO (δ 2.50 for ^1^H and δ 39.5 for ^13^C). ^1^H NMR data are reported as position (δ), relative integral, multiplicity (s, singlet; d, doublet; t, triplet; q, quartet; dd, doublet of doublets; td, triplet of doublets; tt, triplet of triplets; m, multiplet; br, broad peak), coupling constant (*J*, Hz), and the assignment of the atom. ^13^C NMR data are reported as position (δ) and assignment of the atom. All NMR assignments were performed using HSQC and HMBC experiments. All melting points for solid compounds, given in degrees Celsius (°C), were measured using a Reicher-Kofler block and are uncorrected. Infrared (IR) spectra were recorded using a Perkin-Elmer Spectrum 1000 series Fourier Transform Infrared ATR spectrometer (Perkin Elmer, Waltham, MA, USA) Absorption maxima are expressed in wavenumbers (cm^−1^). High-resolution mass spectroscopy (HRMS) was carried out by either chemical ionization (CI) or electrospray ionization (ESI) on a MicroTOF-Q mass spectrometer (Bruker Scientific Instruments, Billerica, MA, USA). Unless noted, chemical reagents were used as purchased. General procedures, synthetic experimental methods, and full characterization data (including copies of NMR spectra for all synthesized final compounds) can be found in the Supplementary Materials.

### 3.2. Cell Proliferation Assay

The synthesised ‘prodrug-like’ thieno[2,3-*b*]pyridines were measured for their anti-proliferative activity against triple negative breast cancer MDA-MB-231 and colorectal cancer HCT-116 cell lines (purchased from the American Type Culture Collection, Manassas, VA, USA) using ^3^H-thymidine incorporation assays. As previously described in Leung et al. [6], 3000 cells were seeded in each well using 96-well plates with varying concentrations of thieno[2,3-*b*]pyridines for three days. Experiments were performed in triplicate, with a minimum of two experimental repeats. An amount of 0.04 μCi of ^3^H-thymidine was added to each well five hours prior to harvest, after which the cells were harvested onto glass fibre filters using an automated TomTec harvester (Tomtec, Chicago, IL, USA). The filters were incubated with Betaplate Scint and thymidine incorporation was determined with a Trilux/Betaplate counter (Perkin Elmer, Waltham, MA, USA). Effects of inhibitors on the incorporation of ^3^H-thymidine into DNA were determined relative to the control samples (a previously known active compound and triplicate wells with no inhibitor).

### 3.3. Molecular Modelling

The thieno[2,3-*b*]pyridines synthesised in this study were docked into the mammalian PI-PLC-δ_1_ crystal structure, which was obtained from the Protein Data Bank (PDB ID: 1DJX-04, from Rattus norvegicus). The software GOLD suite version 5.8.1 was used to prepare the crystal structure for docking, by the addition of hydrogen atoms and the removal of water molecules, and the co-crystallised ligand (D-myo-inositol-1,4,5-triphosphate, IP_3_). Basic amino acids were assumed to be protonated, and acidic amino acids deprotonated, in order to closely resemble a cell’s in vivo environment. The coordinates of the binding pocket were located at the Ca^2+^ ion, i.e., x = 126.257, y = 38.394, z = 22.370, as stated in the literature, with a 10 Å radius. ChemDraw 3D 15.0 was used to build the thieno[2,3-*b*]pyridines and to perform energy minimisation (MM2) of all studied structures. For each ligand to be docked, 50 docking runs were allowed at 100% search efficiency. The scoring functions ChemPLP, GoldScore, ChemScore, and ASP were implemented to validate the predicted binding modes and relative energies of the ligands using the GOLD suite version (CSD, Cambridge, UK) 5.8.1.

## Figures and Tables

**Table 1:** Anti-proliferative activity (at 1 µM) and IC_50_ (nM) of Series 1 compounds against HCT-116 and MDA-MB-231 cancer cell lines.

	Mean Relative Growth of 1 μM in Cancer Cell Line (%)		IC_50_ (nM)	
	HCT-116	MDA-MB-231	HCT-116	MDA-MB-231
5a *	95.3	107.4	-	-
5b *	99.9	97.9	-	-
5c *	2.2	11.7	72	76
5d *	1.7	9.9	171	81
5e *	97.5	114.4	-	-
**6a**	0.8	8.0	224	274
**6b**	1.7	11.7	387	448
**6c**	0.2	4.6	11	24
**6d**	3.1	7.1	-	-
**6e**	70.1	77.0	-	-
**7a**	4.0	24.6	667	768
**7b**	7.8	33.0	776	791
**7c**	0.7	5.7	61	90
**7d**	5.4	7.3	473	449
**7e**	94.8	96.4	-	-
**8a**	7.0	15.2	-	-
**8b**	11.1	16.7	-	-
**8c**	0.2	3.1	15	21
**8d**	0.2	3.1	24	32
**8e**	101.1	107.0	-	-

* denotes previously tested parent alcohol-containing thienopyridines [1]. The most active compounds are shown in the highlighted rows

## Data Availability

Data are contained within the article.

## Supplementary Materials

The following supporting information can be downloaded online, Refs. [1,26] are cited in the Supplementary Materials. Figure S1: ^1^H NMR spectrum of **6a** (400 MHz; DMSO-*d*_6_), Figure S2: ^13^C NMR spectrum of **6a** (100 MHz; DMSO-*d*_6_), Figure S3: ^1^H NMR spectrum of **6b** (400 MHz; DMSO-*d*_6_), Figure S4: ^13^C NMR spectrum of **6b** (100 MHz; DMSO-*d*_6_), Figure S5: ^1^H NMR spectrum of **6c** (400 MHz; DMSO-*d*_6_), Figure S6: ^13^C NMR spectrum of **6c** (100 MHz; DMSO-*d*_6_), Figure S7: ^1^H NMR spectrum of **6d** (400 MHz; DMSO-*d*_6_), Figure S8: ^13^C NMR spectrum of **6d** (100 MHz; DMSO-*d*_6_), Figure S9: ^1^H NMR spectrum of **6e** (400 MHz; DMSO-*d*_6_), Figure S10: ^13^C NMR spectrum of **6e** (100 MHz; DMSO-*d*_6_), Figure S11: ^1^H NMR spectrum of **7a** (400 MHz; DMSO-*d*_6_), Figure S12: C NMR spectrum of **7a** (100 MHz; DMSO-*d*_6_), Figure S13: ^1^H NMR spectrum of **7b** (400 MHz; DMSO-*d*_6_), Figure S14: ^13^C NMR spectrum of **7b** (100 MHz; DMSO-*d*_6_), Figure S15: ^1^H NMR spectrum of **7c** (400 MHz; DMSO-*d*_6_), Figure S16: ^13^C NMR spectrum of **7c** (100 MHz; DMSO-*d*_6_), Figure S17: ^1^H NMR spectrum of **7d** (400 MHz; DMSO-*d*_6_), Figure S18: ^13^C NMR spectrum of **7d** (100 MHz; DMSO-*d*_6_), Figure S19: ^1^H NMR spectrum of **7e** (400 MHz; DMSO-*d*_6_), Figure S20: ^13^C NMR spectrum of **7e** (100 MHz; DMSO-*d*_6_), Figure S21: ^1^H NMR spectrum of **8a** (400 MHz; DMSO-*d*_6_), Figure S22: ^13^C NMR spectrum of **8a** (100 MHz; DMSO-*d*_6_), Figure S23: ^1^H NMR spectrum of **8b** (400 MHz; DMSO-*d*_6_), Figure S24: ^13^C NMR spectrum of **8b** (100 MHz; DMSO-*d*_6_), Figure S25: ^1^H NMR spectrum of **8c** (400 MHz; DMSO-*d*_6_), Figure S26: ^13^C NMR spectrum of **8c** (100 MHz; DMSO-*d*_6_), Figure S27: ^1^H NMR spectrum of **8d** (400 MHz; DMSO-*d*_6_), Figure S28: ^13^C NMR spectrum of **8d** (100 MHz; DMSO-*d*_6_), Figure S29: ^1^H NMR spectrum of **8e** (400 MHz; DMSO-*d*_6_), Figure S30: ^13^C NMR spectrum of **8e** (100 MHz; DMSO-*d*_6_), Figure S31: HRMS of **6a** (top) and **6b** (bottom), Figure S32: HRMS of **6c** (top) and **6d** (bottom), Figure S33: HRMS of **6e** (top) and **7a** (bottom), Figure S34: HRMS of **7b** (top) and **7c** (bottom), Figure S35: HRMS of **7d** (top) and **7e** (bottom), Figure S36: HRMS of **8a** (top) and **8b** (bottom), Figure S37: HRMS of **8c** (top) and **8d** (bottom), Figure S38: HRMS of **8e**, Figure S39: Antiproliferative results of compounds 5**a**–**e**, 6**a**–**e**, 7**a**–**e**, and 8**a**–**e**, Table S1: Computational logP and logS values for thieno[2,3-b]pyridines 5**a**–**e**, 6**a**–**e**, 7**a**–**e**, and 8**a**–**e** as calculated by ChemDraw Professional 19.1.1.21, Table S2: Melting points of compounds 5**a**–**e**, 6**a**–**e**, 7**a**–**e**, and 8**a**–**e**.

## Sample Availability

Samples of the compounds are available from the authors upon request.
