# Supplementary material for: Disruption of Crystal Packing in Thieno[2,3-b]pyridines Improves Anti-Proliferative Activity
Source: Molecules. 2022 Jan 27;27(3):836. doi: 10.3390/molecules27030836 (PMC8840025; doi:10.3390/molecules27030836)
Supplement: Supplementary file 1 [file molecules-27-00836-s001.zip › molecules-1520765-supplementary.pdf]

Supplementary Materials for:

Disruption of Crystal Packing in Thieno[2,3-*b*]pyridines Improves Anti-proliferative Activity

Natalie A. Haverkate,[1] Euphemia Leung[2], Lisa I. Pilkington[1], David Barker[1,3]*

[1]School of Chemical Sciences, University of Auckland, Auckland 1010, New Zealand

[2]Auckland Cancer Society Research Centre and Department of Molecular Medicine and Pathology, University of Auckland, Grafton, Auckland 1023, New Zealand

[3]The MacDiarmid Institute for Advanced Materials and Nanotechnology, Victoria University of Wellington, Wellington 6012, New Zealand

Corresponding author. d.barker@auckland.ac.nz ; Tel.: +64-9-373-7599

Table of Contents:

General synthetic procedures S2
Synthetic procedures and compound characterisation data S3-S19
^{1}H and ^{13}C NMR spectra for compounds S20-S49
HRMS data for compounds S50-S57
Computational logP and logS values S58
Melting points S59
Anti-proliferative data for compounds S60
References S61

*General Procedure A: Synthesis of thieno[2,3-*b*]pyridine-2-carboxamide derivatives* ***4a-e***

A mixture of carbonitrile **2** (1 equiv.), acetamides **3a-e** (1 equiv.), and anhydrous sodium carbonate (1.06-2 equiv.) in absolute ethanol (4.00 mL per mmol acetamide) was stirred at reflux for 24-48 h. The ethanol was then removed *in vacuo* and the remaining residue

recrystallised from methanol to give the thieno[2,3-*b*]pyridine-2-carboxamide derivatives **4a-e**.

*General procedure B: Synthesis of thieno[2,3-*b*]pyridine-2-carboxamide alcohols **5a-e***

To a solution of ketones **4a-e** (1 equiv.) in dry THF was added a solution of sodium borohydride (1 equiv.) in methanol, dropwise, over 15 min. The mixture was stirred at r.t. for 2 h, water added and stirred for a further 5 min before the volatiles were removed *in vacuo.* The residue was diluted with water, extracted with ethyl acetate, washed with H_2O and dried with $MgSO_4$ to give the thieno[2,3-*b*]pyridine-2-carboxamide alcohols **5a-e**.

*General Procedure C: Synthesis of esters and carbonates **6a-e**, 7**a-e** and **8a-e***

To a stirred solution of alcohol (1 equiv.) and DMAP (2.5 equiv.) in dry pyridine under an atmosphere of N_2 at r.t. was added Boc or acetic anhydride, or methyl chloroformate (2.0 equiv.) dropwise. The mixture was monitored by TLC over 20 min, after which the mixture was quenched with equivalent portions of sat. aq. $NaHCO_3$ followed by 1 M HCl. The mixture was extracted with ethyl acetate, washed with water, dried with $MgSO_4$, and the solvent removed *in vacuo* to give the crude product, which was purified using flash chromatography to give the desired product.

5-Acetyl-6-methyl-2-thioxo-1,2-dihydropyridine-3-carbonitrile 2

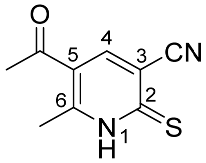

A mixture of acetylacetone **1** (1.00 mL, 10.0 mmol) and *N,N*-dimethylformamide dimethyl acetal (1.33 mL, 10.0 mmol) in dry dioxane (10.0 mL) was stirred under an atmosphere of nitrogen at r.t. overnight. A second mixture of sodium methoxide was prepared by adding sodium (0.46 g, 20.0 mmol) to dry methanol (10.5 mL, 0.260 mol). Cyanothioacetamide (1.00 g, 10.0 mmol) was added to the sodium methoxide and stirred for 10 min, after which the thioacetamide mixture was added to the acetone mixture and stirred for 1 h at r.t.. This was followed by heating at reflux for 4 h. After cooling, the mixture was acidified with ice cold 2 M HCl and water (10 mL) added. The resulting solid was collected by filtration to give the *title compound* **2** (1.86 g, 97%) as a light brown solid. m.p. 200-202 °C (Lit. 230-232 °C).[1] δ_H (400

MHz, $(CD_3)_2SO$) 2.51 (3H, s, $COCH_3$), 2.64 (3H, s, 6-CH_3), 8.55 (1H, s, H-4), 14.26 (1H, br s, NH). The ^{1}H NMR values were in agreement with literature values.[26]

5-Acetyl-3-amino-6-methyl-*N*-phenylthieno[2,3-*b*]pyridine-2-carboxamide 4a

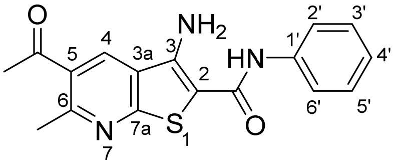

The reaction was carried out following general procedure A using carbonitrile **2** (100 mg, 0.52 mmol), acetamide **3a** (110 mg, 0.52 mmol) and anhydrous sodium carbonate (60.0 mg, 0.55 mmol) in absolute ethanol (4.00 mL) to give the *title compound* **4a** (116 mg, 69%) as a light brown solid. m.p. 228-230 °C (Lit. 228-230 °C).[2] δ_H (400 MHz, $(CD_3)_2SO$) 2.66 (3H, s, 5-$COCH_3$), 2.75 (3H, s, 6-CH_3), 7.07 (1H, t, J = 7.8 Hz, H-4'), 7.32 (2H, t, J = 7.8 Hz, H-3' and H-5'), 7.50 (2H, br s, NH_2), 7.68 (2H, d, J = 7.8 Hz, H-2' and H-6'), 9.05 (1H, s, H-4), 9.40 (1H, br s, NH). The ^{1}H NMR values were in agreement with literature values.[1]

5-Acetyl-3-amino-6-methyl-*N*-(*o*-tolyl)thieno[2,3-*b*]pyridine-2-carboxamide 4b

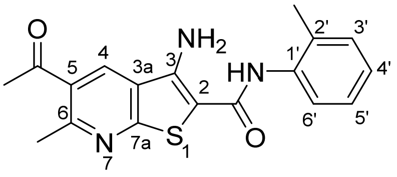

The reaction was carried out following general procedure A using carbonitrile **2** (100 mg, 0.52 mmol), acetamide **3b** (100 mg, 0.52 mmol) and anhydrous sodium carbonate (60.0 mg, 0.55 mmol) in absolute ethanol (4.00 mL) to give the *title compound* **4b** (112 mg, 63%) as a yellow solid. m.p. > 230 °C (Lit. > 230 °C).[2] δ_H (400 MHz, $(CD_3)_2SO$) 2.23 (3H, s, 2'-CH_3), 2.66 (3H, s, 5-$COCH_3$), 2.75 (3H, s, 6-CH_3), 7.13 (1H, t, J = 7.3 Hz, H-4'), 7.19 (1H, t, J = 7.3 Hz, H-5'), 7.26 (1H, d, J = 7.3 Hz, H-3'), 7.33 (1H, d, J = 7.3 Hz, H-6'), 7.35 (2H, br s, NH_2), 9.02 (1H, s, H-4), 9.18 (1H, br s, NH). The ^{1}H NMR values were in agreement with literature values.[1]

5-Acetyl-3-amino-*N*-(3'-chloro-2'-methylphenyl)-6-methylthieno[2,3-*b*]pyridine-2-carboxamide 4c

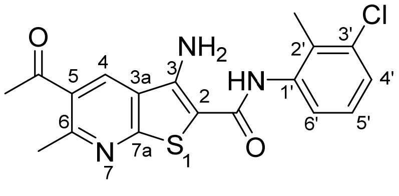

The reaction was carried out following general procedure A using carbonitrile **2** (100 mg, 0.52 mmol), acetamide **3c** (110 mg, 0.52 mmol) and anhydrous sodium carbonate (110 mg, 1.04 mmol) in absolute ethanol (4.00 mL) to give the *title compound* **4c** (207 mg, quant.) as a yellow/cream solid. m.p. > 230 °C (Lit. > 230 °C).[2] δ_H (400 MHz, $(CD_3)_2SO$) 2.24 (3H, s, 2'-CH_3), 2.66 (3H, s, 5-$COCH_3$), 2.75 (3H, s, 6-CH_3), 7.21 (1H, t, *J* = 8.0 Hz, H-5'), 7.31 (2H, d, *J* = 8.0 Hz, H-4' and H-6'), 7.40 (2H, br s, NH_2), 9.03 (1H, s, H-4), 9.41 (1H, br s, NH). The ^{1}H NMR values were in agreement with literature values.[1]

5-Acetyl-3-amino-6-methyl-*N*-(naphthalen-1'-yl)thieno[2,3-*b*]pyridine-2-carboxamide 4d

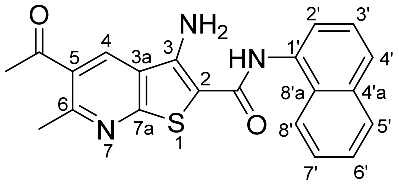

The reaction was carried out following general procedure A using carbonitrile **2** (100 mg, 0.52 mmol), acetamide **3d** (110 mg, 0.52 mmol) and anhydrous sodium carbonate (110 mg, 1.04 mmol) in absolute ethanol (4.00 mL) to give the *title compound* **4d** (93.0 mg, 47%) as a light yellow solid. m.p. >230 °C (Lit. > 230 °C).[2] δ_H (400 MHz, $(CD_3)_2SO$) 2.67 (3H, s, 5-$COCH_3$), 2.76 (3H, s, 6-CH_3), 7.40 (2H, br s, NH_2), 7.46-7.51 (3H, m, 3 × Ar-CH), 7.66-7.71 (2H, m, 2 × Ar-CH), 7.90-7.91 (1H, m, Ar-CH), 8.07 (1H, br, s, Ar-CH), 8.99 (1H, s, H-4), 9.66 (1H, br s, NH). The ^{1}H NMR values were in agreement with literature values.[1]

5-Acetyl-3-amino-*N*-(4'-methoxyphenyl)-6-methylthieno[2,3-*b*]pyridine-2-carboxamide 4e

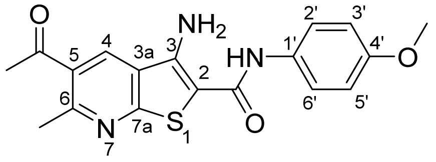

The reaction was carried out following general procedure A using carbonitrile **2** (100 mg, 0.52 mmol), acetamide **3e** (100 mg, 0.52 mmol) and anhydrous sodium carbonate (110 mg, 1.04 mmol) in absolute ethanol (4.00 mL) to give the *title compound* **4e** (159 mg, 88%) as a yellow solid. m.p. 218-220 °C (Lit. 219-221°C).[2] δ_H (400 MHz, $(CD_3)_2SO$) 2.66 (3H, s, 5-$COCH_3$), 2.75 (3H, s, 6-CH_3), 3.74 (3H, s, 4'-OCH_3), 6.89 (2H, d, J = 9.0 Hz, H-3' and H-5'), 7.44 (2H, br s, NH_2), 7.57 (2H, d, J = 9.0 Hz, H-2' and H-6'), 9.03 (1H, s, H-4), 9.36 (1H, br s, NH). The ^{1}H NMR values were in agreement with literature values.[1]

3-Amino-5-(1-hydroxyethyl)-6-methyl-*N*-phenylthieno[2,3-*b*]pyridine-2-carboxamide 5a

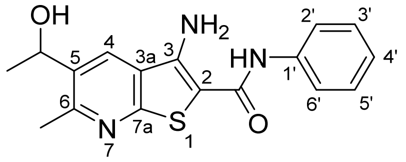

The reaction was carried out following general procedure B using ketone **4a** (0.05 g, 0.15 mmol), $NaBH_4$ (6.00 mg, 0.15 mmol) and methanol (0.51 mL) in THF (7.70 mL) to give the *title compound* **5a** (32.0 mg, 64%) as a yellow solid. m.p. >230 °C (Lit. > 230 °C).[2] δ_H (400 MHz, $(CD_3)_2SO$) 1.40 (3H, d, J = 6.4 Hz, $CHC\underline{H}_3$), 2.61 (3H, s, 6-CH_3), 5.02 (1H, dq, J = 6.4, 3.6 Hz, $C\underline{H}OH$), 5.34 (1H, d, J = 3.6 Hz, $CHO\underline{H}$), 7.06 (1H, t, J = 7.6 Hz, H-4'), 7.31 (2H, d, J = 7.6 Hz, H-3' and H-5'), 7.41 (2H, br s, NH_2), 7.68 (2H, d, J = 7.6 Hz, H-2' and H-6'), 8.54 (1H, s, H-4), 9.34 (1H, br s, NH). The ^{1}H NMR values were in agreement with literature values.[1]

3-Amino-5-(1-hydroxyethyl)-6-methyl-*N*-(*o*-tolyl)thieno[2,3-*b*]pyridine-2-carboxamide 5b

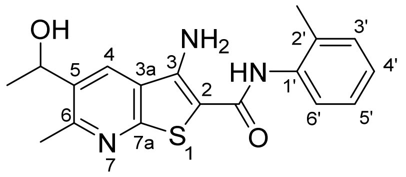

The reaction was carried out following general procedure B using ketone **4b** (0.05 g, 0.15 mmol), $NaBH_4$ (5.60 mg, 0.15 mmol) and methanol (0.50 mL) in THF (7.40 mL) to give the *title compound* **5b** (25.0 mg, 50%) as a yellow solid. m.p. 143-145 °C (Lit. 142-144 °C).[2] δ_H (400 MHz, $(CD_3)_2SO$) 1.40 (3H, d, J = 6.3 Hz, CHC$\underline{H}_3$), 2.22 (3H, s, 2'-CH_3), 2.61 (3H, s, 6-CH_3), 5.02 (1H, dq, J = 6.3, 3.8 Hz, C$\underline{H}$OH), 5.35 (1H, d, J = 3.8 Hz, CHO$\underline{H}$), 7.16 (1H, dd, J = 7.4, 1.8 Hz, H-4'), 7.19 (1H, dd, J = 7.4, 1.8 Hz, H-5'), 7.26-7.32 (4H, m, H-3', H-6' and NH_2), 8.52 (1H, s, H-4), 9.03 (1H, br s, NH). The ^{1}H NMR values were in agreement with literature values.[1]

3-Amino-*N*-(3'-chloro-2'-methylphenyl)-5-(1-hydroxyethyl)-6-methylthieno[2,3-*b*]pyridine-2-carboxamide 5c

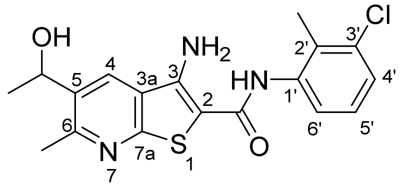

The reaction was carried out following general procedure B using ketone **4c** (0.08 g, 0.21 mmol), $NaBH_4$ (8.10 mg, 0.21 mmol) and methanol (0.71 mL) in THF (10.7 mL) to give the *title compound* **5c** (49.0 mg, 61%) as a yellow solid. m.p. >230 °C (Lit. > 230 °C).[2] δ_H (400 MHz, $(CD_3)_2SO$) 1.40 (3H, d, J = 6.4 Hz, CHC$\underline{H}_3$), 2.23 (3H, s, 2'-CH_3), 2.61 (3H, s, 6-CH_3), 5.02 (1H, dq, J = 6.4, 3.6 Hz, C$\underline{H}$OH), 5.35 (1H, d, J = 3.6 Hz, CHO$\underline{H}$), 7.22 (1H, t, J = 7.8 Hz, H-5'), 7.26 (1H, d, J = 7.8 Hz, H-6'), 7.28-7.35 (3H, m, H-4' and NH_2), 8.53 (1H, s, H-4), 9.30 (1H, br s, NH). The ^{1}H NMR values were in agreement with literature values.[1]

3-Amino-5-(1-hydroxyethyl)-6-methyl-*N*-(naphthalen-1-yl)thieno[2,3-*b*]pyridine-2-carboxamide 5d

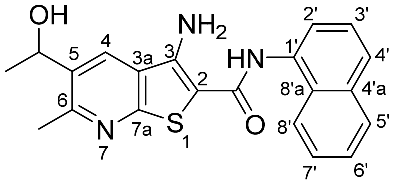

The reaction was carried out following general procedure B using ketone **4d** (0.04 g, 0.107 mmol), $NaBH_4$ (4.00 mg, 0.107 mmol) and methanol (0.35 mL) in THF (5.30 mL) to give the *title compound* **5d** (44.0 mg, quant.) as a yellow solid. m.p. 216-218 °C (Lit. 216-218 °C).[2] δ_H (400 MHz, $(CD_3)_2SO$) 1.40 (3H, d, J = 6.4 Hz, CHC<u>H</u>$_3$), 2.63 (3H, s, 6-CH_3), 5.04 (1H, dq, J = 6.4, 3.7 Hz, C<u>H</u>OH), 5.37 (1H, d, J = 3.7 Hz, CHO<u>H</u>), 7.33 (2H, br s, NH_2), 7.52-7.56 (4H, m, 4 × Ar-CH), 7.84-7.86 (1H, m, Ar-CH), 7.91-7.98 (2H, m, 2 × Ar-CH), 8.54 (1H, s, H-4), 9.62 (1H, br s, NH). The ^{1}H NMR values were in agreement with literature values.[1]

3-Amino-5-(1-hydroxyethyl)-*N*-(4'-methoxyphenyl)-6-methylthieno[2,3-*b*]pyridine-2-carboxamide 5e

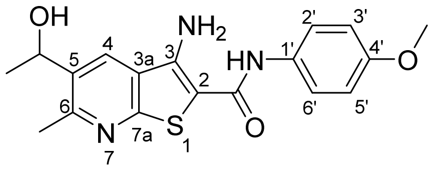

The reaction was carried out following general procedure B using ketone **4e** (0.08 g, 0.23 mmol), $NaBH_4$ (8.50 mg, 0.23 mmol) and methanol (0.74 mL) in THF (11.3 mL) to give the *title compound* **5e** (90.0 mg, quant.) as a yellow solid. m.p. >230 °C (Lit. > 230 °C).[2] δ_H (400 MHz, $(CD_3)_2SO$) 1.40 (3H, d, J = 6.3 Hz, CHC<u>H</u>$_3$), 2.61 (3H, s, 6-CH_3), 3.74 (3H, s, 4'-OCH_3), 5.02 (1H, dq, J = 6.3, 3.4 Hz, C<u>H</u>OH), 5.34 (1H, d, J = 3.4 Hz, CHO<u>H</u>), 6.89 (2H, d, J = 8.8 Hz, H-3' and H-5'), 7.35 (2H, br s, NH_2), 7.56 (2H, d, J = 8.8 Hz, H-2' and H-6'), 8.52 (1H, s, H-4), 9.24 (1H, br s, NH). The ^{1}H NMR values were in agreement with literature values.[1]

1-(3-Amino-6-methyl-2-(phenylcarbamoyl)thieno[2,3-*b*]pyridin-5-yl)ethyl acetate 6a

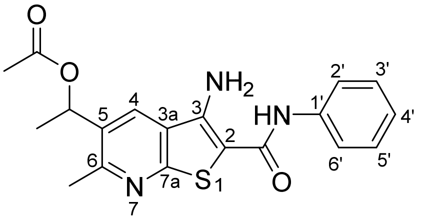

The reaction was carried out following General Procedure C using alcohol **5a** (14.0 mg, 0.04 mmol), DMAP (13.0 mg, 0.11 mmol) and acetic anhydride (0.08 mL, 0.09 mmol) in dry pyridine (0.50 mL) for 15 min then purified with flash chromatography (3:1 petroleum ether, ethyl acetate) to give the *title compound* **6a** (10.0 mg, 63%) as a pale yellow crystalline solid. m.p. 203-205 °C. δ_H (400 MHz, $(CD_3)_2SO$) 1.55 (3H, d, J = 6.5 Hz, 5-CHC$\underline{H}_3$), 2.08 (3H, s, acetyl $COCH_3$), 2.64 (3H, s, 6-CH_3), 6.01 (1H, q, J = 6.5 Hz, 5-C$\underline{H}CH_3$), 7.07 (1H, tt, J = 7.5, 1.1 Hz, H-4'), 7.32 (2H, t, J = 7.5 Hz, H-3' and H-5'), 7.41 (2H, br s, NH_2), 7.69 (2H, dd, J = 7.5, 1.1 Hz, H-2' and H-6'), 8.52 (1H, s, H-4), 9.39 (1H, br s, NH). δ_C (100 MHz, $(CD_3)_2SO$) 20.8 and 20.9 (5-CH$\underline{C}H_3$ and acetyl CO$\underline{C}H_3$), 22.4 (6-CH_3), 68.4 (5-$\underline{C}HCH_3$), 96.0 (C-2), 121.1 (C-2' and C-6'), 123.3 (C-4'), 124.5 (C-3a), 128.1 (C-4), 128.4 (C-3' and C-5'), 132.0 (C-5), 138.0 (C-1'), 147.0 (C-3), 156.8 (C-6), 157.2 (C-7a), 163.9 (2-CONH), 169.8 (acetyl $\underline{C}OCH_3$). ν_{max} (ATR)/cm^{-1} 3437 (N-H amide), 3329 (N-H amine), 2929 (C-H aromatic), 2825 (C-H alkane), 1735 (C=O carbonyl), 1608 (C=O amide), 1529 (C=C aromatic), 1438 (-C-H bending), 1223 (C-N aromatic), 1102 (C-O ether), 1075 (C-N aliphatic). *m/z* (ESI^+): 370 (MH^+, 100%). HRMS (ESI^+) found (MH^+): 370.1214 $C_{19}H_{20}N_3O_3S$ requires 370.1220.

1-(3-Amino-6-methyl-2-(o-tolylcarbamoyl)thieno[2,3-*b*]pyridin-5-yl)ethyl acetate 6b

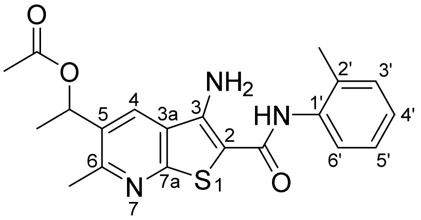

The reaction was carried out following General Procedure C using alcohol **5b** (26 mg, 0.08 mmol), DMAP (23.0 mg, 0.19 mmol) and acetic anhydride (0.01 mL, 0.15 mmol) in dry pyridine (0.60 mL) for 15 min then purified with flash chromatography (3:1 petroleum ether, ethyl acetate) to give the *title compound* **6b** (15.0 mg, 54%) as a pale yellow crystalline solid.

m.p. 196-198 °C. δ_H (400 MHz, $(CD_3)_2SO$) 1.56 (3H, d, *J* = 6.5 Hz, 5-CHC$\underline{H}_3$), 2.08 (3H, s, acetyl $COCH_3$), 2.22 (3H, s, 2'-CH_3), 2.64 (3H, s, 6-CH_3), 6.01 (1H, q, *J* = 6.5 Hz, 5-C$\underline{H}CH_3$), 7.15 (1H, td, *J* = 7.3, 1.3 Hz, H-4'), 7.20 (1H, td, *J* = 7.3, 1.3 Hz, H-5'), 7.25 (1H, dd, *J* = 7.3, 1.3 Hz, H-3'), 7.29 (2H, br s, NH_2), 7.32 (1H, dd, *J* = 7.3, 1.3 Hz, H-6'), 8.50 (1H, s, H-4), 9.08 (1H, br s, NH). δ_C (100 MHz, $(CD_3)_2SO$) 17.9 (2'-CH_3), 20.8 and 20.9 (5-CH$\underline{C}H_3$ and acetyl CO$\underline{C}H_3$), 22.2 (6-CH_3), 68.4 (5-$\underline{C}HCH_3$), 96.5 (C-2), 124.6 (C-3a), 125.8 and 125.9 (C-4' and C-5'), 126.8 (C-6'), 128.0 (C-4), 130.1 (C-3'), 131.9 (C-5), 133.9 (C-2'), 136.4 (C-1'), 146.3 (C-3), 156.6 (C-6), 157.1 (C-7a), 163.9 (2-CONH), 169.8 (acetyl $\underline{C}OCH_3$). ν_{max} (ATR)/cm^{-1} 3406 (N-H amide), 3294 (N-H amine), 2970 (C-H aromatic), 2930 (C-H alkane), 1738 (C=O carbonyl), 1610 (C=O amide), 1514 (C=C aromatic), 1489 (-C-H bending), 1232 (C-N aromatic), 1114 (C-O ether), 1072 (C-N aliphatic). *m/z* (ESI^+): 384 (MH^+, 100%), 346 (97%), 324 (55%). HRMS (ESI^+) found (MH^+): 384.1370 $C_{20}H_{22}N_3O_3S$ requires 384.1376.

1-(3-Amino-2-((3'-chloro-2'-methylphenyl)carbamoyl)-6-methylthieno[2,3-*b*]pyridin-5-yl)ethyl acetate 6c

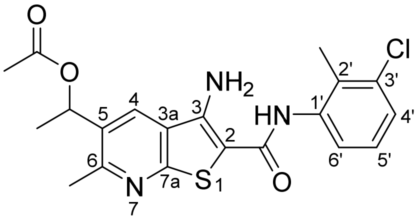

The reaction was carried out following General Procedure C using alcohol **5c** (0.10 g, 0.27 mmol), DMAP (81.0 mg, 0.67 mmol) and acetic anhydride (0.05 mL, 0.53 mmol) in dry pyridine (2 mL) for 15 min then purified with flash chromatography (3:1 petroleum ether, ethyl acetate) to give the *title compound* **6c** (62.0 mg, 56%) as a pale yellow crystalline solid. m.p. 209-211 °C. δ_H (400 MHz, $(CD_3)_2SO$) 1.55 (3H, d, *J* = 6.4 Hz, 5-CHC$\underline{H}_3$), 2.08 (3H, s, acetyl $COCH_3$), 2.23 (3H, s, 2'-CH_3), 2.64 (3H, s, 6-CH_3), 6.01 (1H, q, *J* = 6.4 Hz, 5-C$\underline{H}CH_3$), 7.22 (1H, t, *J* = 7.5 Hz, H-5'), 7.28 (1H, dd, *J* = 7.5, 1.4 Hz, H-6'), 7.34 (1H, dd, *J* = 7.5, 1.4 Hz, H-4'), 7.33 (2H, br s, NH_2), 8.51 (1H, s, H-4), 9.36 (1H, br s, NH). δ_C (100 MHz, $(CD_3)_2SO$) 15.4 (2'-CH_3), 20.8 and 20.9 (5-CH$\underline{C}H_3$ and acetyl CO$\underline{C}H_3$), 22.3 (6-CH_3), 68.4 (5-$\underline{C}HCH_3$), 95.8 (C-2), 124.5 (C-3a), 126.2 (C-6'), 126.5 (C-4'), 126.7 (C-5'), 128.1 (C-4), 132.0 (C-5), 132.5 (C-2'), 133.6 (C-3'), 138.1 (C-1'), 146.8 (C-3), 156.8 (C-6), 157.2 (C-7a), 164.1 (2-CONH), 169.8 (acetyl $\underline{C}OCH_3$). ν_{max} (ATR)/cm^{-1} 3404 (N-H amide), 3351 (N-H amine), 2982 (C-H aromatic), 2936 (C-H alkane), 1740 (C=O carbonyl), 1645 (C=O amide), 1578 (C=C aromatic),

1432 (-C-H bending), 1234 (C-N aromatic), 1183 (C-O ether), 1074 (C-N aliphatic), 768 (C-Cl). *m/z* (ESI$^+$): 442 (^{37}ClMNa$^+$, 40%), 440 (^{35}ClMNa$^+$, 100%). HRMS (ESI$^+$) found (^{37}ClMNa$^+$): 442.0854 $C_{20}H_{20}{}^{37}ClN_3NaO_3S$ requires 442.0781. Found (^{35}ClMNa$^+$): 440.0797 $C_{20}H_{20}{}^{35}ClN_3NaO_3S$ requires 440.0806.

1-(3-Amino-6-methyl-2-(naphthalen-1'-ylcarbamoyl)thieno[2,3-*b*]pyridin-5-yl)ethyl acetate 6d

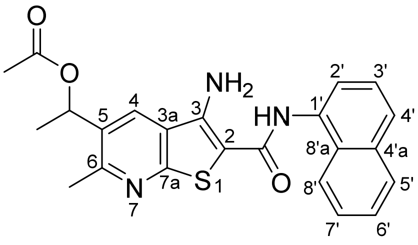

The reaction was carried out following General Procedure C using alcohol **5d** (20.0 mg, 0.05 mmol), DMAP (16.0 mg, 0.13 mmol) and acetic anhydride (0.01 mL, 0.11 mmol) in dry pyridine (0.40 mL) for 15 min then purified with flash chromatography (3:1 petroleum ether, ethyl acetate) to give the *title compound* **6d** (10.0 mg, 45%) as a pale yellow crystalline solid. m.p. 190-192 °C. δ_H (400 MHz, $(CD_3)_2SO$) 1.57 (3H, d, J = 6.5 Hz, 5-CHC<u>H</u>$_3$), 2.09 (3H, s, acetyl $COCH_3$), 2.66 (3H, s, 6-CH_3), 6.02 (1H, q, J = 6.5 Hz, 5-C<u>H</u>CH_3), 7.34 (2H, br s, NH_2), 7.52-7.56 (4H, m, 4 × Ar-CH), 7.83-7.87 (1H, m, Ar-CH), 7.91-7.93 (1H, m, Ar-CH), 7.96-7.98 (1H, m, Ar-CH), 8.52 (1H, s, H-4), 9.67 (1H, br s, NH). δ_C (100 MHz, $(CD_3)_2SO$) 20.8 and 20.9 (5-CH<u>C</u>H_3 and acetyl CO<u>C</u>H_3), 22.3 (6-CH_3), 68.4 (5-<u>C</u>HCH_3), 96.2 (C-2), 123.5 (Ar-CH), 124.3 (Ar-CH), 124.6 (C-3a), 125.5 (Ar-CH), 125.86 (Ar-CH), 125.94 (Ar-CH), 126.2 (Ar-CH), 128.0 (Ar-CH), 128.1 (C-4), 129.7 (Ar-C), 131.9 (C-5), 133.7 (C-1'), 133.9 (Ar-C), 146.7 (C-3), 156.7 (C-6), 157.3 (C-7a), 164.8 (2-CONH), 169.8 (acetyl <u>C</u>OCH_3). ν_{max} (ATR)/cm^{-1} 3394 (N-H amide), 3308 (N-H amine), 2923 (C-H aromatic), 2852 (C-H alkane), 1731 (C=O carbonyl), 1607 (C=O amide), 1516 (C=C aromatic), 1464 (-C-H bending), 1236 (C-N aromatic), 1112 (C-O ether), 1089 (C-N aliphatic). *m/z* (ESI$^+$): 420 (MH$^+$, 100%), 413 (65%), 397 (20%), 382 (90%), 360 (45%). HRMS (ESI$^+$) found (MH$^+$): 420.1367 $C_{23}H_{22}N_3O_3S$ requires 420.1376.

1-(3-Amino-2-((4'-methoxyphenyl)carbamoyl)-6-methylthieno[2,3-*b*]pyridin-5-yl)ethyl acetate 6e

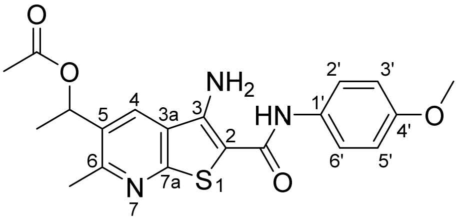

The reaction was carried out following General Procedure C using alcohol **5e** (20.0 mg, 0.06 mmol), DMAP (17.0 mg, 0.14 mmol) and acetic anhydride (0.01 mL, 0.11 mmol) in dry pyridine (1 mL) for 20 min then purified with flash chromatography (3:1 petroleum ether, ethyl acetate) to give the *title compound* **6e** (15.0 mg, 68%) as a pale yellow crystalline solid. m.p. > 230 °C. δ_H (400 MHz, $(CD_3)_2SO$) 1.56 (3H, d, J = 6.5 Hz, 5-CHC$\underline{H}_3$), 2.08 (3H, s, acetyl $COCH_3$), 2.63 (3H, s, 6-CH_3), 3.74 (3H, s, 4'-OCH_3), 6.00 (1H, q, J = 6.5 Hz, 5-C$\underline{H}CH_3$), 6.89 (2H, d, J = 8.7 Hz, H-3' and H-5'), 7.35 (2H, br s, NH_2), 7.57 (2H, d, J = 8.7 Hz, H-2' and H-6'), 8.50 (1H, s, H-4), 9.29 (1H, br s, NH). δ_C (100 MHz, $(CD_3)_2SO$) 20.8 and 20.9 (5-CH$\underline{C}H_3$ and acetyl CO$\underline{C}H_3$), 22.2 (6-CH_3), 55.2 (4'-O$\underline{C}H_3$), 68.4 (5-$\underline{C}HCH_3$), 96.2 (C-2), 113.5 (C-3' and C-5'), 122.9 (C-2' and C-6'), 124.6 (C-3a), 128.0 (C-4), 131.88 and 131.95 (C-1' and C-5), 146.6 (C-3), 155.5 (C-4'), 156.7 (C-6), 157.1 (C-7a), 163.7 (2-CONH), 169.8 (acetyl $\underline{C}OCH_3$). ν_{max} (ATR)/cm^{-1} 3431 (N-H amide), 3327 (N-H amine), 2982 (C-H aromatic), 2891 (C-H alkane), 1737 (C=O carbonyl), 1600 (C=O amide), 1509 (C=C aromatic), 1411 (-C-H bending), 1227 (C-N aromatic), 1168 (C-O ether), 1098 (C-N aliphatic). *m/z* (ESI^+): 422 (MNa^+, 100%). HRMS (ESI^+) found (MNa^+): 422.1135 $C_{20}H_{21}N_3NaO_4S$ requires 422.1145.

1-(3-Amino-6-methyl-2-(phenylcarbamoyl)thieno[2,3-*b*]pyridin-5-yl)ethyl *tert*-butyl carbonate 7a

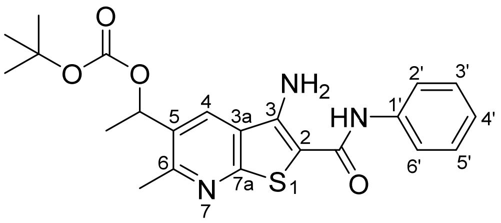

The reaction was carried out following General Procedure C using alcohol **5a** (30.0 mg, 0.09 mmol), DMAP (28.0 mg, 0.23 mmol) and Boc anhydride (40.0 mg, 0.18 mmol) in dry pyridine

(0.70 mL) for 15 min then purified with flash chromatography (3:1 petroleum ether, ethyl acetate) to give the *title compound* **7a** (15.0 mg, 38%) as a pale yellow crystalline solid. m.p. 190-192 °C. δ_H (400 MHz, $(CD_3)_2SO$) 1.39 (9H, s, 3 × *t*-butyl CH_3), 1.56 (3H, d, *J* = 6.5 Hz, 5-CHC$\underline{H}_3$), 2.65 (3H, s, 6-CH_3), 5.88 (1H, q, *J* = 6.5 Hz, 5-C$\underline{H}$CH$_3$), 7.07 (1H, tt, *J* = 7.4, 1.2 Hz, H-4'), 7.32 (2H, t, *J* = 7.4 Hz, H-3' and H-5'), 7.44 (2H, br s, NH_2), 7.69 (2H, d, *J* = 7.4 Hz, H-2' and H-6'), 8.54 (1H, s, H-4), 9.39 (1H, br s, NH). δ_C (100 MHz, $(CD_3)_2SO$) 21.1 (5-CH$\underline{C}$H$_3$), 22.2 (6-CH_3), 27.3 (3 × *t*-butyl CH_3), 71.2 (5-$\underline{C}$HCH$_3$), 81.9 (*t*-butyl quat.), 96.0 (C-2), 121.1 (C-2' and C-6'), 123.3 (C-4'), 124.5 (C-3a), 128.1 (C-4), 128.4 (C-3' and C-5'), 131.9 (C-5), 139.0 (C-1'), 147.0 (C-3), 152.3 (carbonate C=O), 156.7 (C-6), 157.3 (C-7a), 163.9 (2-CONH). ν_{max} (ATR)/cm^{-1} 3409 (N-H amide), 3303 (N-H amine), 2983 (C-H aromatic), 2852 (C-H alkane), 1731 (C=O carbonyl), 1630 (C=O amide), 1591 (C=C aromatic), 1432 (-C-H bending), 1251 (C-N aromatic), 1163 (C-O ether), 1082 (C-N aliphatic). *m/z* (ESI$^+$): 428 (MH$^+$, 100%), 372 (20%), 332 (40%). HRMS (ESI$^+$) found (MH$^+$): 428.1626 $C_{22}H_{26}N_3O_4S$ requires 428.1639.

1-(3-Amino-6-methyl-2-(o-tolylcarbamoyl)thieno[2,3-*b*]pyridin-5-yl)ethyl *tert*-butyl carbonate 7b

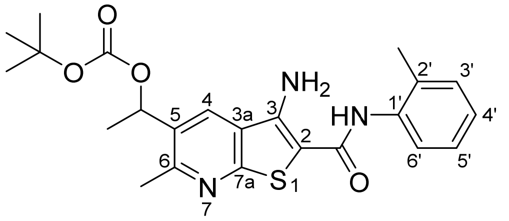

The reaction was carried out following General Procedure C using alcohol **5b** (70.0 mg, 0.21 mmol), DMAP (63.0 mg, 0.51 mmol) and Boc anhydride (89.0 mg, 0.41 mmol) in dry pyridine (1.5 mL) for 30 min then purified with flash chromatography (3:1 petroleum ether, ethyl acetate) to give the *title compound* **7b** (25.0 mg, 27%) as a pale yellow crystalline solid. m.p. 187-189 °C. δ_H (400 MHz, $(CD_3)_2SO$) 1.39 (9H, s, 3 × *t*-butyl CH_3), 1.56 (3H, d, *J* = 6.5 Hz, 5-CHC$\underline{H}_3$), 2.22 (3H, s, 2'-CH_3), 2.65 (3H, s, 6-CH_3), 5.87 (1H, q, *J* = 6.5 Hz, 5-C$\underline{H}$CH$_3$), 7.15 (1H, td, *J* = 7.3, 1.3 Hz, H-4'), 7.19 (1H, td, *J* = 7.3, 1.3 Hz, H-5'), 7.25 (1H, dd, *J* = 7.3, 1.3 Hz, H-3'), 7.31 (1H, dd, *J* = 7.3, 1.3 Hz, H-6'), 7.32 (2H, br s, NH_2), 8.52 (1H, s, H-4), 9.09 (1H, br s, NH). δ_C (100 MHz, $(CD_3)_2SO$) 17.9 (2'-CH_3), 21.1 (5-CH$\underline{C}$H$_3$), 22.2 (6-CH_3), 27.3 (3 × *t*-butyl CH_3), 71.2 (5-$\underline{C}$HCH$_3$), 81.9 (*t*-butyl quat.), 96.4 (C-2), 124.7 (C-3a), 125.8 and 125.9 (C-4' and C-5'), 126.9 (C-6'), 128.0 (C-4), 130.1 (C-3'), 131.8 (C-5), 134.0 (C-2'), 136.4

(C-1'), 146.4 (C-3), 152.3 (carbonate C=O), 156.5 (C-6), 157.3 (C-7a), 163.9 (2-CONH). ν_{max} (ATR)/cm^{-1} 3400 (N-H amide), 3279 (N-H amine), 2978 (C-H aromatic), 2880 (C-H alkane), 1739 (C=O carbonyl), 1609 (C=O amide), 1513 (C=C aromatic), 1283 (C-N aromatic), 1165 (C-O ether), 1089 (C-N aliphatic). *m/z* (ESI$^+$): 442 (MH$^+$, 100%), 386 (20%), 346 (60%). HRMS (ESI$^+$) found (MH$^+$): 442.1787 $C_{23}H_{28}N_3O_4S$ requires 442.1795.

1-(3-Amino-2-((3'-chloro-2'-methylphenyl)carbamoyl)-6-methylthieno[2,3-*b*]pyridin-5-yl)ethyl *tert*-butyl carbonate 7c

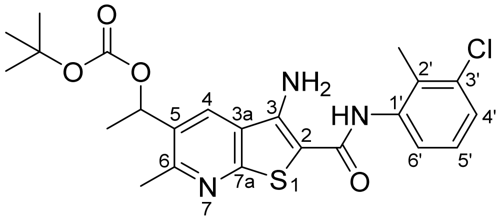

The reaction was carried out following General Procedure C using alcohol **5c** (40.0 mg, 0.11 mmol), DMAP (32.0 mg, 0.27 mmol) and Boc anhydride (46.0 mg, 0.21 mmol) in dry pyridine (1.0 mL) for 15 min then purified with flash chromatography (3:1 petroleum ether, ethyl acetate) to give the *title compound* **7c** (10.0 mg, 20%) as a pale yellow crystalline solid. m.p. 199-201 °C. δ_H (400 MHz, $(CD_3)_2SO$) 1.39 (9H, s, 3 × *t*-butyl CH_3), 1.56 (3H, d, *J* = 6.5 Hz, 5-CHC$\underline{H}_3$), 2.23 (3H, s, 2'-CH_3), 2.65 (3H, s, 6-CH_3), 5.87 (1H, q, *J* = 6.5 Hz, 5-C$\underline{H}$$CH_3$), 7.22 (1H, t, *J* = 7.8 Hz, H-5'), 7.27 (1H, dd, *J* = 7.8, 1.4 Hz, H-6'), 7.34 (1H, dd, *J* = 7.8, 1.4 Hz, H-4'), 7.36 (2H, br s, NH_2), 8.53 (1H, s, H-4), 9.36 (1H, br s, NH). δ_C (100 MHz, $(CD_3)_2SO$) 15.4 (2'-CH_3), 21.1 (5-CH$\underline{C}H_3$), 22.2 (6-CH_3), 27.3 (3 × *t*-butyl CH_3), 71.2 (5-$\underline{C}$HCH_3), 81.9 (*t*-butyl quat.), 95.8 (C-2), 124.6 (C-3a), 126.2 (C-6'), 126.6 (C-4'), 126.7 (C-5'), 128.1 (C-4), 131.9 (C-5), 132.5 (C-2'), 133.6 (C-3'), 138.1 (C-1'), 146.8 (C-3), 152.2 (carbonate C=O), 156.6 (C-6), 157.3 (C-7a), 164.1 (2-CONH). ν_{max} (ATR)/cm^{-1} 3406 (N-H amide), 3290 (N-H amine), 2927 (C-H aromatic), 2845 (C-H alkane), 1742 (C=O carbonyl), 1607 (C=O amide), 1510 (C=C aromatic), 1460 (-C-H bending), 1283 (C-N aromatic), 1168 (C-O ether), 1088 (C-N aliphatic), 779 (C-Cl). *m/z* (ESI$^+$): 478 (^{37}ClMH$^+$, 40%), 476 (^{35}ClMH$^+$, 100%), 413 (43%), 380 (55%). HRMS (ESI$^+$) found (^{37}ClMH$^+$): 478.1369 $C_{23}H_{27}{}^{37}ClN_3O_4S$ requires 478.1383. Found (^{35}ClMH$^+$): 476.1392 $C_{23}H_{27}{}^{35}ClN_3O_4S$ requires 476.1405.

1-(3-Amino-6-methyl-2-(naphthalen-1'-ylcarbamoyl)thieno[2,3-*b*]pyridin-5-yl)ethyl *tert*-butyl carbonate 7d

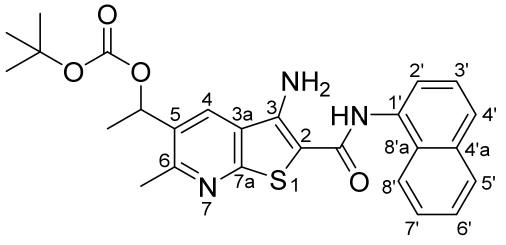

The reaction was carried out following General Procedure C using alcohol **5d** (0.10 g, 0.27 mmol), DMAP (81.0 mg, 0.66 mmol) and Boc anhydride (0.12 g, 0.53 mmol) in dry pyridine (2.0 mL) for 20 min then purified with flash chromatography (3:1 petroleum ether, ethyl acetate) to give the *title compound* **7d** (20.0 mg, 17%) as a pale yellow crystalline solid. m.p. 202-204 °C. δ_H (400 MHz, $(CD_3)_2SO$) 1.40 (9H, s, 3 × *t*-butyl CH_3), 1.57 (3H, d, J = 6.5 Hz, 5-CHC$\underline{H}_3$), 2.67 (3H, s, 6-CH_3), 5.89 (1H, q, J = 6.5 Hz, 5-C$\underline{H}$$CH_3$), 7.36 (2H, br s, NH_2), 7.53-7.56 (4H, m, 4 × Ar-CH), 7.84-7.87 (1H, m, Ar-CH), 7.91-7.93 (1H, m, Ar-CH), 7.95-7.98 (1H, m, Ar-CH), 8.54 (1H, s, H-4), 9.67 (1H, br s, NH). δ_C (100 MHz, $(CD_3)_2SO$) 21.2 (5-CH$\underline{C}H_3$), 22.3 (6-CH_3), 27.3 (3 × *t*-butyl CH_3), 71.2 (5-$\underline{C}$HCH_3), 81.9 (*t*-butyl quat.), 96.2 (C-2), 123.5 (Ar-CH), 124.4 (Ar-CH), 124.6 (C-3a), 125.5 (Ar-CH), 125.9 (Ar-CH), 126.0 (Ar-CH), 126.2 (Ar-CH), 127.99 (Ar-CH), 128.04 (C-4), 129.7 (Ar-C), 131.8 (C-5), 133.7 (C-1'), 133.9 (Ar-C), 146.8 (C-3), 152.3 (carbonate C=O), 156.6 (C-6), 157.4 (C-7a), 164.8 (2-CONH). ν_{max} (ATR)/cm^{-1} 3400 (N-H amide), 3285 (N-H amine), 2978 (C-H aromatic), 2923 (C-H alkane), 1734 (C=O carbonyl), 1605 (C=O amide), 1501 (C=C aromatic), 1262 (C-N aromatic), 1158 (C-O ether), 1081 (C-N aliphatic). *m/z* (ESI^+): 500 (MNa^+, 100%), 478 (50%), 413 (35%). HRMS (ESI^+) found (MNa^+): 500.1606 $C_{26}H_{27}N_3NaO_4S$ requires 500.1614.

1-(3-Amino-2-((4'-methoxyphenyl)carbamoyl)-6-methylthieno[2,3-*b*]pyridin-5-yl)ethyl *tert*-butyl carbonate 7e

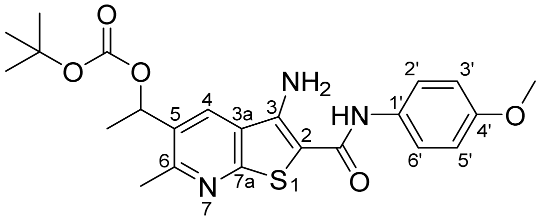

The reaction was carried out following General Procedure C using alcohol **5e** (60.0 mg, 0.17 mmol), DMAP (51.0 mg, 0.42 mmol) and Boc anhydride (73.0 mg, 0.42 mmol) in dry pyridine (1.3 mL) for 15 min then purified with flash chromatography (3:1 petroleum ether, ethyl acetate) to give the *title compound* **7e** (20.0 mg, 26%) as a pale yellow crystalline solid. m.p. 196-198 °C. δ_H (400 MHz, $(CD_3)_2SO$) 1.39 (9H, s, 3 × *t*-butyl CH_3), 1.56 (3H, d, J = 6.5 Hz, 5-CHC$\underline{H}_3$), 2.65 (3H, s, 6-CH_3), 3.74 (3H, s, 4'-OCH_3), 5.87 (1H, q, J = 6.5 Hz, 5-C$\underline{H}CH_3$), 6.89 (2H, d, J = 8.9 Hz, H-3' and H-5'), 7.38 (2H, br s, NH_2), 7.57 (2H, d, J = 8.9 Hz, H-2' and H-6'), 8.52 (1H, s, H-4), 9.29 (1H, br s, NH). δ_C (100 MHz, $(CD_3)_2SO$) 21.1 (5-CH$\underline{C}H_3$), 22.2 (6-CH_3), 27.3 (3 × *t*-butyl CH_3), 55.1 (4'-OCH_3), 71.2 (5-$\underline{C}HCH_3$), 81.9 (*t*-butyl quat.), 96.2 (C-2), 113.5 (C-3' and C-5'), 122.9 (C-2' and C-6'), 124.6 (C-3a), 128.0 (C-4), 131.8 and 131.9 (C-1' and C-5), 146.6 (C-3), 152.3 (carbonate C=O), 155.5 (C-4'), 156.5 (C-6), 157.3 (C-7a), 163.7 (2-CONH). ν_{max} (ATR)/cm^{-1} 3408 (N-H amide), 3292 (N-H amine), 2981 (C-H aromatic), 2925 (C-H alkane), 1745 (C=O carbonyl), 1603 (C=O amide), 1506 (C=C aromatic), 1465 (-C-H bending), 1248 (C-N aromatic), 1166 (C-O ether), 1092 (C-N aliphatic). *m/z* (ESI^+): 480 (MNa^+, 45%), 402 (35%), 362 (100%), 275 (50%). HRMS (ESI^+) found (MNa^+): 480.1561 $C_{23}H_{27}N_3NaO_5S$ requires 480.1564.

1-(3-Amino-6-methyl-2-(phenylcarbamoyl)thieno[2,3-*b*]pyridin-5-yl)ethyl methyl carbonate 8a

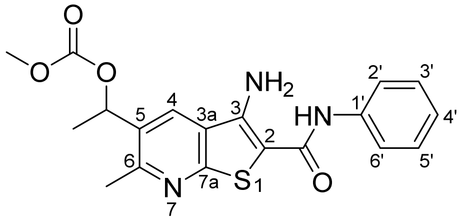

The reaction was carried out following General Procedure C using alcohol **5a** (90.0 mg, 0.28 mmol), DMAP (67.0 mg, 0.55 mmol) and methyl chloroformate (0.02 mL, 0.28 mmol) in dry pyridine (2.0 mL) for 15 min then purified with flash chromatography (3:1 petroleum ether, ethyl acetate) to give the *title compound* **8a** (20.0 mg, 22%) as a pale yellow crystalline solid. m.p. 194-196 °C. δ_H (400 MHz, $(CD_3)_2SO$) 1.60 (3H, d, J = 6.5 Hz, 5-CHC$\underline{H}_3$), 2.67 (3H, s, 6-CH_3), 3.69 (3H, s, OCH_3), 5.94 (1H, q, J = 6.5 Hz, 5-C$\underline{H}$$CH_3$), 7.07 (1H, tt, J = 7.5, 1.2 Hz, H-4'), 7.32 (2H, t, J = 7.5 Hz, H-3' and H-5'), 7.42 (2H, br s, NH_2), 7.69 (2H, d, J = 7.5 Hz, H-2' and H-6'), 8.55 (1H, s, H-4), 9.40 (1H, br s, NH). δ_C (100 MHz, $(CD_3)_2SO$) 21.1 (5-CH$\underline{C}H_3$), 22.3 (6-CH_3), 54.7 (OCH_3), 72.3 (5-$\underline{C}$HCH_3), 96.1 (C-2), 121.1 (C-2' and C-6'), 123.3 (C-4'), 124.5 (C-3a), 128.3 (C-4), 128.4 (C-3' and C-5'), 131.5 (C-5), 139.0 (C-1'), 147.0 (C-3), 154.5 (carbonate C=O), 156.8 (C-6), 157.4 (C-7a), 163.9 (2-CONH). ν_{max} (ATR)/cm^{-1} 3449 (N-H amide), 3327 (N-H amine), 2926 (C-H aromatic), 2852 (C-H alkane), 1741 (C=O carbonyl), 1626 (C=O amide), 1588 (C=C aromatic), 1495 (-C-H bending), 1255 (C-N aromatic), 1100 (C-O ether), 1071 (C-N aliphatic). *m/z* (ESI$^+$): 386 (MH$^+$, 100%), 332 (45%). HRMS (ESI$^+$) found (MH$^+$): 386.1163 $C_{19}H_{20}N_3O_4S$ requires 386.1169.

1-(3-Amino-6-methyl-2-(o-tolylcarbamoyl)thieno[2,3-*b*]pyridin-5-yl)ethyl methyl carbonate 8b

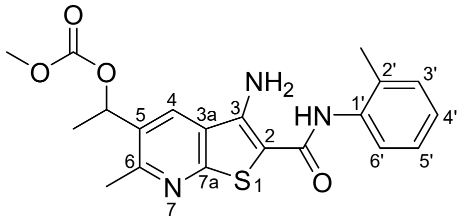

The reaction was carried out following General Procedure C using alcohol **5b** (0.10 g, 0.29 mmol), DMAP (72.0 mg, 0.59 mmol) and methyl chloroformate (0.02 mL, 0.29 mmol) in dry

pyridine (2.0 mL) for 15 min then purified with flash chromatography (3:1 petroleum ether, ethyl acetate) to give the *title compound* **8b** (40.0 mg, 40%) as a pale yellow crystalline solid. m.p. 213-215 °C. δ_H (400 MHz, $(CD_3)_2SO$) 1.60 (3H, d, *J* = 6.5 Hz, 5-CHC$\underline{H}_3$), 2.22 (3H, s, 2'-CH_3), 2.66 (3H, s, 6-CH_3), 3.69 (3H, s, OCH_3), 5.94 (1H, q, *J* = 6.5 Hz, 5-C$\underline{H}CH_3$), 7.15 (1H, td, *J* = 7.3, 1.3 Hz, H-4'), 7.20 (1H, td, *J* = 7.3, 1.3 Hz, H-5'), 7.25 (1H, dd, *J* = 7.3, 1.3 Hz, H-3'), 7.30 (2H, br s, NH_2), 7.31 (1H, dd, *J* = 7.3, 1.3 Hz, H-6'), 8.53 (1H, s, H-4), 9.10 (1H, br s, NH). δ_C (100 MHz, $(CD_3)_2SO$) 17.9 (2'-CH_3), 21.1 (5-CH$\underline{C}H_3$), 22.3 (6-CH_3), 54.7 (OCH_3), 72.4 (5-$\underline{C}HCH_3$), 96.5 (C-2), 124.7 (C-3a), 125.8 and 125.9 (C-4' and C-5'), 126.9 (C-6'), 128.2 (C-4), 130.2 (C-3'), 131.4 (C-5), 133.9 (C-2'), 136.4 (C-1'), 146.4 (C-3), 154.5 (carbonate C=O), 156.6 (C-6), 157.4 (C-7a), 163.9 (2-CONH). ν_{max} (ATR)/cm^{-1} 3400 (N-H amide), 3291 (N-H amine), 2955 (C-H aromatic), 2850 (C-H alkane), 1747 (C=O carbonyl), 1609 (C=O amide), 1596 (C=C aromatic), 1439 (-C-H bending), 1262 (C-N aromatic), 1108 (C-O ether), 1069 (C-N aliphatic). *m/z* (ESI^+): 400 (MH^+, 100%), 346 (73%). HRMS (ESI^+) found (MH^+): 400.1314 $C_{20}H_{22}N_3O_4S$ requires 400.1326

1-(3-Amino-2-((3'-chloro-2'-methylphenyl)carbamoyl)-6-methylthieno[2,3-*b*]pyridin-5-yl)ethyl methyl carbonate 8c

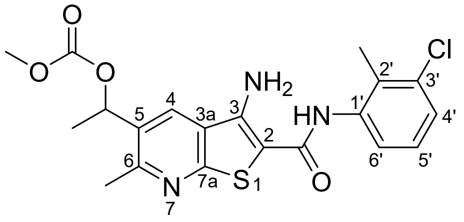

The reaction was carried out following General Procedure C using alcohol **5c** (30.0 mg, 0.08 mmol), DMAP (19.0 mg, 0.18 mmol) and methyl chloroformate (0.01 mL, 0.08 mmol) in dry pyridine (1.0 mL) for 15 min then purified with flash chromatography (3:1 petroleum ether, ethyl acetate) to give the *title compound* **8c** (10.0 mg, 33%) as a pale yellow crystalline solid. m.p. 202-204 °C. δ_H (400 MHz, $(CD_3)_2SO$) 1.60 (3H, d, *J* = 6.5 Hz, 5-CHC$\underline{H}_3$), 2.23 (3H, s, 2'-CH_3), 2.67 (3H, s, 6-CH_3), 3.69 (3H, s, OCH_3), 5.94 (1H, q, *J* = 6.5 Hz, 5-C$\underline{H}CH_3$), 7.23 (1H, t, *J* = 7.8 Hz, H-5'), 7.28 (1H, dd, *J* = 7.8, 1.4 Hz, H-6'), 7.33-7.35 (3H, m, H-4' and NH_2), 8.54 (1H, s, H-4), 9.36 (1H, br s, NH). δ_C (100 MHz, $(CD_3)_2SO$) 15.4 (2'-CH_3), 21.1 (5-CH$\underline{C}H_3$), 22.3 (6-CH_3), 54.7 (OCH_3), 72.3 (5-$\underline{C}HCH_3$), 95.8 (C-2), 124.6 (C-3a), 126.2 (C-6'), 126.6 (C-4'), 126.7 (C-5'), 128.3 (C-4), 131.5 (C-5), 132.5 (C-2'), 133.6 (C-3'), 138.1 (C-1'), 146.8 (C-3), 154.5 (carbonate C=O), 156.7 (C-6), 157.5 (C-7a), 164.1 (2-CONH). ν_{max}

(ATR)/cm^{-1} 3400 (N-H amide), 3291 (N-H amine), 2952 (C-H aromatic), 2851 (C-H alkane), 1747 (C=O carbonyl), 1607 (C=O amide), 1590 (C=C aromatic), 1436 (-C-H bending), 1240 (C-N aromatic), 1106 (C-O ether), 1071 (C-N aliphatic), 774 (C-Cl). *m/z* (ESI$^+$): 436 (^{37}ClMH$^+$, 40%), 434 (^{35}ClMH$^+$, 100%), 380 (45%). HRMS (ESI$^+$) found (^{37}ClMH$^+$): 436.0903 $C_{20}H_{21}{}^{37}ClN_3O_4S$ requires 436.0912. Found (^{35}ClMH$^+$): 434.0930 $C_{20}H_{21}{}^{35}ClN_3O_4S$ requires 434.0936.

1-(3-Amino-6-methyl-2-(naphthalen-1'-ylcarbamoyl)thieno[2,3-*b*]pyridin-5-yl)ethyl methyl carbonate 8d

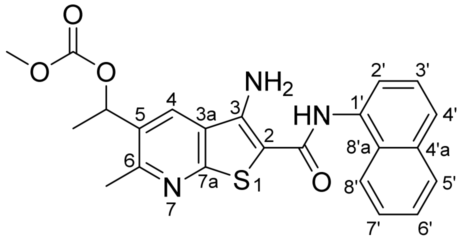

The reaction was carried out following General Procedure C using alcohol **5d** (60.0 mg, 0.16 mmol), DMAP (39.0 mg, 0.32 mmol) and methyl chloroformate (0.01 mL, 0.16 mmol) in dry pyridine (1.0 mL) for 15 min then purified with flash chromatography (3:1 petroleum ether, ethyl acetate) to give the *title compound* **8d** (28.0 mg, 47%) as a pale yellow crystalline solid. m.p. 188-190 °C. δ_H (400 MHz, $(CD_3)_2SO$) 1.61 (3H, d, *J* = 6.5 Hz, 5-CHC$\underline{H}_3$), 2.68 (3H, s, 6-CH_3), 3.70 (3H, s, OCH_3), 5.96 (1H, q, *J* = 6.5 Hz, 5-C$\underline{H}$CH$_3$), 7.35 (2H, br s, NH_2), 7.53-7.56 (4H, m, 4 × Ar-CH), 7.84-7.88 (1H, m, Ar-CH), 7.91-7.94 (1H, m, Ar-CH), 7.96-7.98 (1H, m, Ar-CH), 8.56 (1H, s, H-4), 9.68 (1H, br s, NH). δ_C (100 MHz, $(CD_3)_2SO$) 21.1 (5-CH$\underline{C}$H$_3$), 22.3 (6-CH_3), 54.8 (OCH_3), 72.4 (5-$\underline{C}$HCH$_3$), 96.3 (C-2), 123.4 (Ar-CH), 124.3 (Ar-CH), 124.6 (C-3a), 125.5 (Ar-CH), 125.87 (Ar-CH), 125.95 (Ar-CH), 126.2 (Ar-CH), 128.0 (Ar-CH), 128.3 (C-4), 129.7 (Ar-C), 131.4 (C-5), 133.7 (C-1'), 133.9 (Ar-C), 146.7 (C-3), 154.5 (carbonate C=O), 156.7 (C-6), 157.6 (C-7a), 164.8 (2-CONH). ν_{max} (ATR)/cm^{-1} 3393 (N-H amide), 3308 (N-H amine), 2985 (C-H aromatic), 2851 (C-H alkane), 1748 (C=O carbonyl), 1607 (C=O amide), 1501 (C=C aromatic), 1439 (-C-H bending), 1259 (C-N aromatic), 1106 (C-O ether), 1067 (C-N aliphatic). *m/z* (ESI$^+$): 436 (MH$^+$, 100%), 382 (73%). HRMS (ESI$^+$) found (MH$^+$): 436.1318 $C_{23}H_{22}N_3O_4S$ requires 436.1326.

1-(3-Amino-2-((4'-methoxyphenyl)carbamoyl)-6-methylthieno[2,3-*b*]pyridin-5-yl)ethyl methyl carbonate 8e

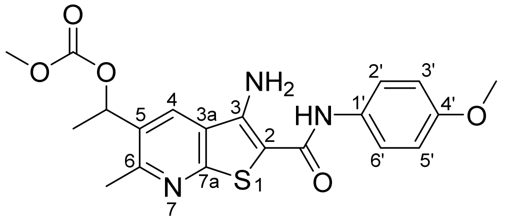

The reaction was carried out following General Procedure C using alcohol **5e** (90.0 mg, 0.25 mmol), DMAP (62.0 mg, 0.50 mmol) and methyl chloroformate (0.02 mL, 0.25 mmol) in dry pyridine (2.0 mL) for 15 min then purified with flash chromatography (3:1 petroleum ether, ethyl acetate) to give the *title compound* **8e** (25.0 mg, 28%) as a pale yellow crystalline solid. m.p. > 230 °C. δ_H (400 MHz, $(CD_3)_2SO$) 1.60 (3H, d, *J* = 6.5 Hz, 5-CHC$\underline{H}_3$), 2.66 (3H, s, 6-CH_3), 3.69 (3H, s, OCH_3), 3.74 (3H, s, 4'-OCH_3), 5.93 (1H, q, *J* = 6.5 Hz, 5-C$\underline{H}$$CH_3$), 6.89 (2H, d, *J* = 8.9 Hz, H-3' and H-5'), 7.37 (2H, br s, NH_2), 7.57 (2H, d, *J* = 8.9 Hz, H-2' and H-6'), 8.53 (1H, s, H-4), 9.29 (1H, br s, NH). δ_C (100 MHz, $(CD_3)_2SO$) 21.1 (5-CH$\underline{C}H_3$), 22.2 (6-CH_3), 54.7 (OCH_3), 55.1 (4'-OCH_3), 72.3 (5-$\underline{C}HCH_3$), 96.2 (C-2), 113.5 (C-3' and C-5'), 122.9 (C-2' and C-6'), 124.6 (C-3a), 128.2 (C-4), 131.4 (C-5), 131.9 (C-1'), 146.6 (C-3), 154.5 (carbonate C=O), 155.5 (C-4'), 156.6 (C-6), 157.4 (C-7a), 163.7 (2-CONH). ν_{max} (ATR)/cm^{-1} 3413 (N-H amide), 3295 (N-H amine), 2955 (C-H aromatic), 2850 (C-H alkane), 1752 (C=O carbonyl), 1601 (C=O amide), 1507 (C=C aromatic), 1464 (-C-H bending), 1263 (C-N aromatic), 1106 (C-O ether), 1071 (C-N aliphatic). *m/z* (ESI^+): 416 (MH^+, 100%). HRMS (ESI^+) found (MH^+): 416.1265 $C_{20}H_{22}N_3O_5S$ requires 416.1275.

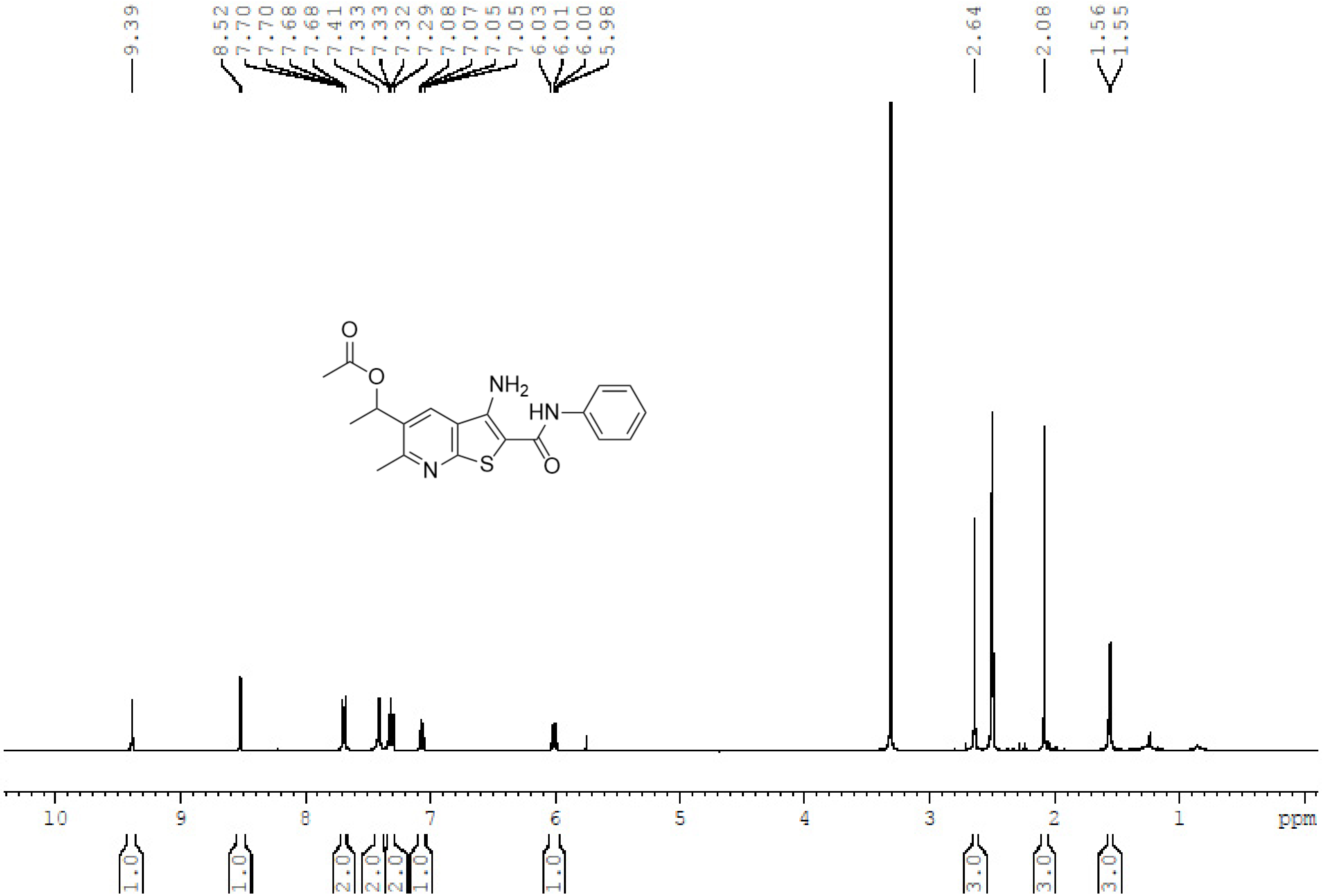

Figure S1: ^{1}H NMR spectrum of **6a** (400 MHz; DMSO-d_6).

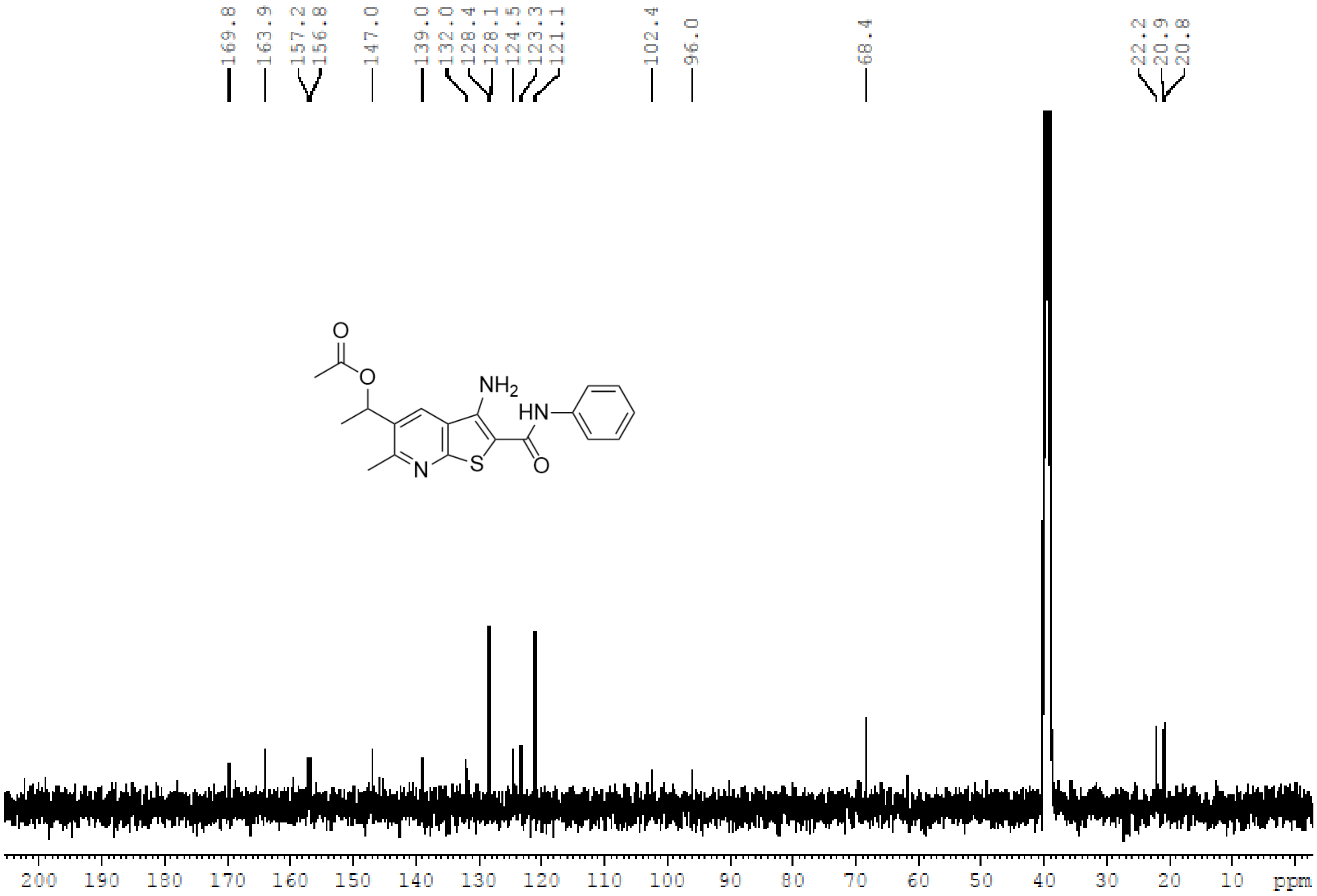

Figure S2: ^{13}C NMR spectrum of **6a** (100 MHz; DMSO-d_6).

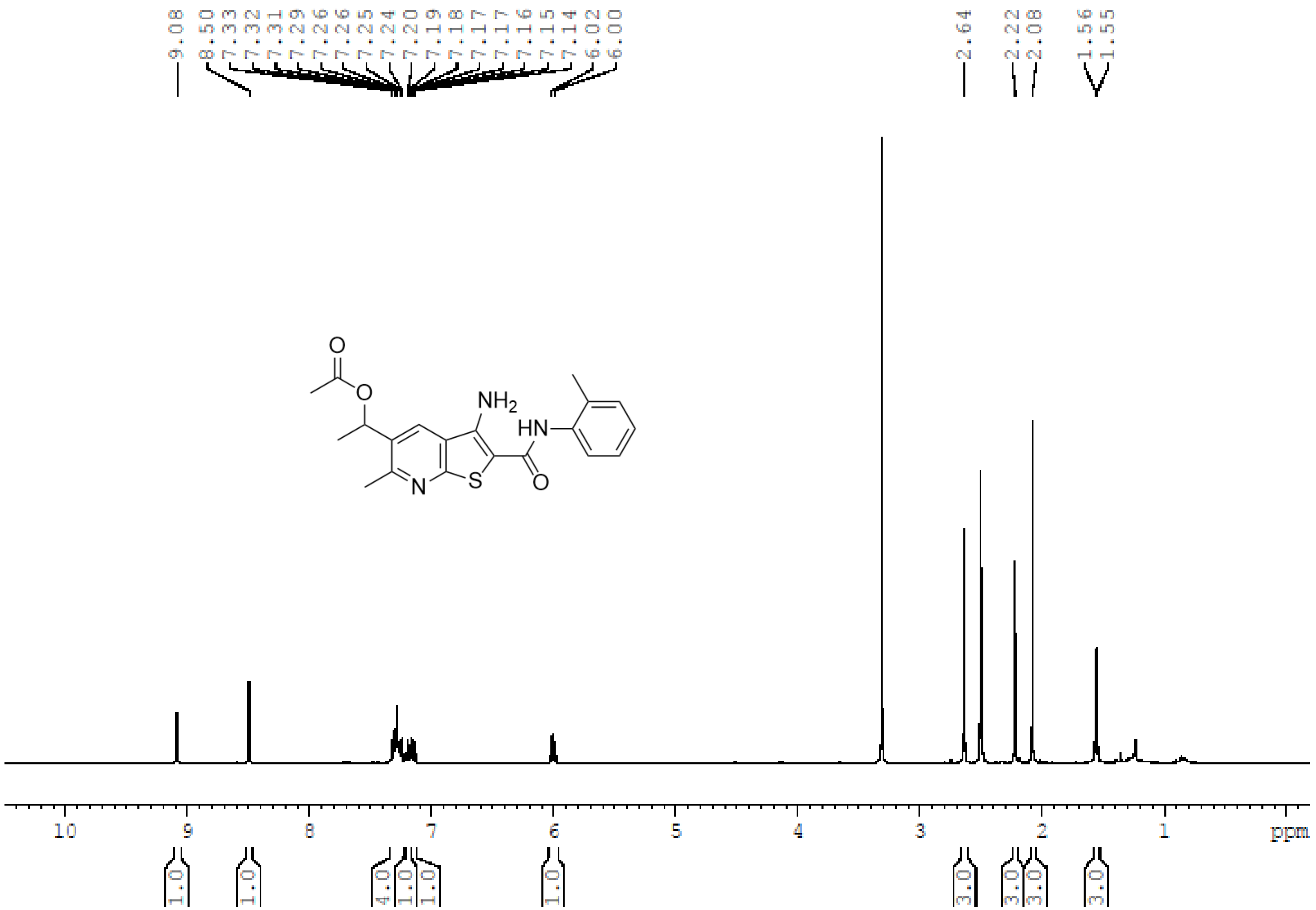

Figure S3: ^{1}H NMR spectrum of **6b** (400 MHz; DMSO-d_6).

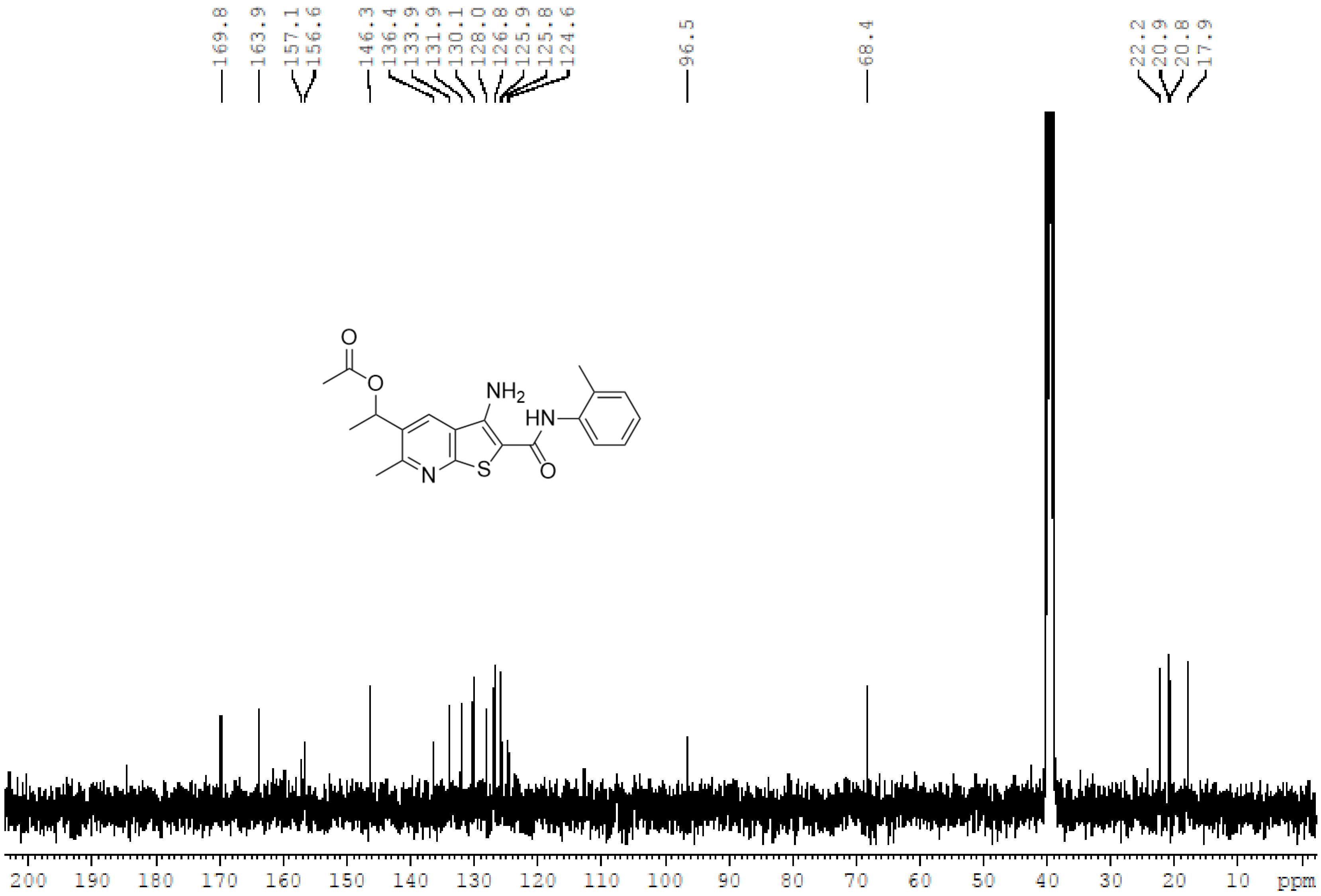

Figure S4: ^{13}C NMR spectrum of **6b** (100 MHz; DMSO-*d₆*).

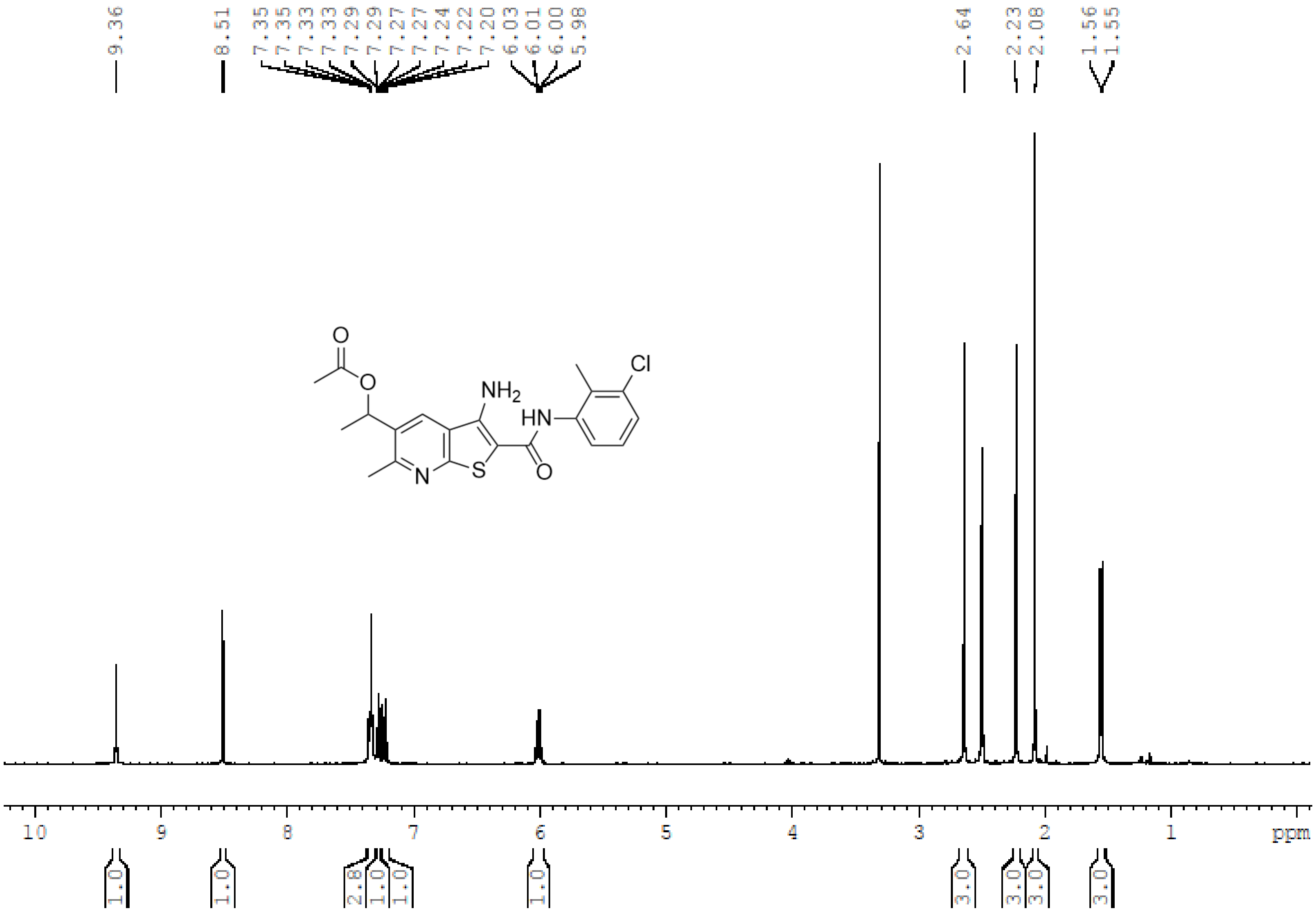

Figure S5: ^{1}H NMR spectrum of **6c** (400 MHz; DMSO-d_6).

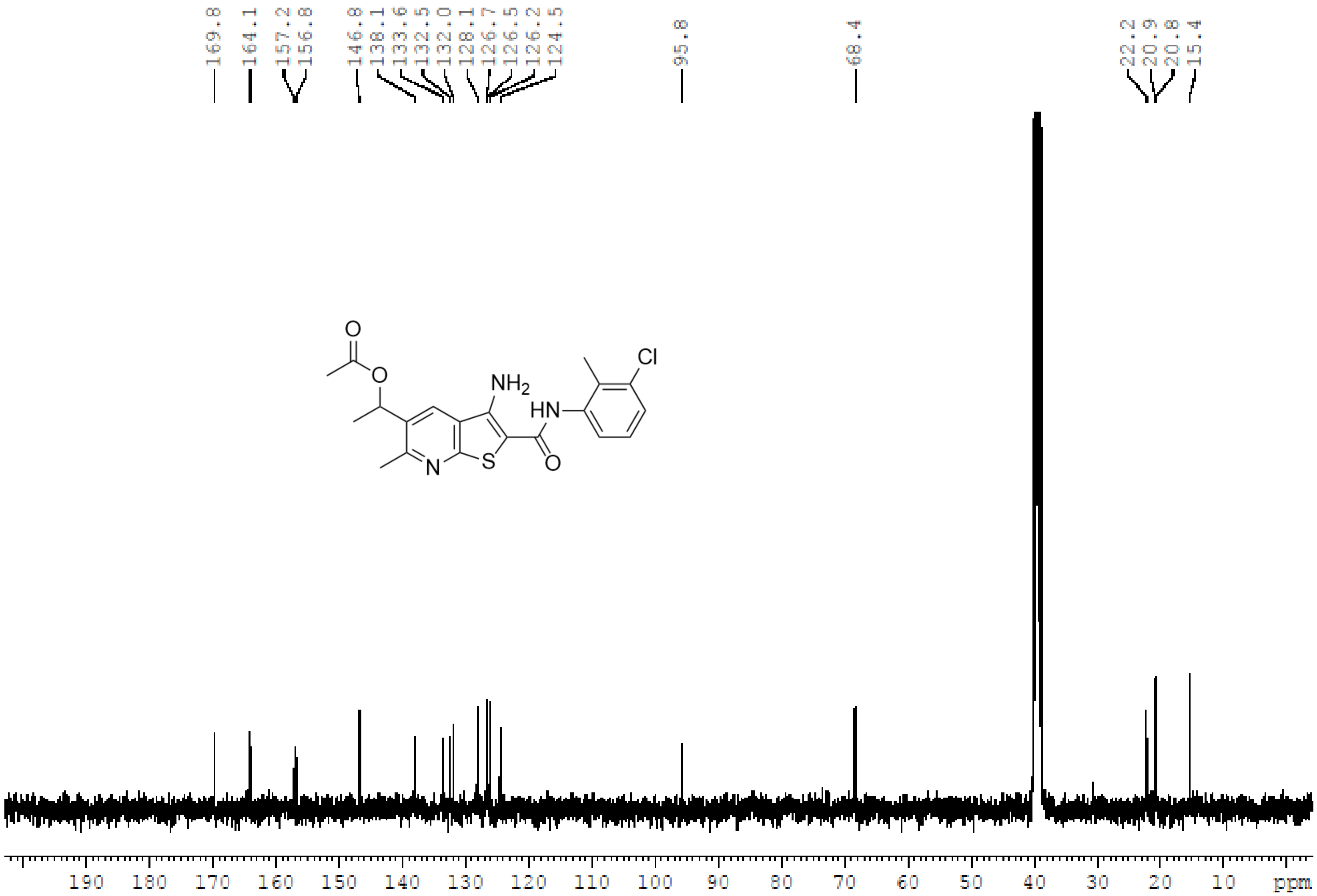

Figure S6: ^{13}C NMR spectrum of **6c** (100 MHz; DMSO-*d6*).

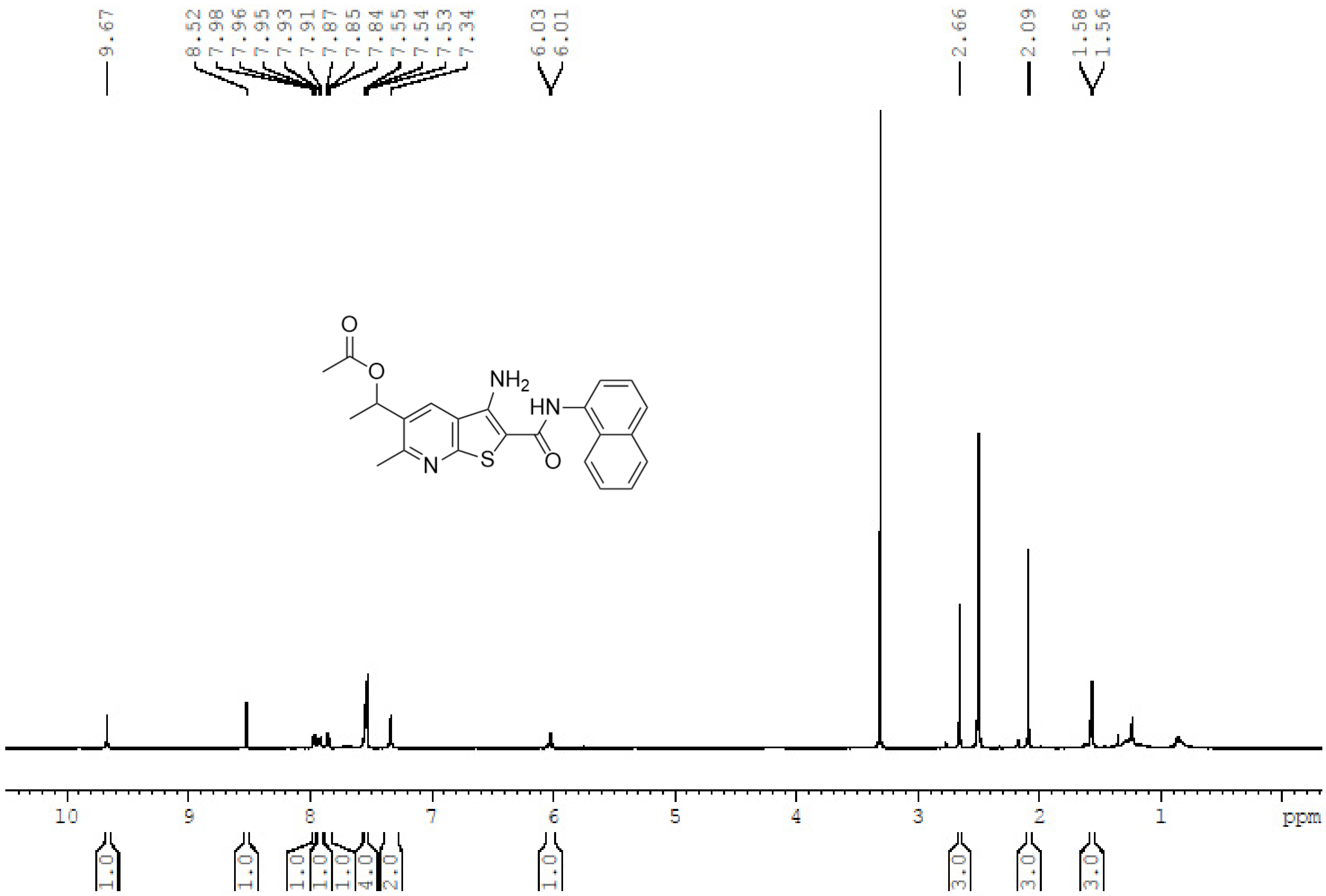

Figure S7: ^{1}H NMR spectrum of **6d** (400 MHz; DMSO-*d*$_6$).

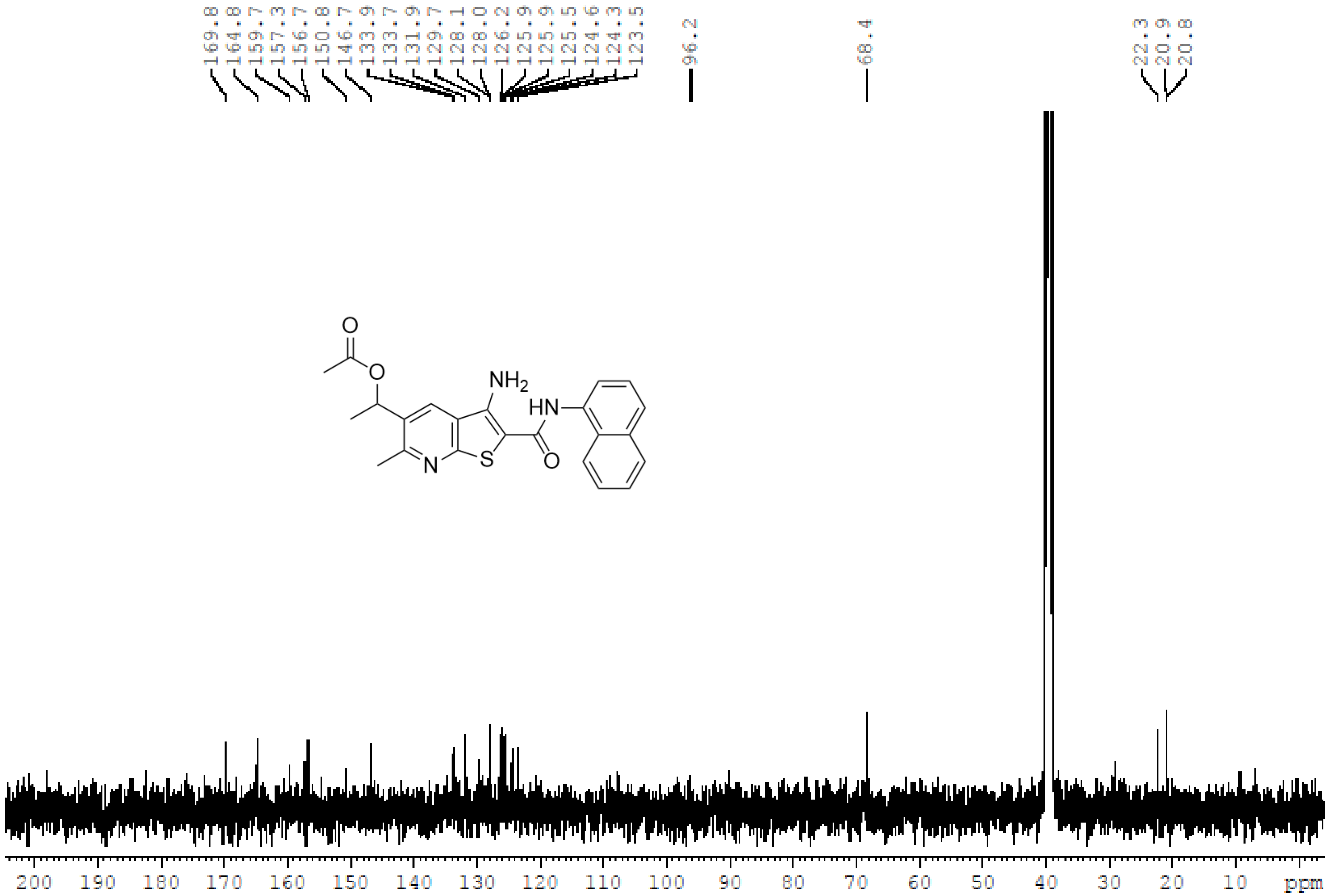

Figure S8: ^{13}C NMR spectrum of **6d** (100 MHz; DMSO-d_6).

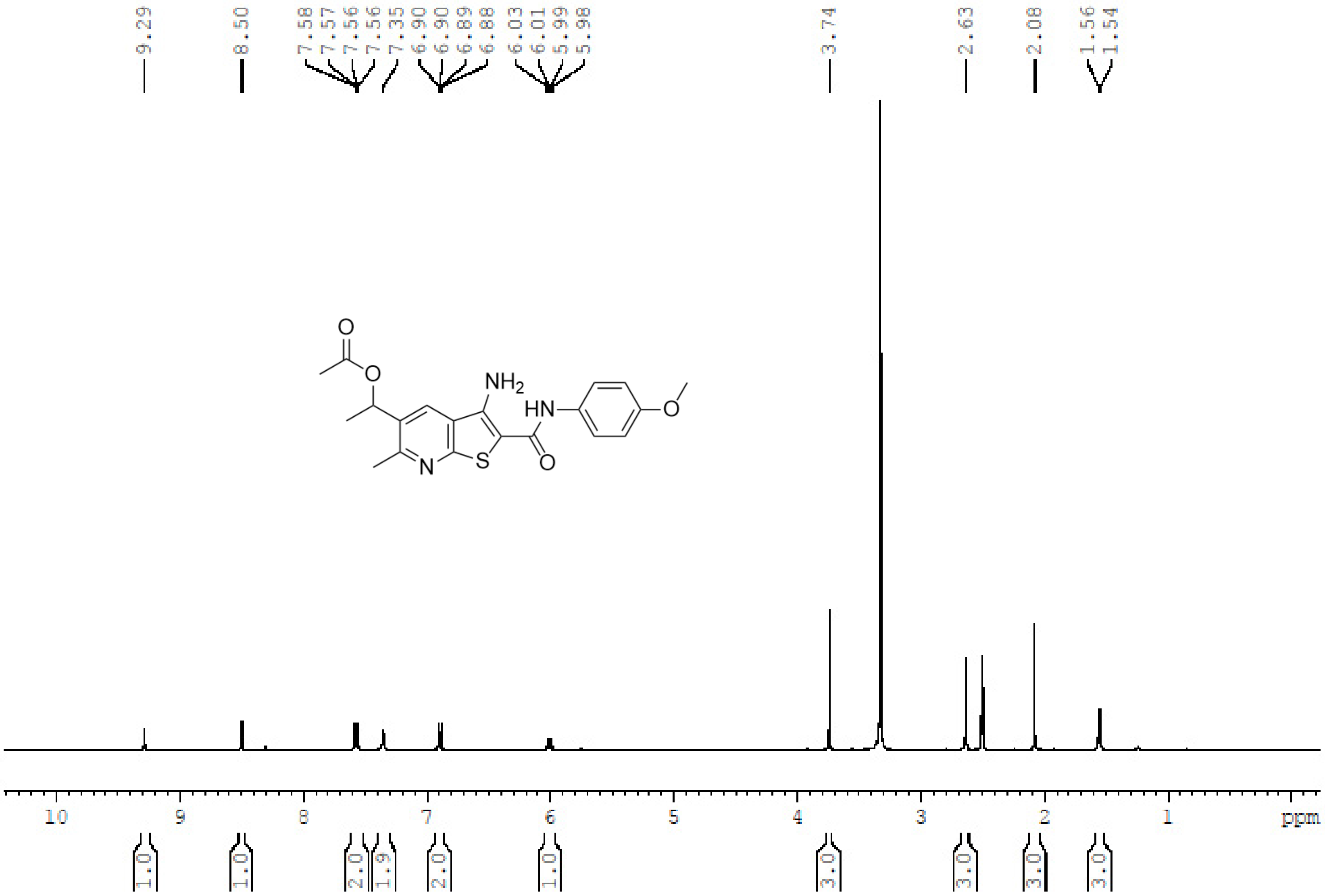

Figure S9: ^{1}H NMR spectrum of **6e** (400 MHz; DMSO-*d*$_6$).

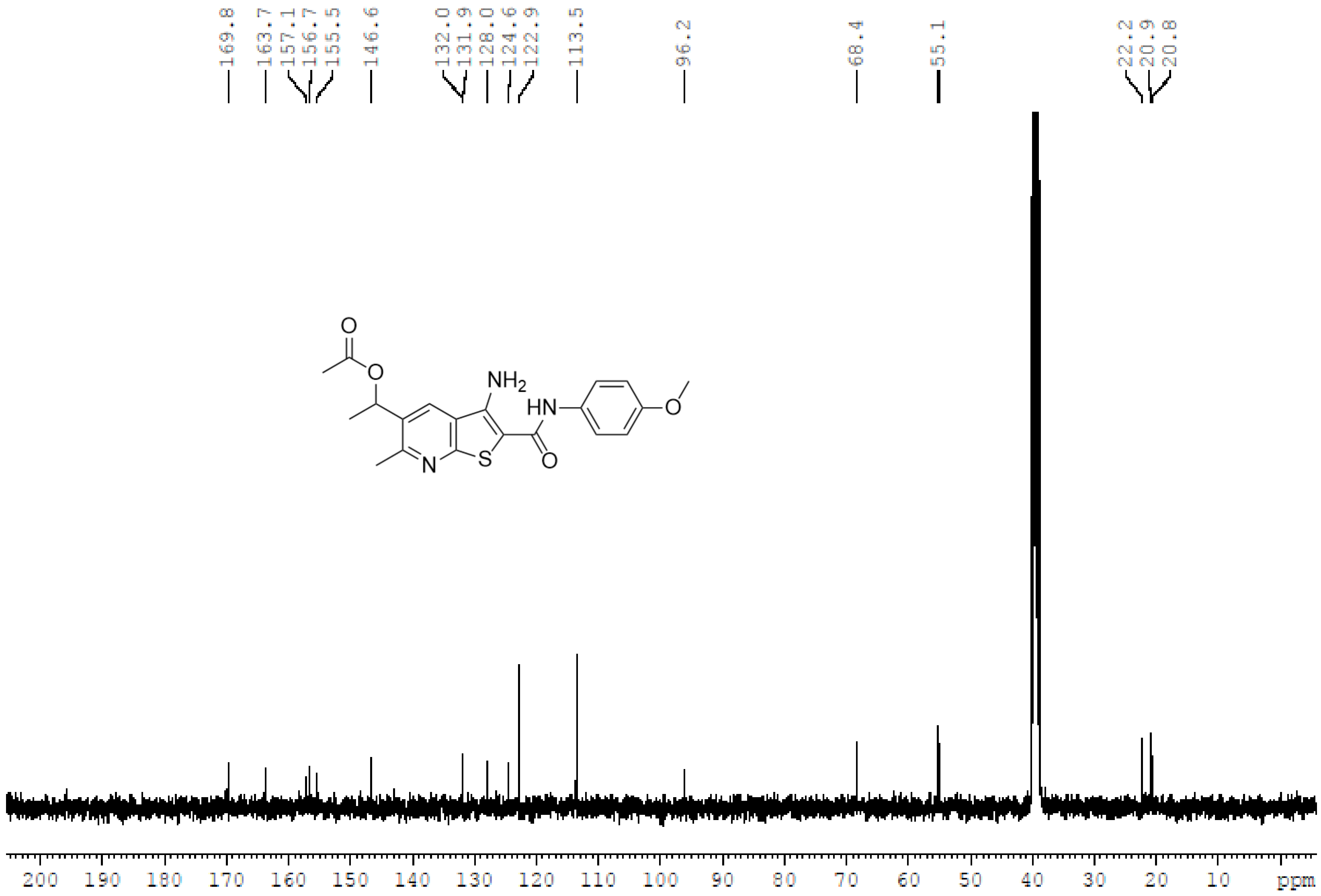

Figure S10: ^{13}C NMR spectrum of **6e** (100 MHz; DMSO-*d*$_6$).

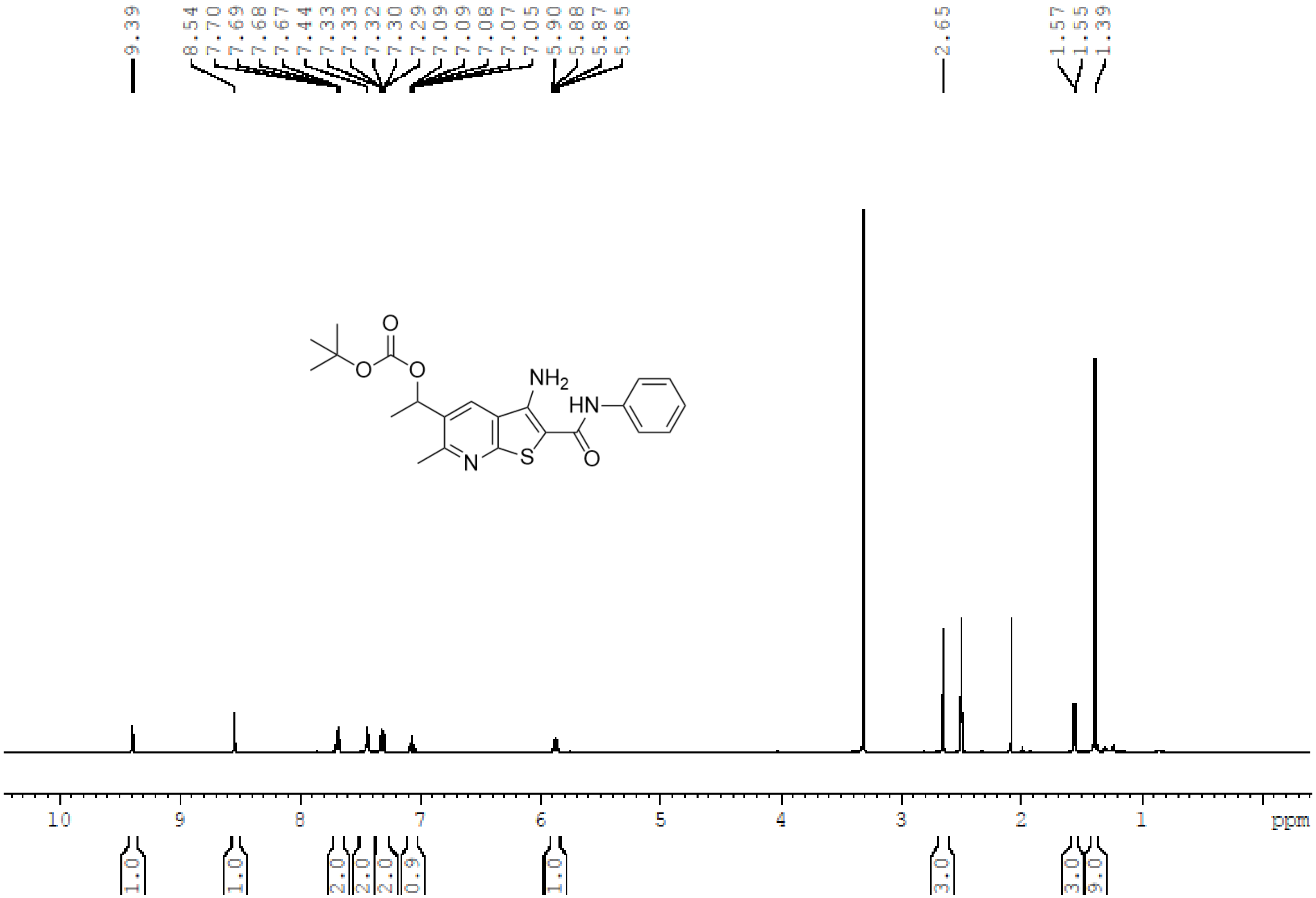

Figure S11: ^{1}H NMR spectrum of **7a** (400 MHz; DMSO-*d*$_6$).

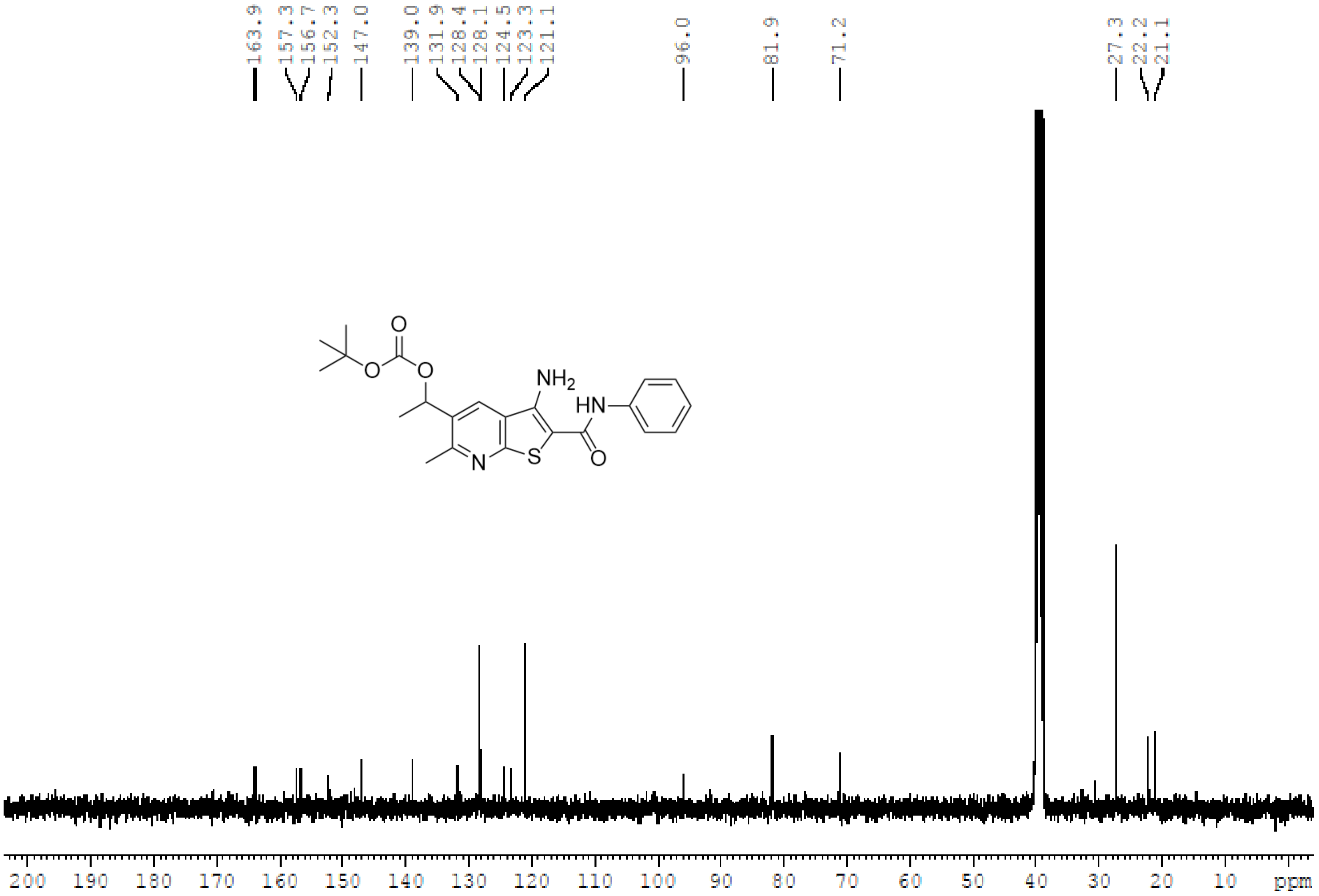

Figure S12: ^{13}C NMR spectrum of **7a** (100 MHz; DMSO-*d*$_6$).

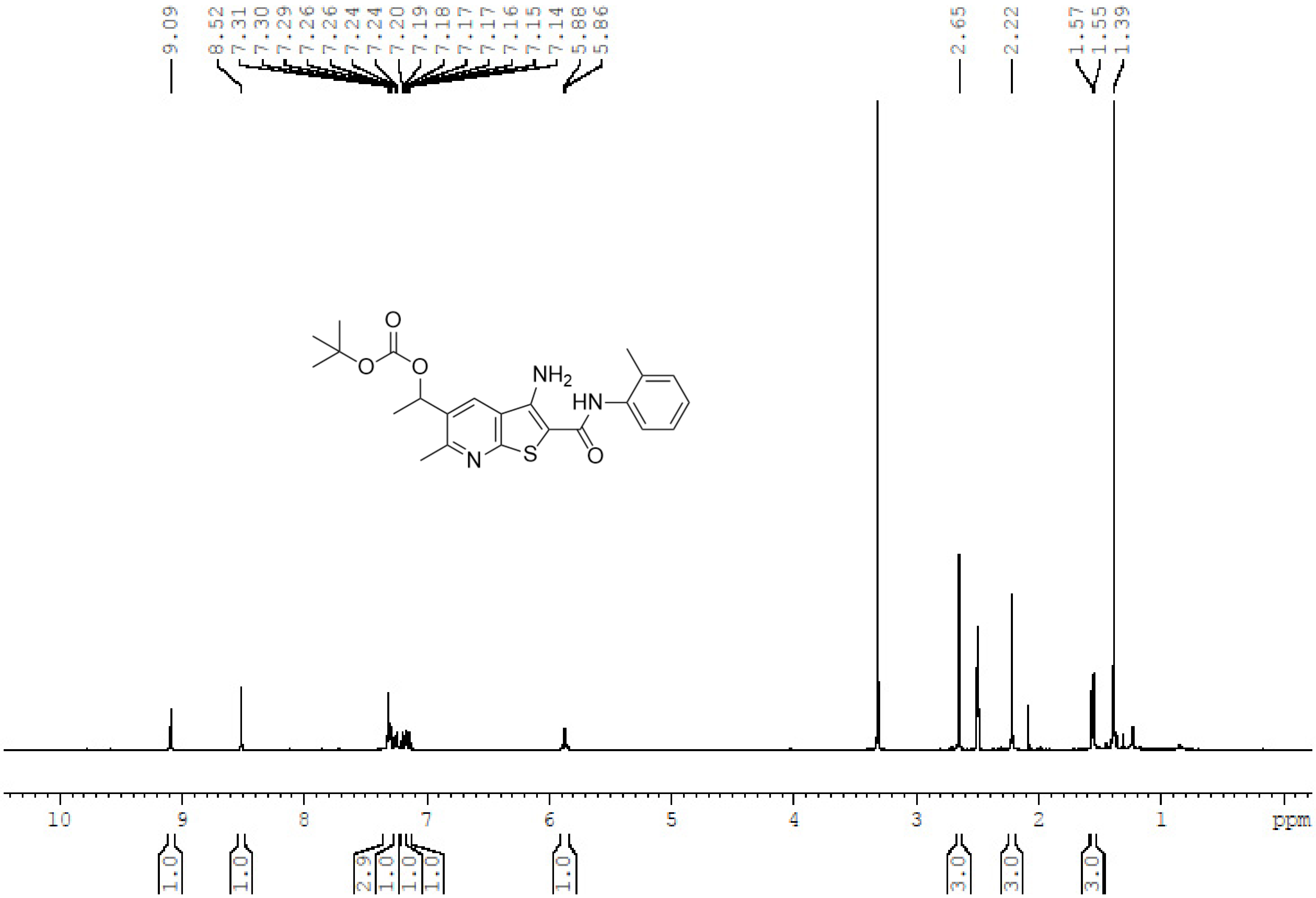

Figure S13: ^{1}H NMR spectrum of **7b** (400 MHz; DMSO-d_6).

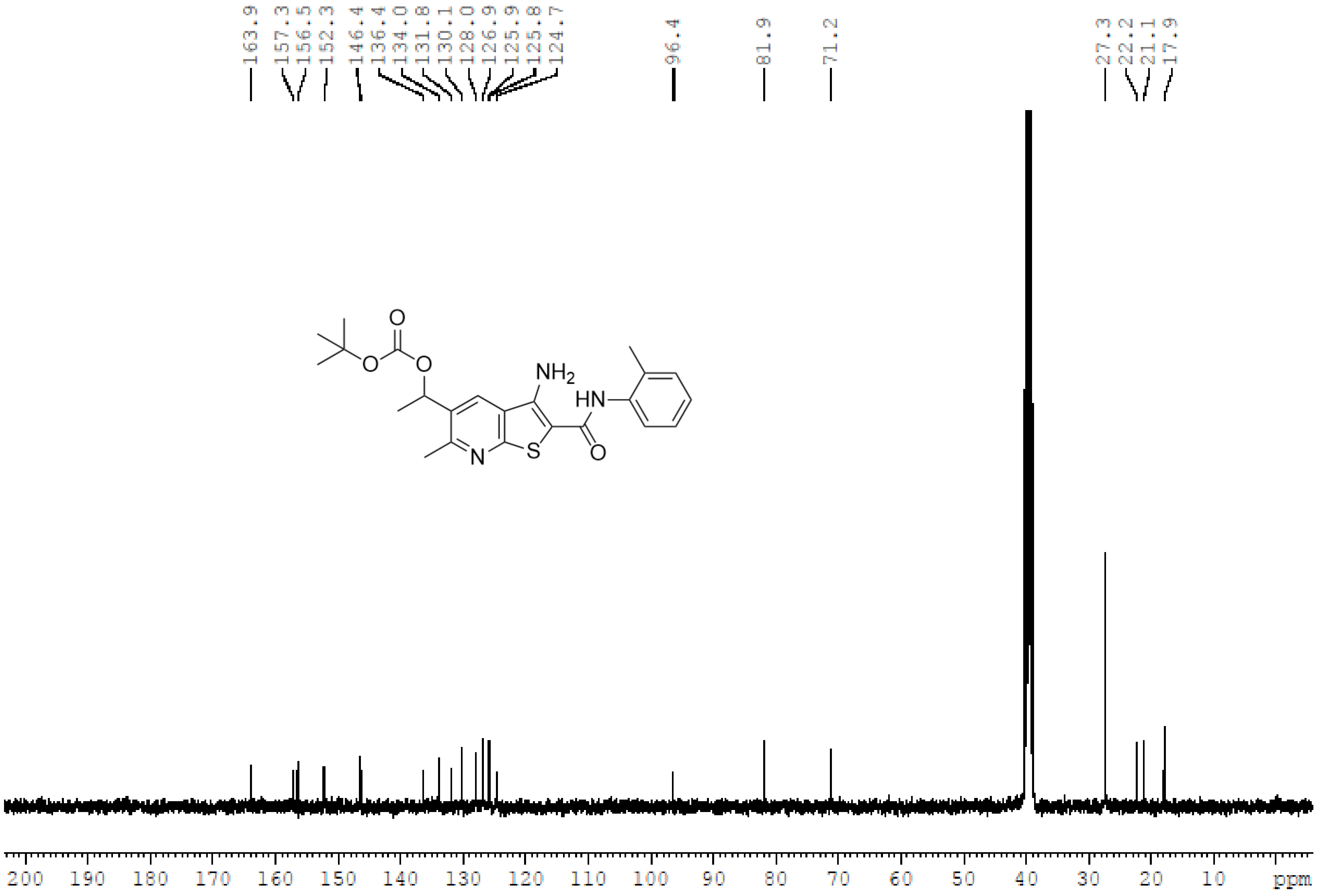

Figure S14: ^{13}C NMR spectrum of **7b** (100 MHz; DMSO-*d₆*).

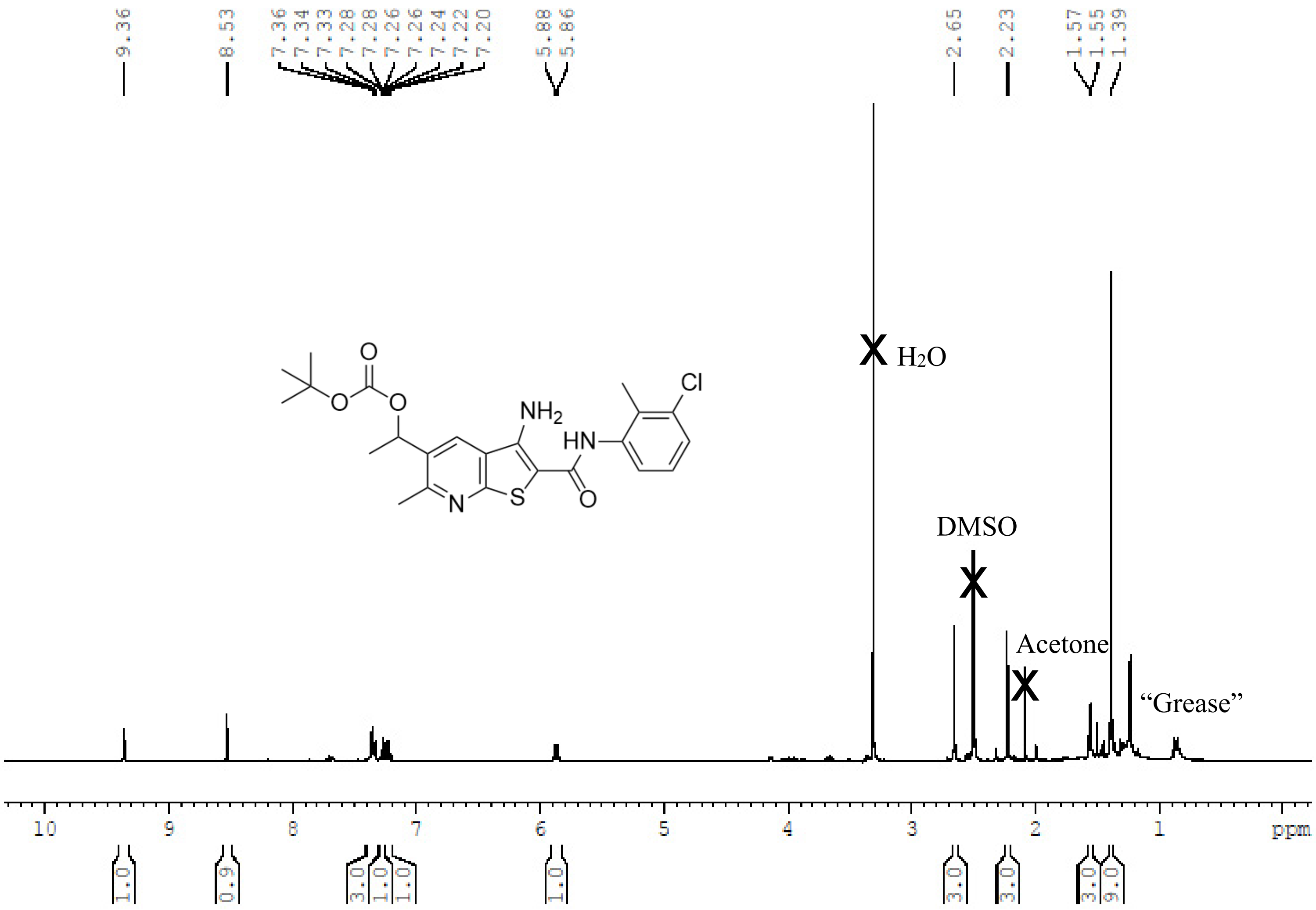

Figure S15: ^{1}H NMR spectrum of **7c** (400 MHz; DMSO-d_6).

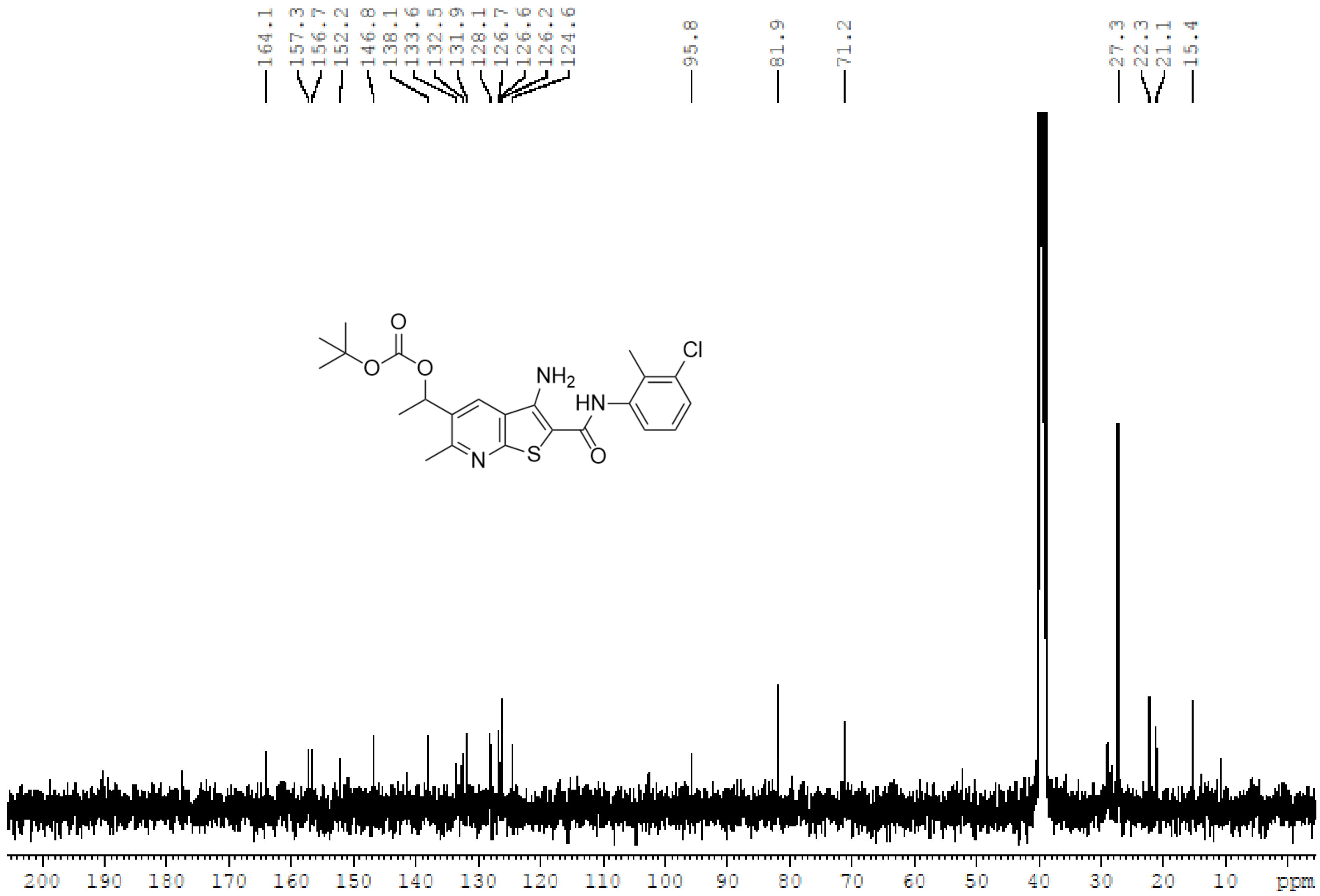

Figure S16: ^{13}C NMR spectrum of **7c** (100 MHz; DMSO-*d*$_6$).

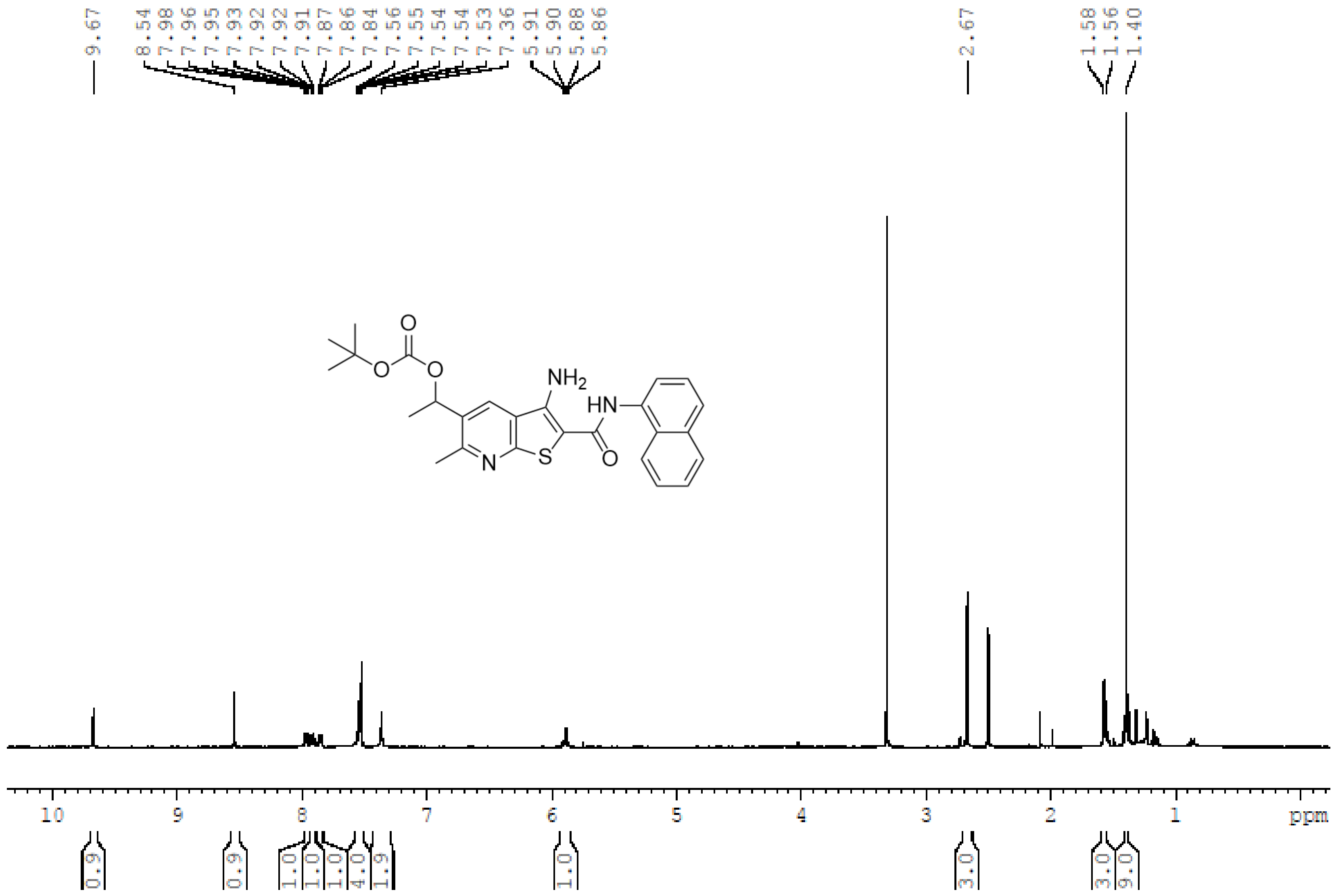

Figure S17: ^{1}H NMR spectrum of **7d** (400 MHz; DMSO-d_6).

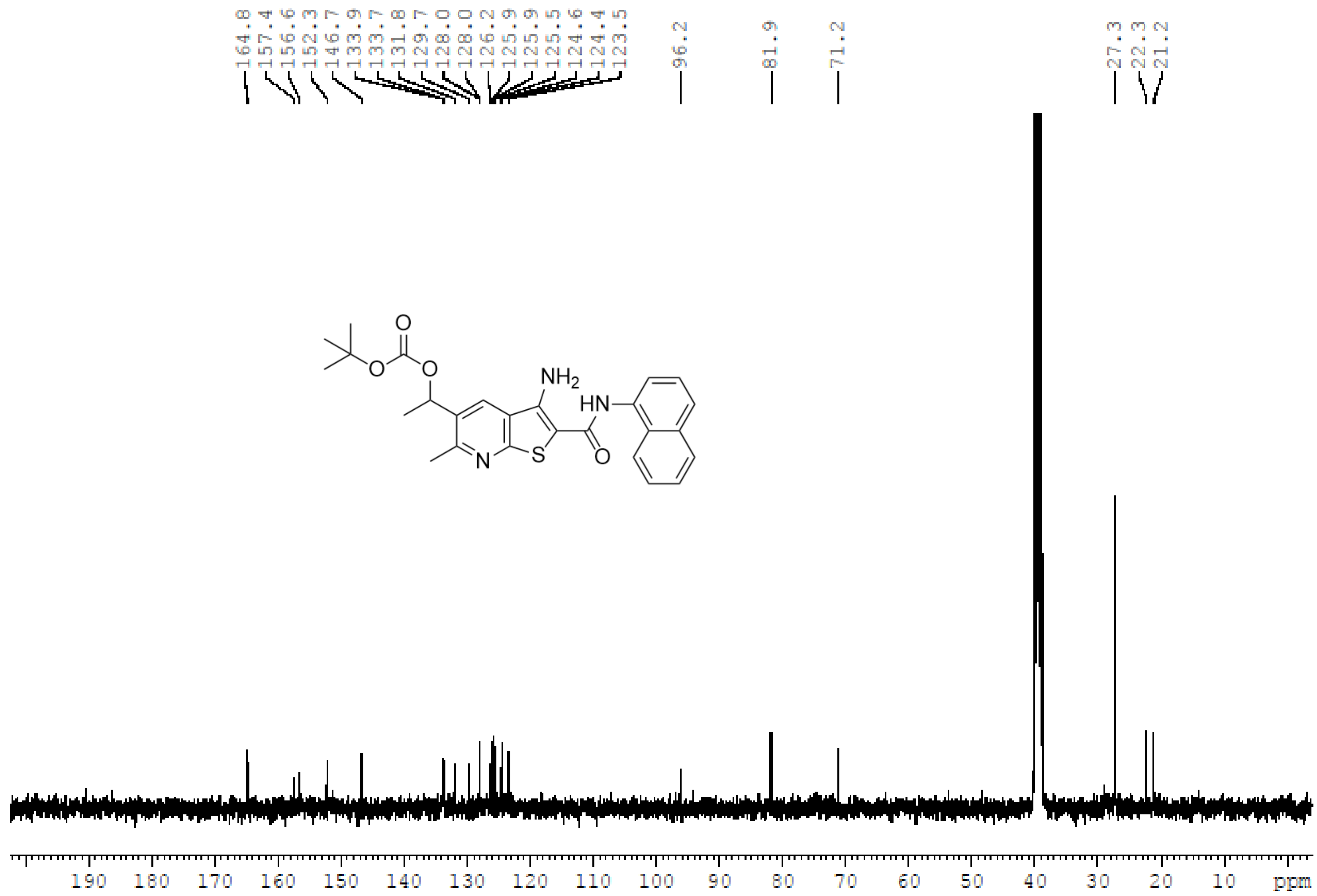

Figure S18: ^{13}C NMR spectrum of **7d** (100 MHz; DMSO-d_6).

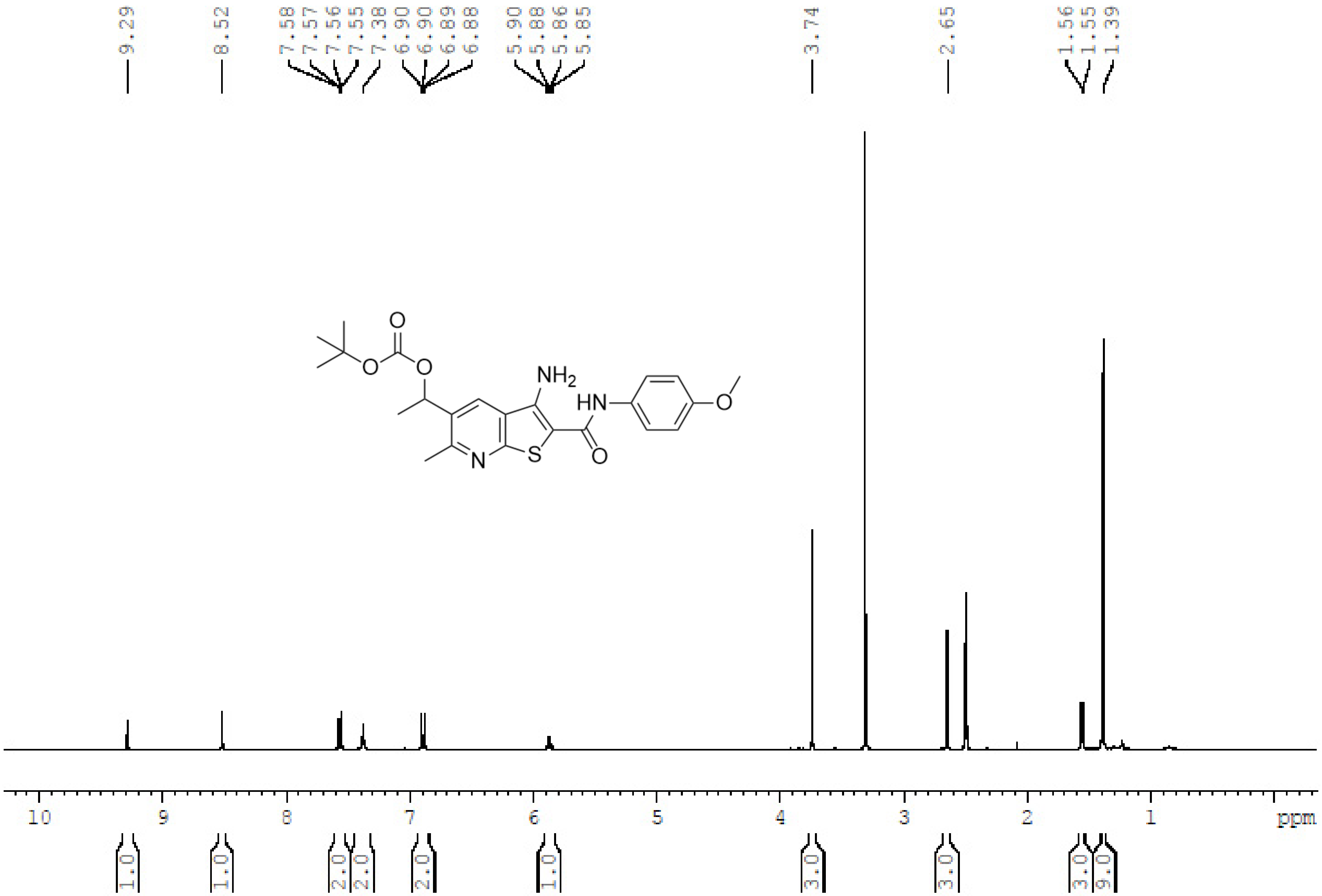

Figure S19: ^{1}H NMR spectrum of **7e** (400 MHz; DMSO-*d*$_6$).

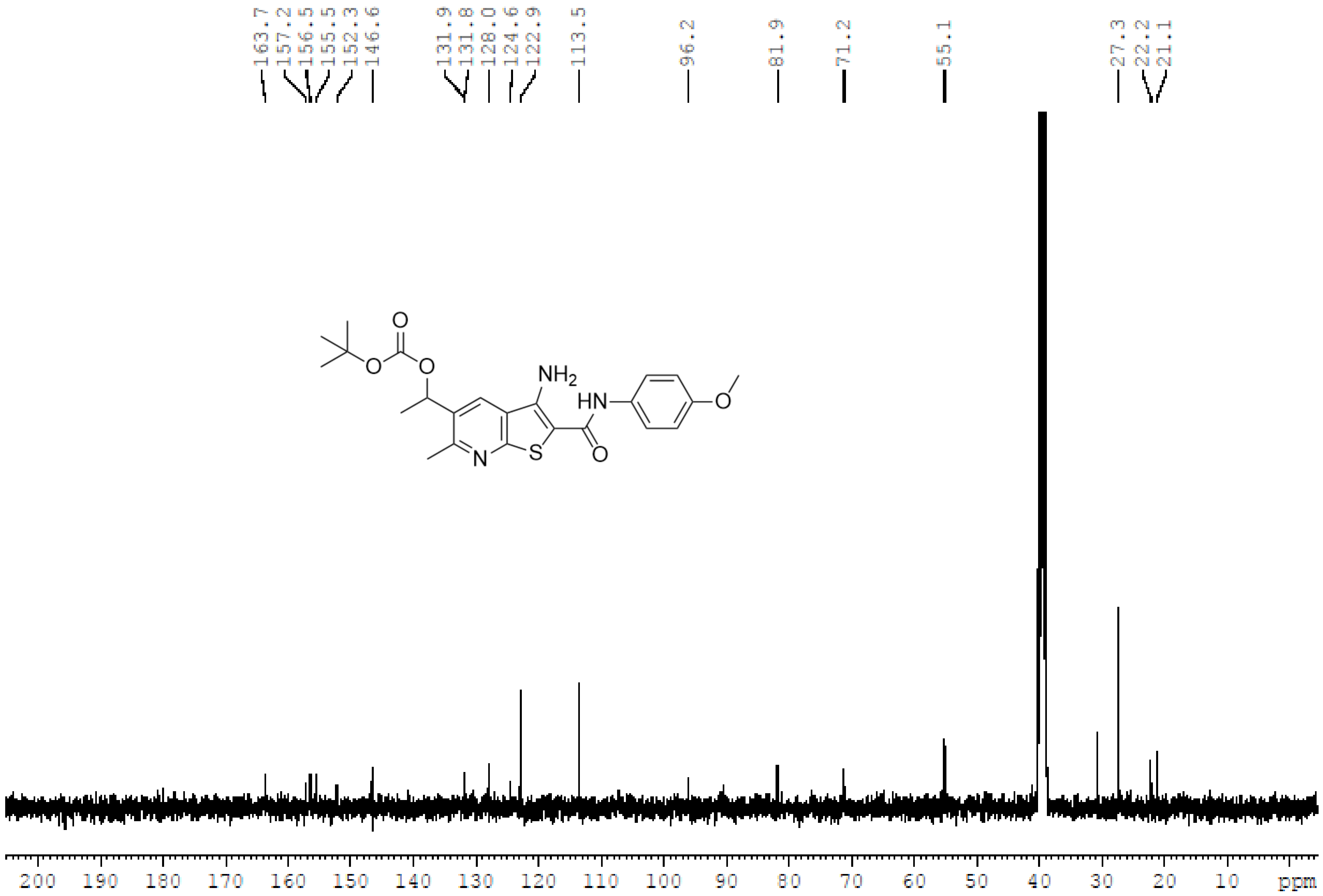

Figure S20: ^{13}C NMR spectrum of **7e** (100 MHz; DMSO-*d*$_6$).

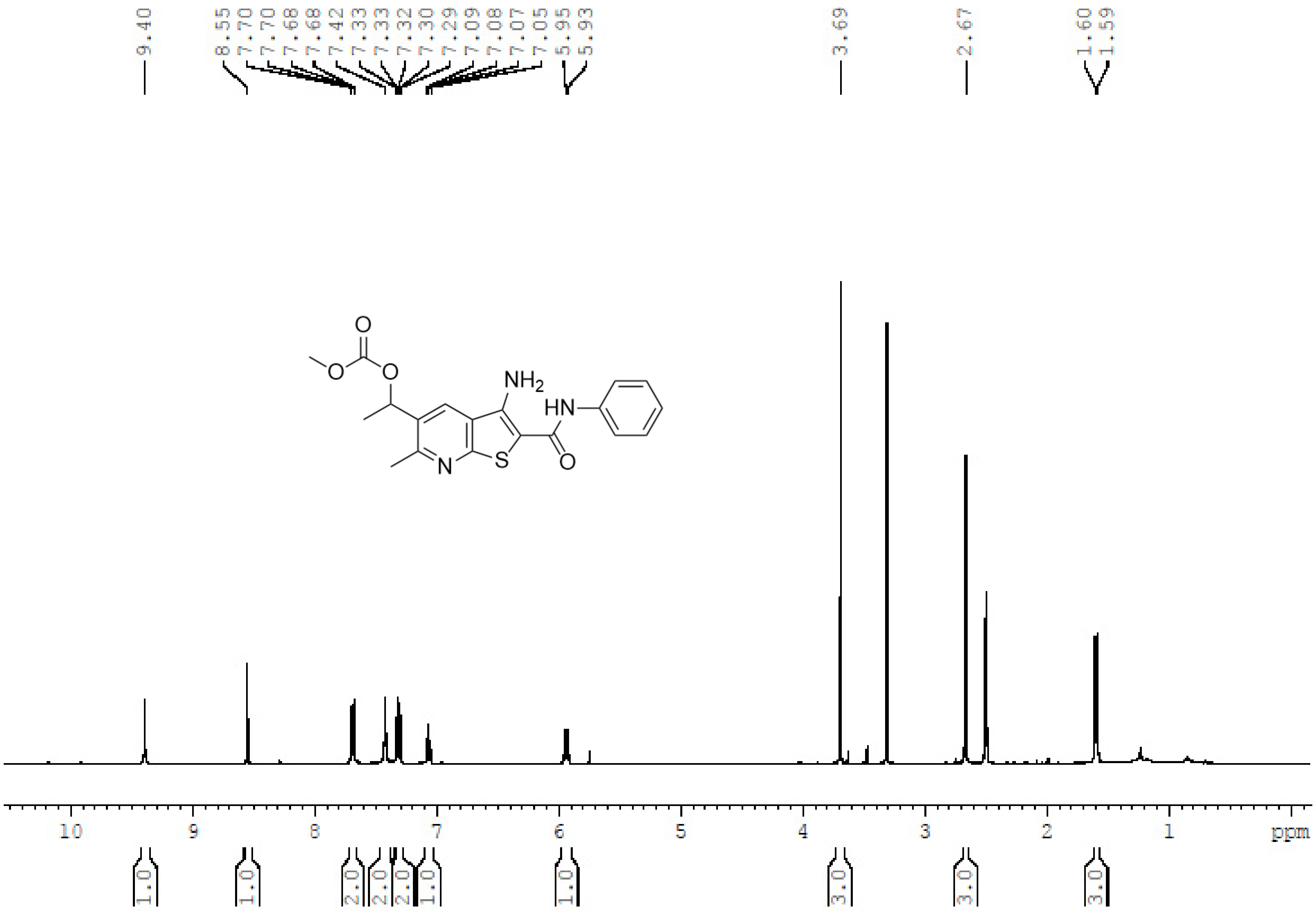

Figure S21: ^{1}H NMR spectrum of **8a** (400 MHz; DMSO-d_6).

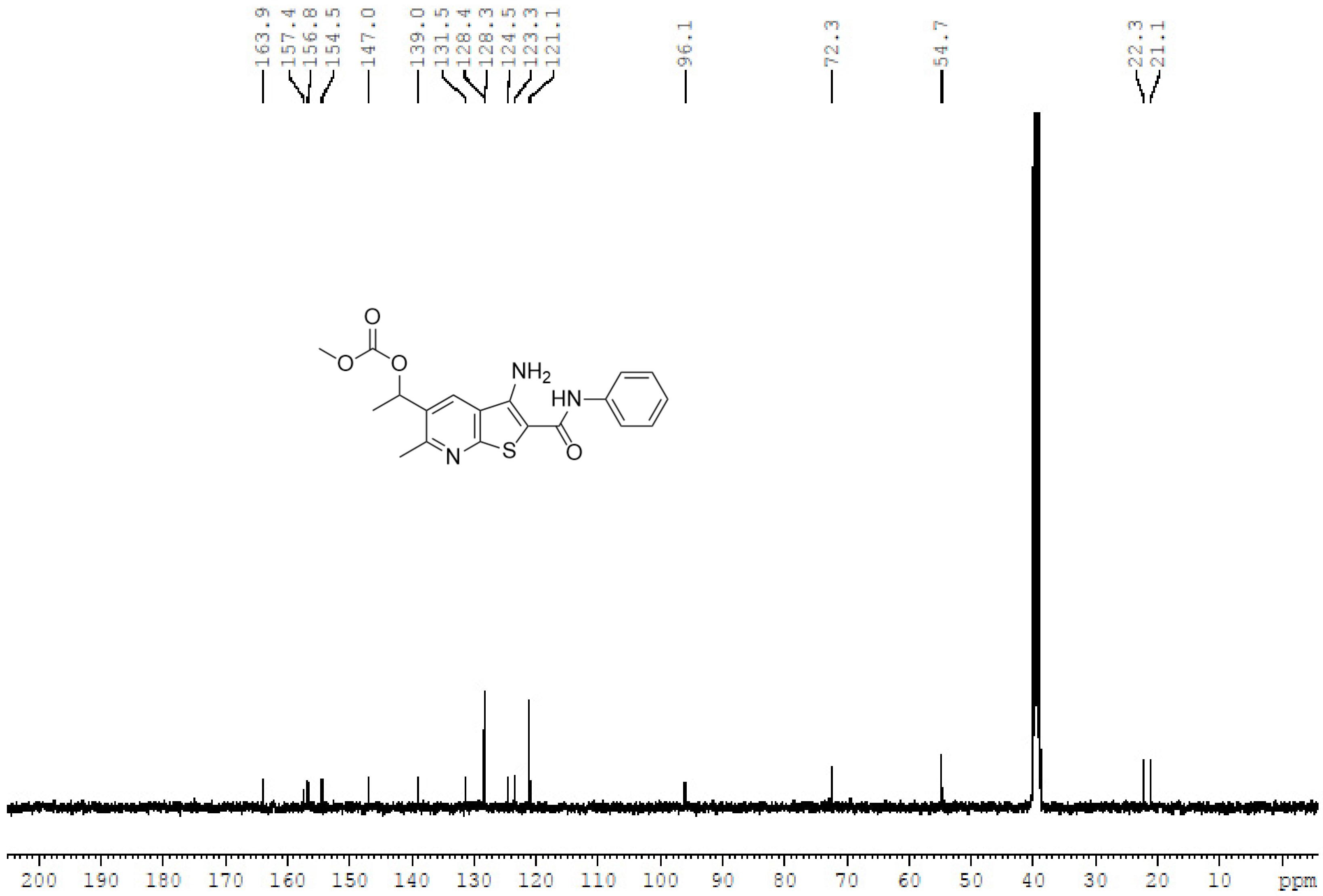

Figure S22: ^{13}C NMR spectrum of **8a** (100 MHz; DMSO-d_6).

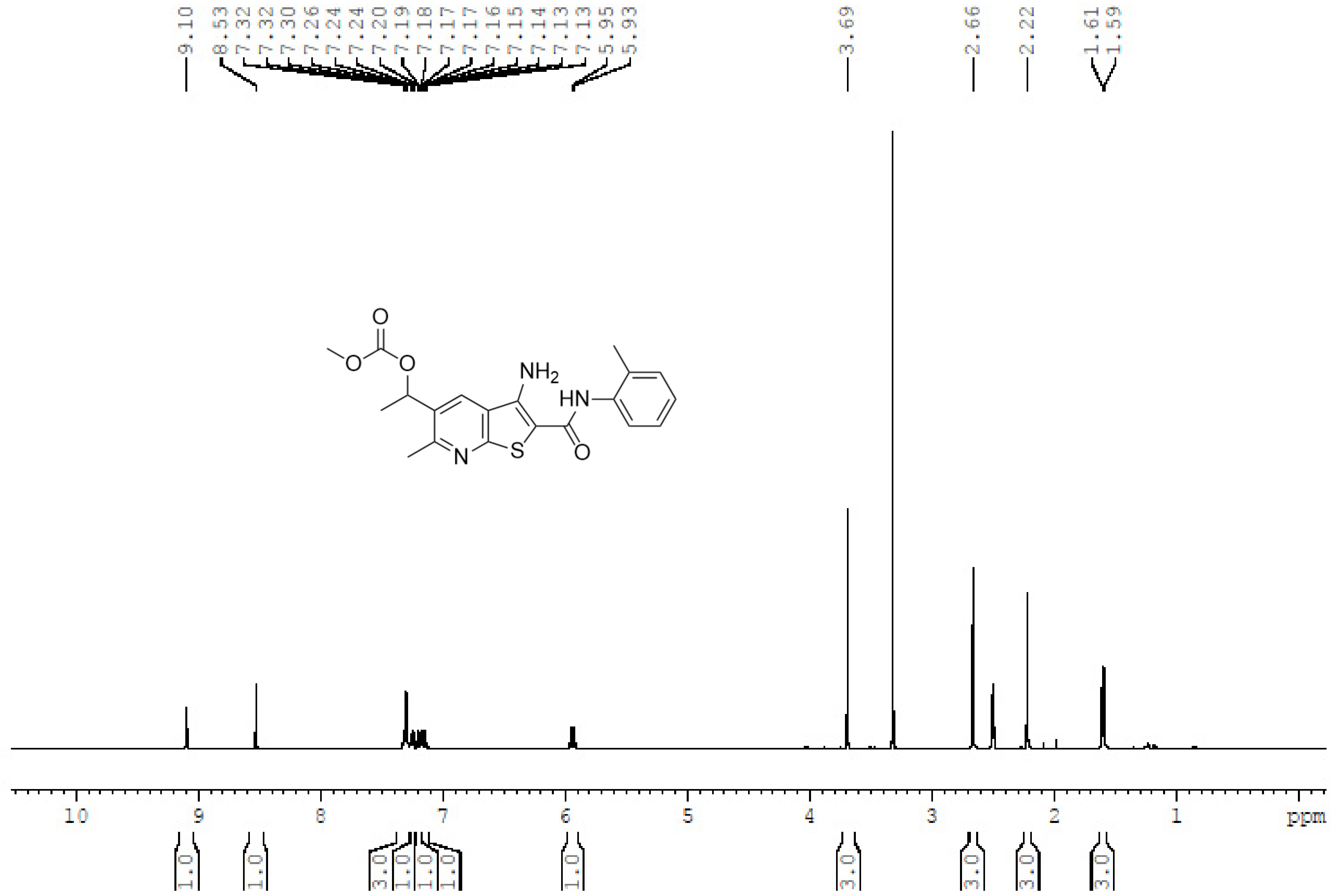

Figure S23: ^{1}H NMR spectrum of **8b** (400 MHz; DMSO-d_6).

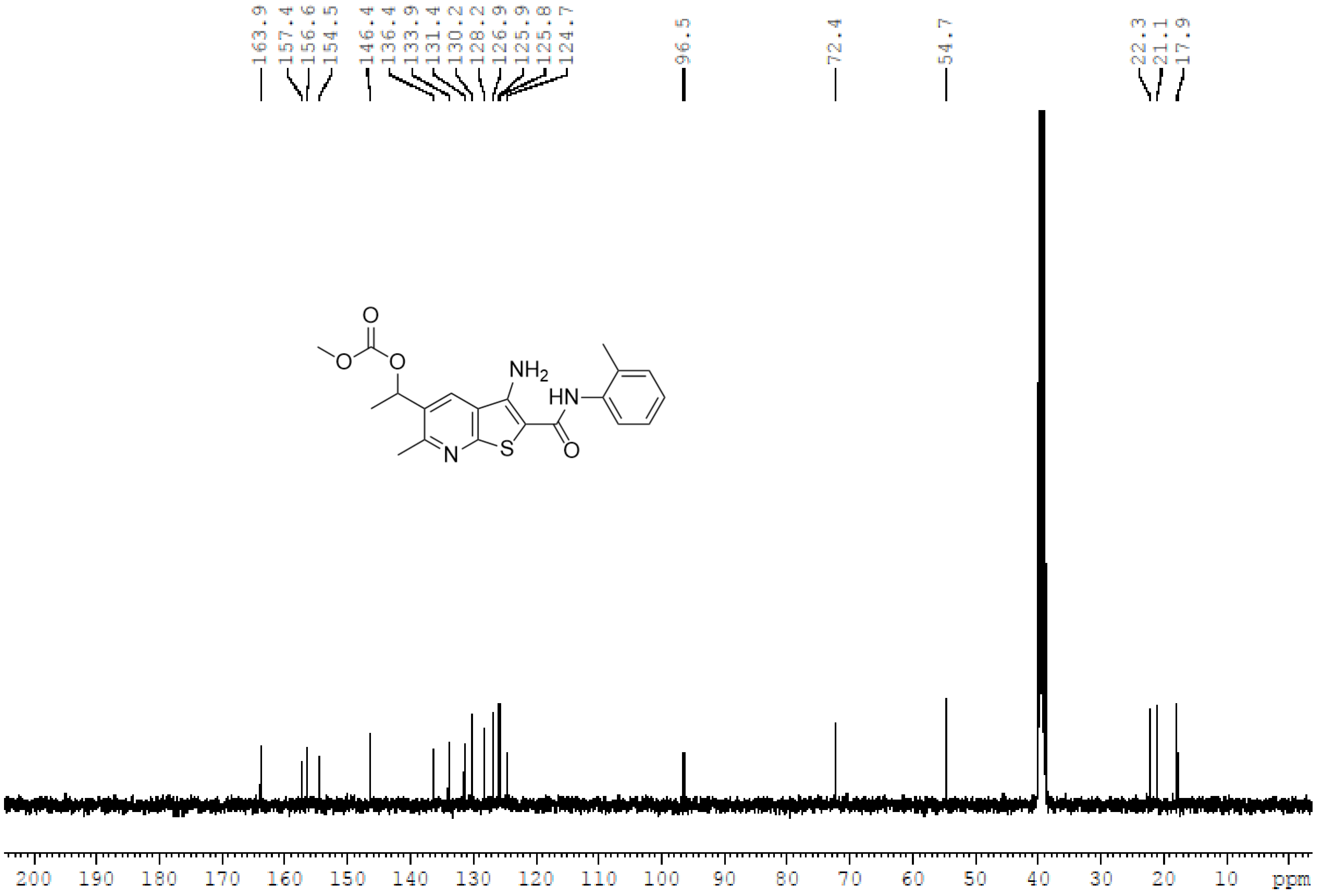

Figure S24: ^{13}C NMR spectrum of **8b** (100 MHz; DMSO-d_6).

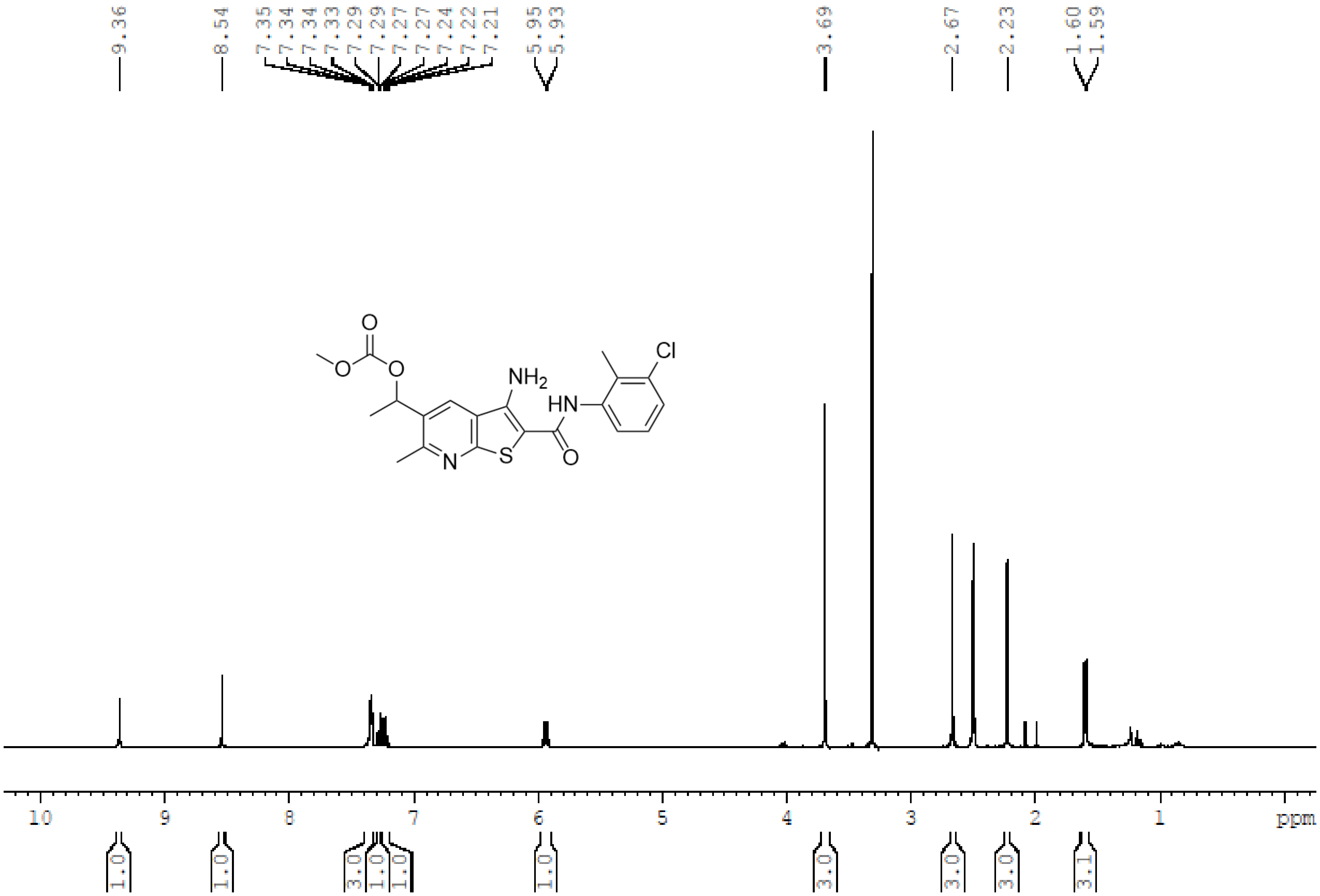

Figure S25: ^{1}H NMR spectrum of **8c** (400 MHz; DMSO-d_6).

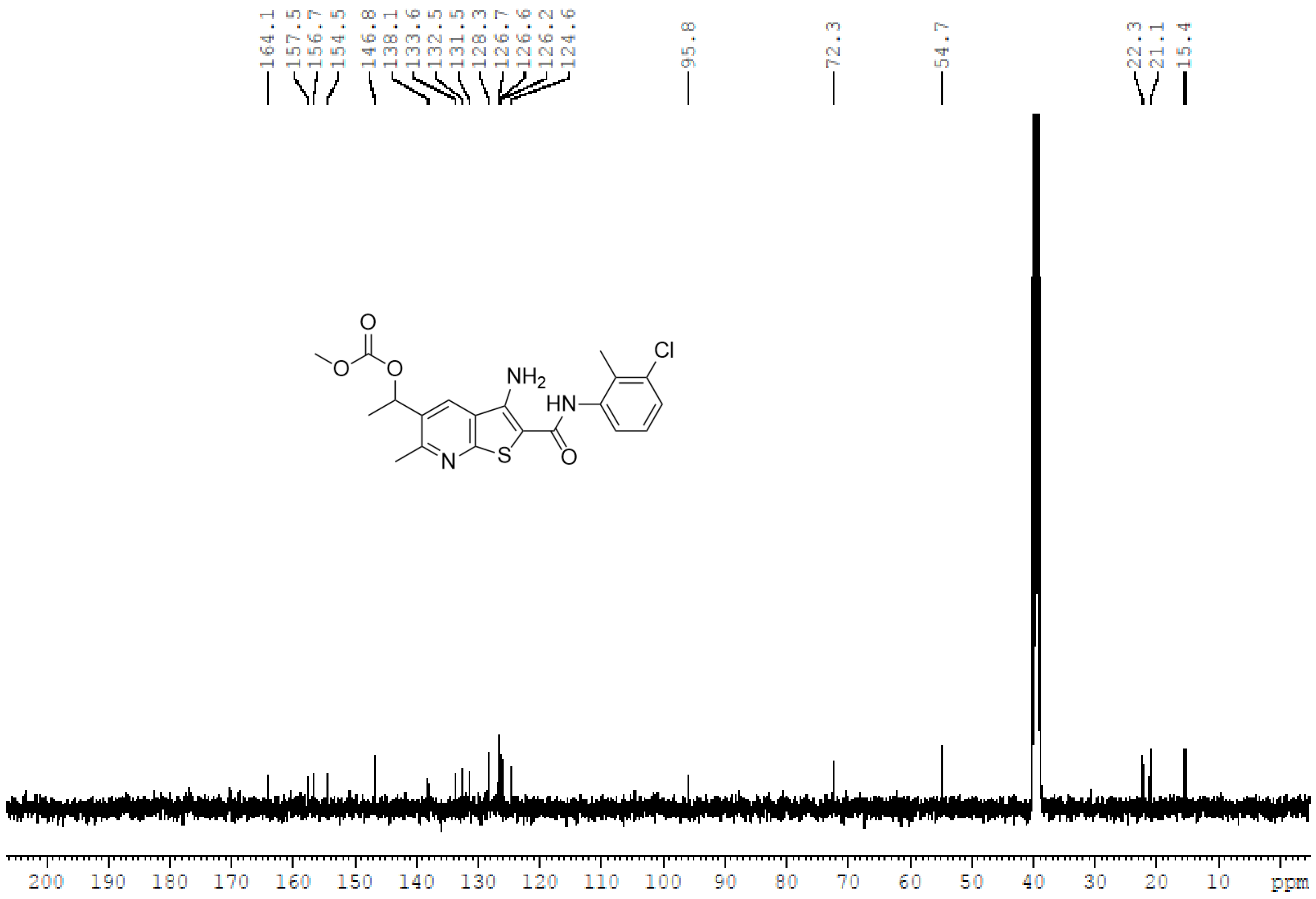

Figure S26: ^{13}C NMR spectrum of **8c** (100 MHz; DMSO-d_6).

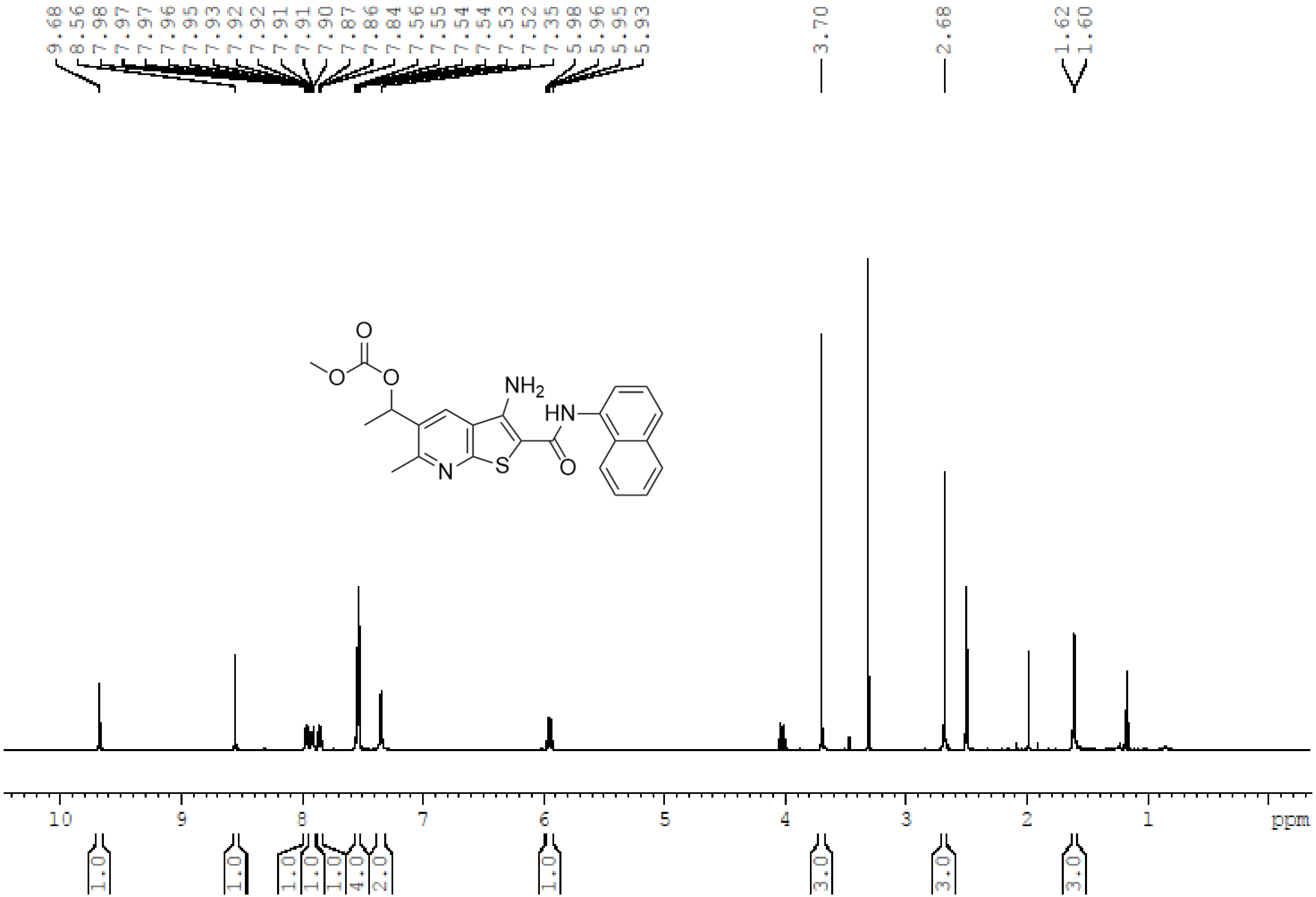

Figure S27: ^{1}H NMR spectrum of **8d** (400 MHz; DMSO-d_6).

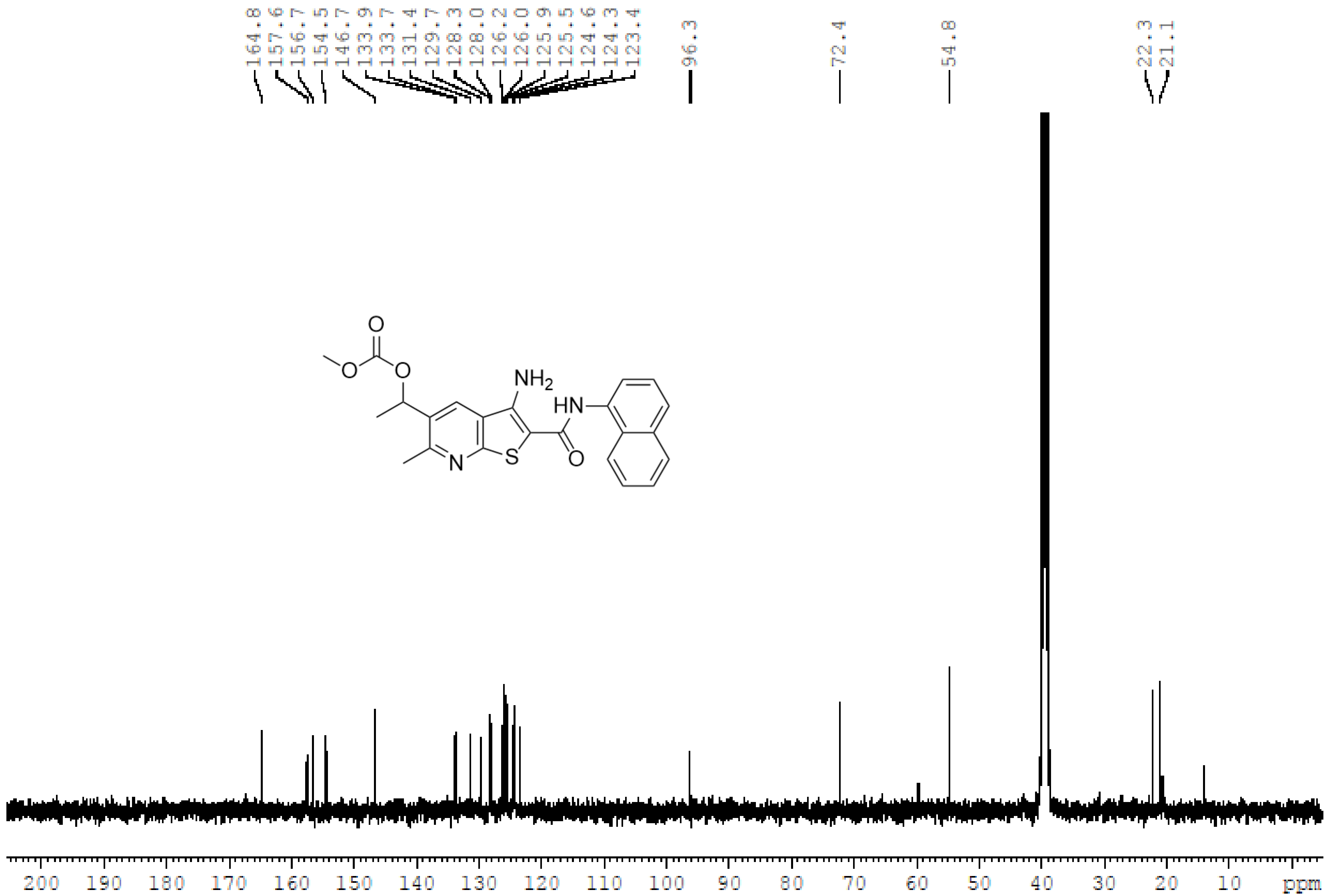

Figure S28: ^{13}C NMR spectrum of **8d** (100 MHz; DMSO-d_6).

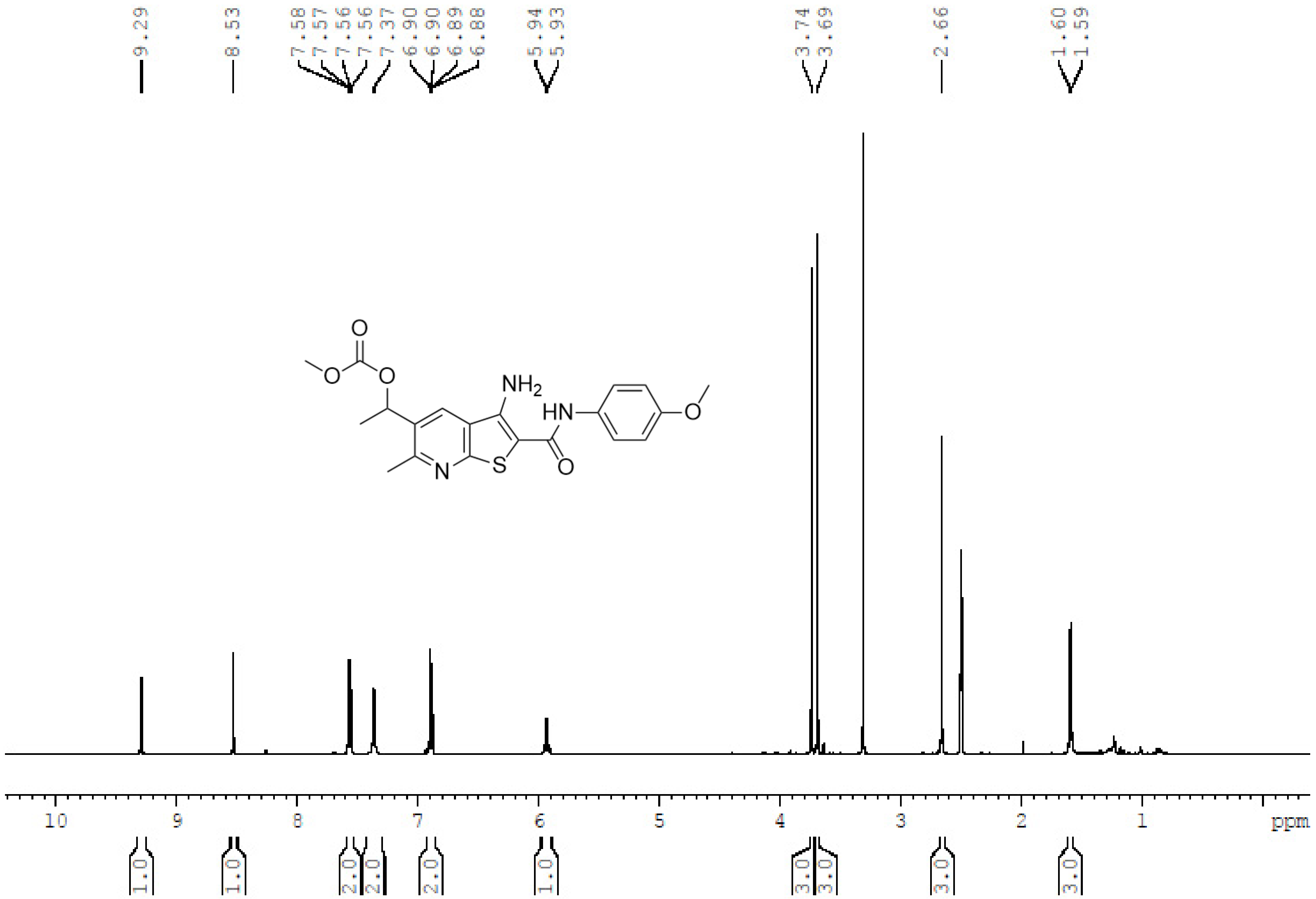

Figure S29: ^{1}H NMR spectrum of **8e** (400 MHz; DMSO-d_6).

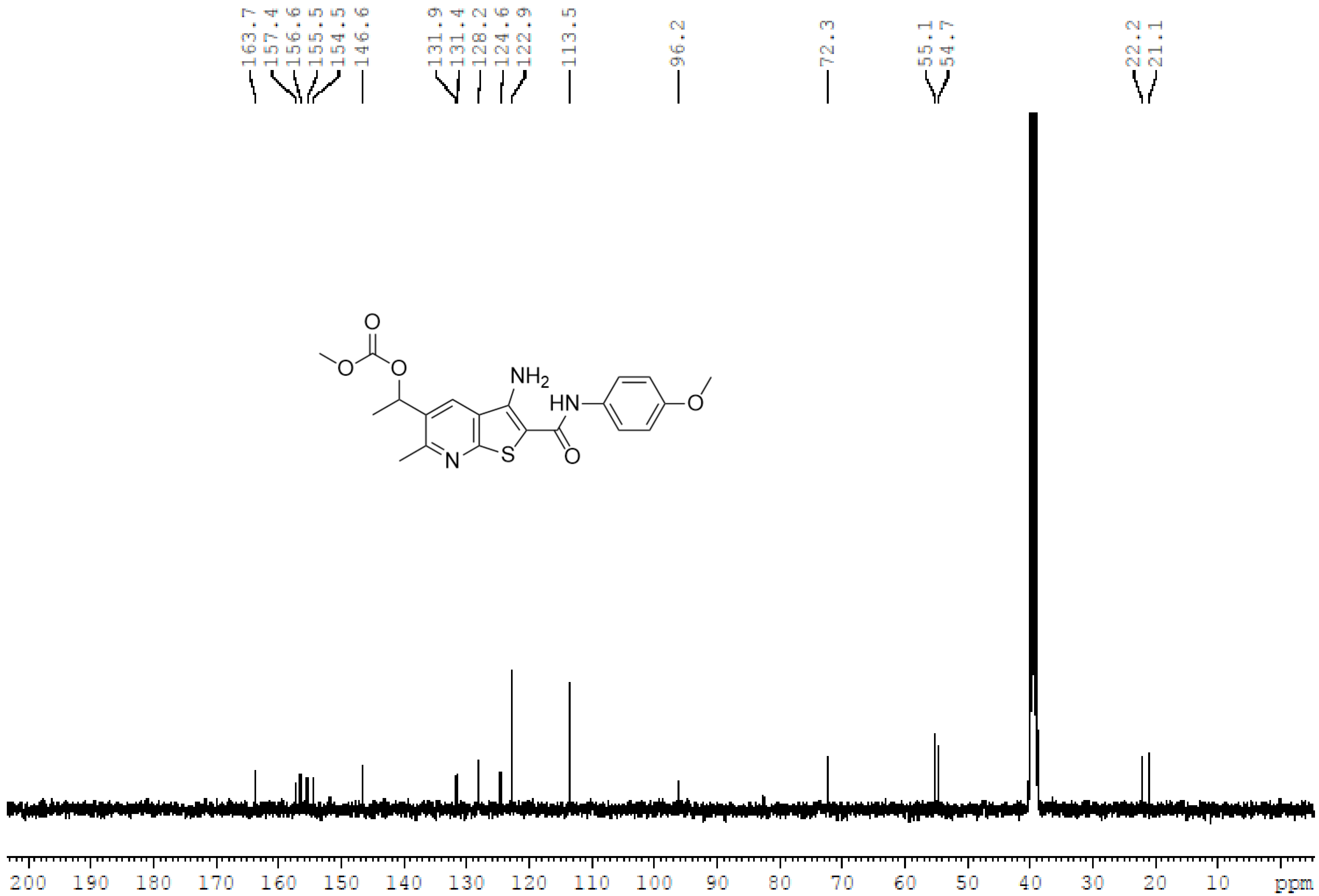

Figure S30: ^{13}C NMR spectrum of **8e** (100 MHz; DMSO-d_6).

Acquisition Parameter

Source Type	ESI	Ion Polarity	Positive	Set Nebulizer	0.4 Bar
Focus	Active	Set Capillary	4500 V	Set Dry Heater	180 °C
Scan Begin	50 m/z	Set End Plate Offset	-500 V	Set Dry Gas	4.0 l/min
Scan End	1000 m/z	Set Collision Cell RF	150.0 Vpp	Set Divert Valve	Waste

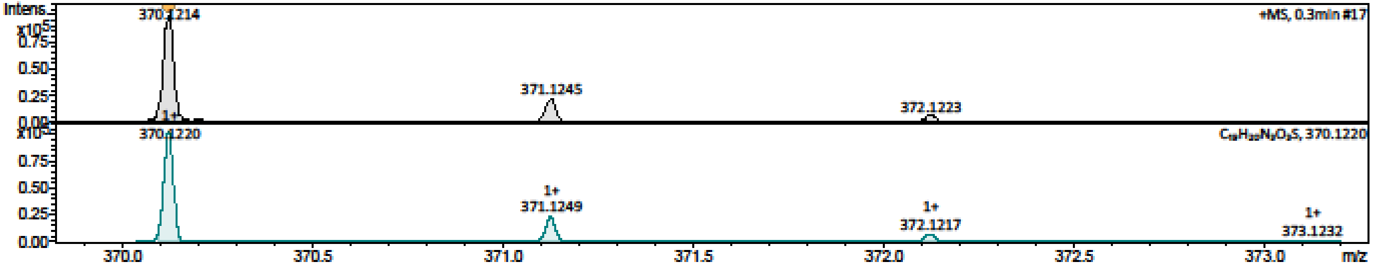

Meas. m/z	#	Ion Formula	m/z	err [ppm]	mSigma	# Sigma	Score	rdb	e⁻ Conf	N-Rule
370.1214	1	C19H20N3O3S	370.1220	-1.7	9.1	1	100.00	11.5	even	ok
	1	C19H23NaO4S	370.1209	1.2	7.8	1	100.00	8.0	odd	ok

Acquisition Parameter

Source Type	ESI	Ion Polarity	Positive	Set Nebulizer	0.4 Bar
Focus	Active	Set Capillary	4500 V	Set Dry Heater	180 °C
Scan Begin	50 m/z	Set End Plate Offset	-500 V	Set Dry Gas	4.0 l/min
Scan End	1000 m/z	Set Collision Cell RF	150.0 Vpp	Set Divert Valve	Waste

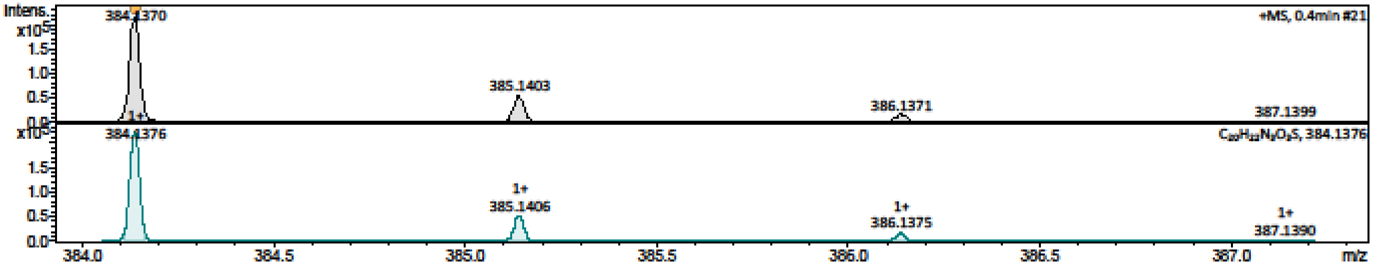

Meas. m/z	#	Ion Formula	m/z	err [ppm]	mSigma	# Sigma	Score	rdb	e⁻ Conf	N-Rule
384.1370	1	C20H22N3O3S	384.1376	1.8	6.1	1	100.00	11.5	even	ok
	1	C20H25NaO4S	384.1366	1.0	11.2	1	100.00	8.0	odd	ok

Figure S31: HRMS of **6a** (top) and **6b** (bottom).

Acquisition Parameter

Source Type	ESI	Ion Polarity	Positive	Set Nebulizer	0.4 Bar
Focus	Active	Set Capillary	4500 V	Set Dry Heater	180 °C
Scan Begin	50 m/z	Set End Plate Offset	-500 V	Set Dry Gas	4.0 l/min
Scan End	1000 m/z	Set Collision Cell RF	150.0 Vpp	Set Divert Valve	Waste

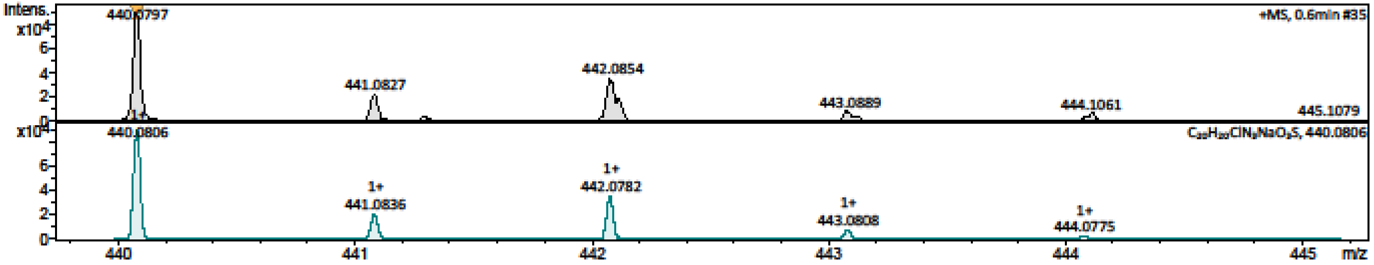

Meas. m/z	#	Ion Formula	m/z	err [ppm]	mSigma	# Sigma	Score	rdb	e⁻ Conf	N-Rule
440.0797	1	C20H20ClN3NaO3S	440.0806	-2.1	13.6	1	100.00	11.5	even	ok

Acquisition Parameter

Source Type	ESI	Ion Polarity	Positive	Set Nebulizer	0.4 Bar
Focus	Active	Set Capillary	4500 V	Set Dry Heater	180 °C
Scan Begin	50 m/z	Set End Plate Offset	-500 V	Set Dry Gas	4.0 l/min
Scan End	1000 m/z	Set Collision Cell RF	150.0 Vpp	Set Divert Valve	Waste

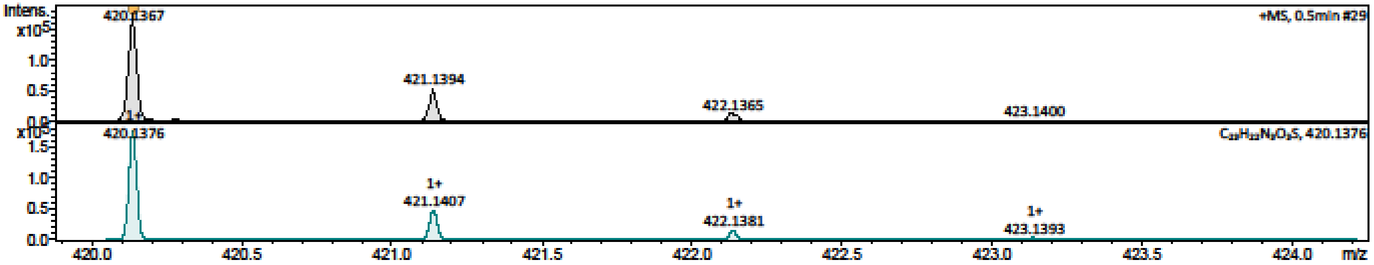

Meas. m/z	#	Ion Formula	m/z	err [ppm]	mSigma	# Sigma	Score	rdb	e⁻ Conf	N-Rule
420.1367	1	C23H22N3O3S	420.1376	-2.2	16.3	1	100.00	14.5	even	ok
	1	C23H25NaO4S	420.1366	-0.3	21.3	1	100.00	11.0	odd	ok

Figure S32: HRMS of **6c** (top) and **6d** (bottom).

Acquisition Parameter

Source Type	ESI	Ion Polarity	Positive	Set Nebulizer	0.4 Bar
Focus	Active	Set Capillary	4500 V	Set Dry Heater	180 °C
Scan Begin	50 m/z	Set End Plate Offset	-500 V	Set Dry Gas	4.0 l/min
Scan End	1000 m/z	Set Collision Cell RF	150.0 Vpp	Set Divert Valve	Waste

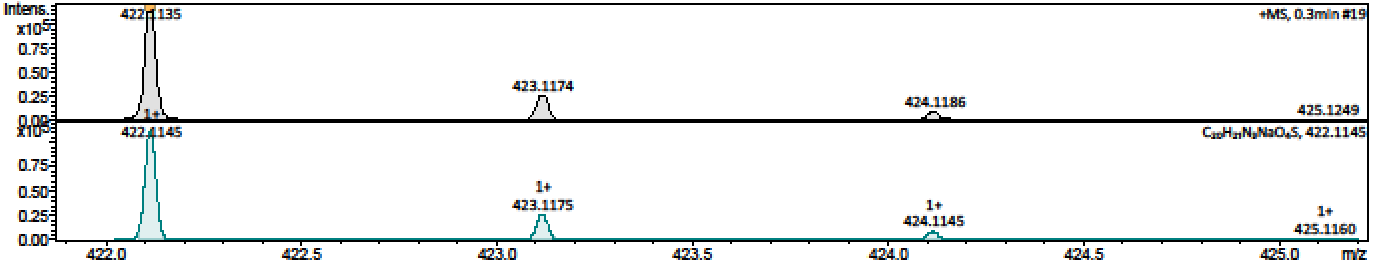

Meas. m/z	#	Ion Formula	m/z	err [ppm]	mSigma	# Sigma	Score	rdb	e⁻ Conf	N-Rule
422.1135	1	C20H21N3NaO4S	422.1145	2.2	3.5	1	100.00	11.5	even	ok

Acquisition Parameter

Source Type	ESI	Ion Polarity	Positive	Set Nebulizer	0.4 Bar
Focus	Active	Set Capillary	4500 V	Set Dry Heater	180 °C
Scan Begin	50 m/z	Set End Plate Offset	-500 V	Set Dry Gas	4.0 l/min
Scan End	1000 m/z	Set Collision Cell RF	150.0 Vpp	Set Divert Valve	Waste

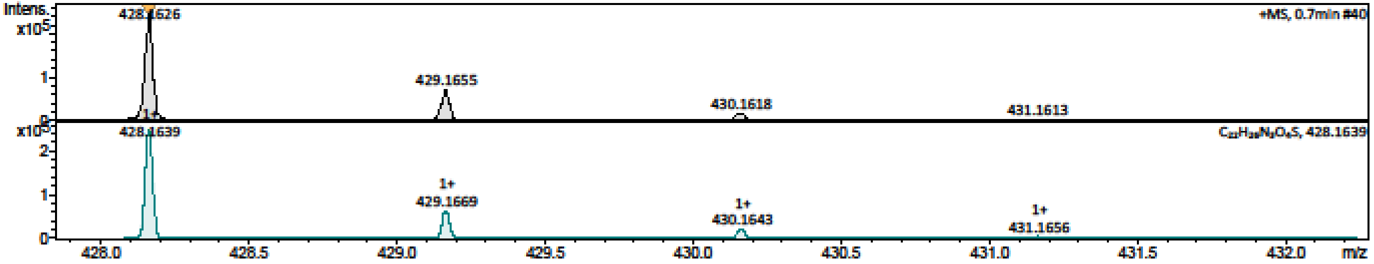

Meas. m/z	#	Ion Formula	m/z	err [ppm]	mSigma	# Sigma	Score	rdb	e⁻ Conf	N-Rule
428.1626	1	C22H26N3O4S	428.1639	3.0	11.3	1	100.00	11.5	even	ok
	1	C22H29NaO5S	428.1628	0.5	16.0	1	100.00	8.0	odd	ok

Figure S33: HRMS of **6e** (top) and **7a** (bottom).

Acquisition Parameter

Source Type	ESI	Ion Polarity	Positive	Set Nebulizer	0.4 Bar
Focus	Active	Set Capillary	4500 V	Set Dry Heater	180 °C
Scan Begin	50 m/z	Set End Plate Offset	-500 V	Set Dry Gas	4.0 l/min
Scan End	1000 m/z	Set Collision Cell RF	150.0 Vpp	Set Divert Valve	Waste

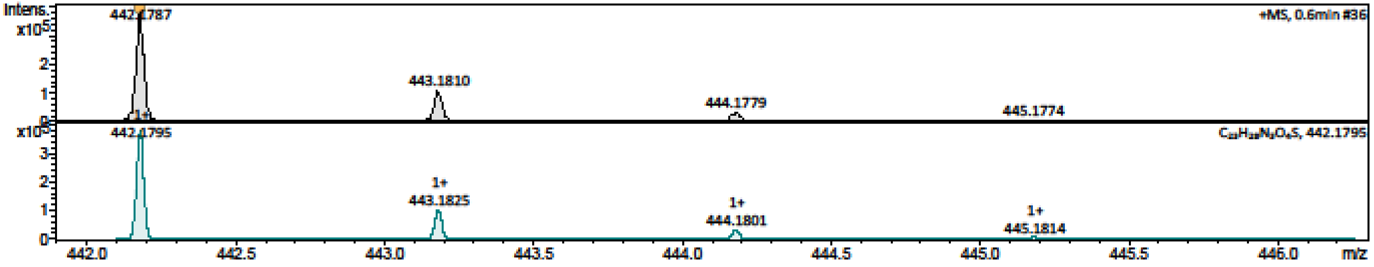

Meas. m/z	#	Ion Formula	m/z	err [ppm]	mSigma	# Sigma	Score	rdb	e⁻ Conf	N-Rule
442.1787	1	C23H28N3O4S	442.1795	-1.7	8.2	1	100.00	11.5	even	ok
	1	C23H31NaO5S	442.1784	-0.7	12.9	1	100.00	8.0	odd	ok

Acquisition Parameter

Source Type	ESI	Ion Polarity	Positive	Set Nebulizer	0.4 Bar
Focus	Active	Set Capillary	4500 V	Set Dry Heater	180 °C
Scan Begin	50 m/z	Set End Plate Offset	-500 V	Set Dry Gas	4.0 l/min
Scan End	1000 m/z	Set Collision Cell RF	150.0 Vpp	Set Divert Valve	Waste

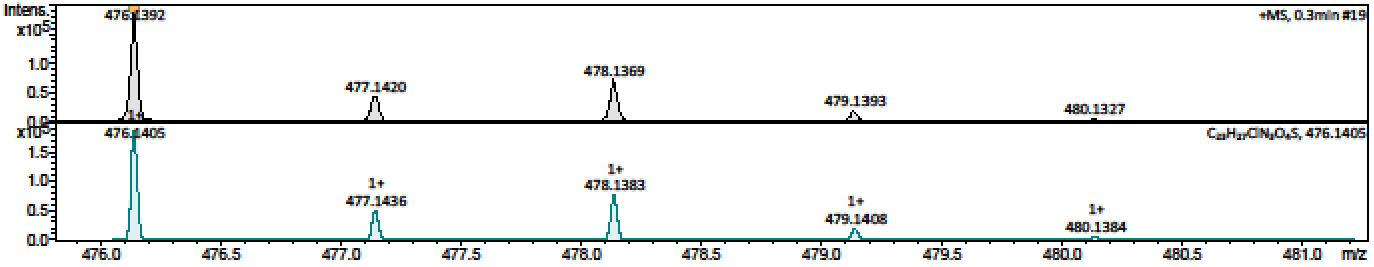

Meas. m/z	#	Ion Formula	m/z	err [ppm]	mSigma	# Sigma	Score	rdb	e⁻ Conf	N-Rule
476.1392	1	C23H27ClN3O4S	476.1405	-2.8	16.2	1	100.00	11.5	even	ok
	1	C23H30ClNaO5S	476.1395	0.6	12.6	1	100.00	8.0	odd	ok

Figure S34: HRMS of **7b** (top) and **7c** (bottom).

Acquisition Parameter

Source Type	ESI	Ion Polarity	Positive	Set Nebulizer	0.4 Bar
Focus	Active	Set Capillary	4500 V	Set Dry Heater	180 °C
Scan Begin	50 m/z	Set End Plate Offset	-500 V	Set Dry Gas	4.0 l/min
Scan End	1000 m/z	Set Collision Cell RF	150.0 Vpp	Set Divert Valve	Waste

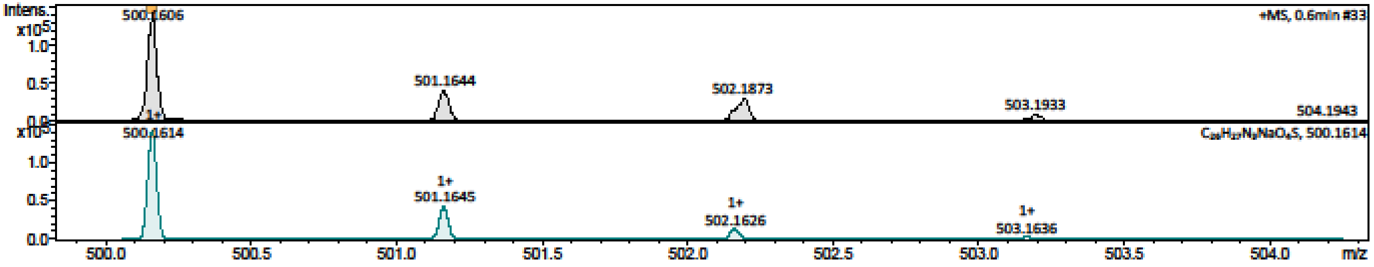

Meas. m/z	#	Ion Formula	m/z	err [ppm]	mSigma	# Sigma	Score	rdb	e⁻ Conf	N-Rule
500.1606	1	C26H24N6O3S	500.1625	3.9	51.4	1	100.00	18.0	odd	ok
	1	C26H27N3NaO4S	500.1614	1.7	65.4	1	100.00	14.5	even	ok

Acquisition Parameter

Source Type	ESI	Ion Polarity	Positive	Set Nebulizer	0.4 Bar
Focus	Active	Set Capillary	4500 V	Set Dry Heater	180 °C
Scan Begin	50 m/z	Set End Plate Offset	-500 V	Set Dry Gas	4.0 l/min
Scan End	1000 m/z	Set Collision Cell RF	150.0 Vpp	Set Divert Valve	Waste

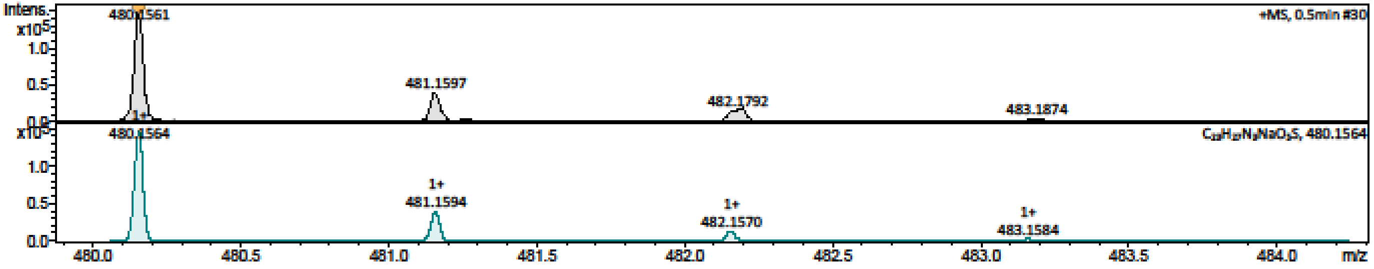

Meas. m/z	#	Ion Formula	m/z	err [ppm]	mSigma	# Sigma	Score	rdb	e⁻ Conf	N-Rule
480.1561	1	C23H24N6O4S	480.1574	2.7	17.3	1	100.00	15.0	odd	ok
	1	C23H27N3NaO5S	480.1564	-0.5	15.8	1	100.00	11.5	even	ok

Figure S35: HRMS of **7d** (top) and **7e** (bottom).

Acquisition Parameter

Source Type	ESI	Ion Polarity	Positive	Set Nebulizer	0.4 Bar
Focus	Active	Set Capillary	4500 V	Set Dry Heater	180 °C
Scan Begin	50 m/z	Set End Plate Offset	-500 V	Set Dry Gas	4.0 l/min
Scan End	1000 m/z	Set Collision Cell RF	150.0 Vpp	Set Divert Valve	Waste

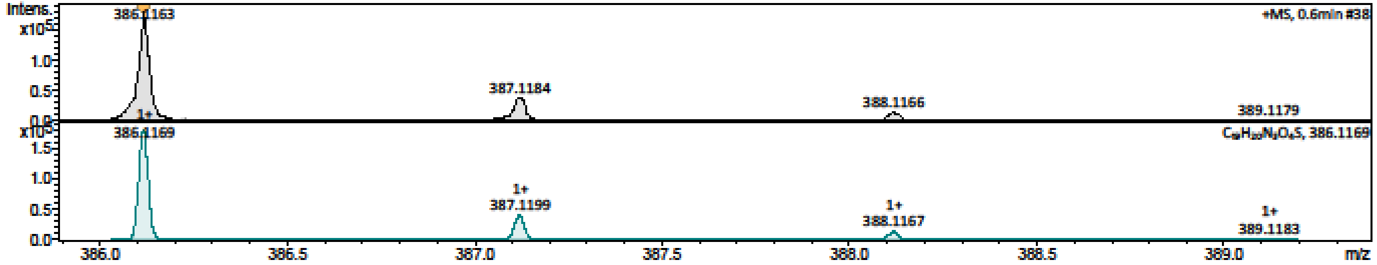

Meas. m/z	#	Ion Formula	m/z	err [ppm]	mSigma	# Sigma	Score	rdb	e⁻ Conf	N-Rule
386.1163	1	C19H20N3O4S	386.1169	-1.6	7.4	1	100.00	11.5	even	ok
	1	C19H23NaO5S	386.1158	1.2	3.9	1	100.00	8.0	odd	ok

Acquisition Parameter

Source Type	ESI	Ion Polarity	Positive	Set Nebulizer	0.4 Bar
Focus	Active	Set Capillary	4500 V	Set Dry Heater	180 °C
Scan Begin	50 m/z	Set End Plate Offset	-500 V	Set Dry Gas	4.0 l/min
Scan End	1000 m/z	Set Collision Cell RF	150.0 Vpp	Set Divert Valve	Waste

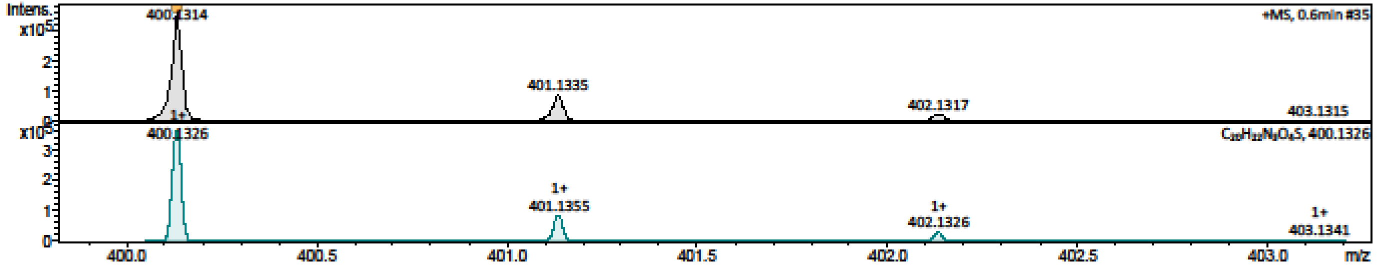

Meas. m/z	#	Ion Formula	m/z	err [ppm]	mSigma	# Sigma	Score	rdb	e⁻ Conf	N-Rule
400.1314	1	C20H22N3O4S	400.1326	-2.8	9.4	1	100.00	11.5	even	ok
	1	C20H25NaO5S	400.1315	-0.2	14.1	1	100.00	8.0	odd	ok

Figure S36: HRMS of **8a** (top) and **8b** (bottom).

Acquisition Parameter

Source Type	ESI	Ion Polarity	Positive	Set Nebulizer	0.4 Bar
Focus	Active	Set Capillary	4500 V	Set Dry Heater	180 °C
Scan Begin	50 m/z	Set End Plate Offset	-500 V	Set Dry Gas	4.0 l/min
Scan End	1000 m/z	Set Collision Cell RF	150.0 Vpp	Set Divert Valve	Waste

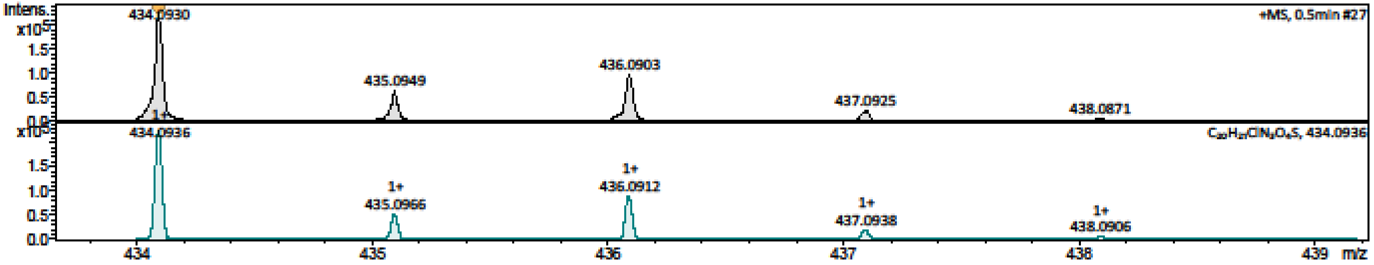

Meas. m/z	#	Ion Formula	m/z	err [ppm]	mSigma	# Sigma	Score	rdb	e⁻ Conf	N-Rule
434.0930	1	C20H21ClN3O4S	434.0936	-1.4	25.5	1	100.00	11.5	even	ok
	1	C20H24ClNaO5S	434.0925	1.1	28.8	1	100.00	8.0	odd	ok

Acquisition Parameter

Source Type	ESI	Ion Polarity	Positive	Set Nebulizer	0.4 Bar
Focus	Active	Set Capillary	4500 V	Set Dry Heater	180 °C
Scan Begin	50 m/z	Set End Plate Offset	-500 V	Set Dry Gas	4.0 l/min
Scan End	1000 m/z	Set Collision Cell RF	150.0 Vpp	Set Divert Valve	Waste

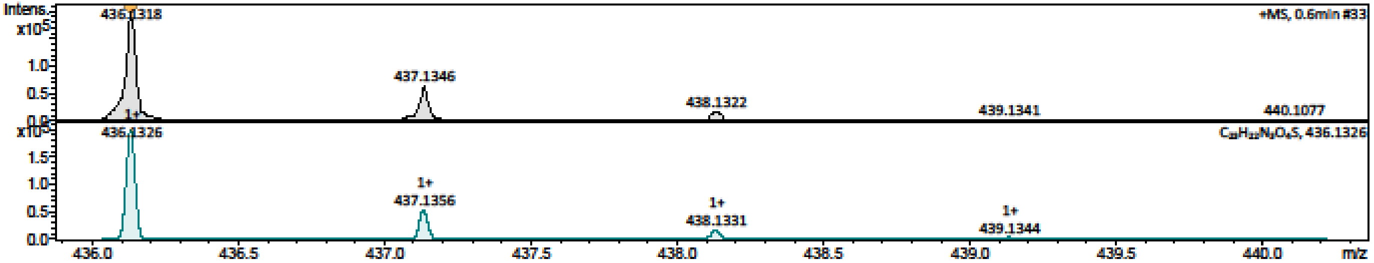

Meas. m/z	#	Ion Formula	m/z	err [ppm]	mSigma	# Sigma	Score	rdb	e⁻ Conf	N-Rule
436.1318	1	C23H22N3O4S	436.1326	-1.8	29.1	1	100.00	14.5	even	ok
	1	C23H25NaO5S	436.1315	0.6	34.3	1	100.00	11.0	odd	ok

Figure S37: HRMS of **8c** (top) and **8d** (bottom).

Acquisition Parameter

Source Type	ESI	Ion Polarity	Positive	Set Nebulizer	0.4 Bar
Focus	Active	Set Capillary	4500 V	Set Dry Heater	180 °C
Scan Begin	50 m/z	Set End Plate Offset	-500 V	Set Dry Gas	4.0 l/min
Scan End	1000 m/z	Set Collision Cell RF	150.0 Vpp	Set Divert Valve	Waste

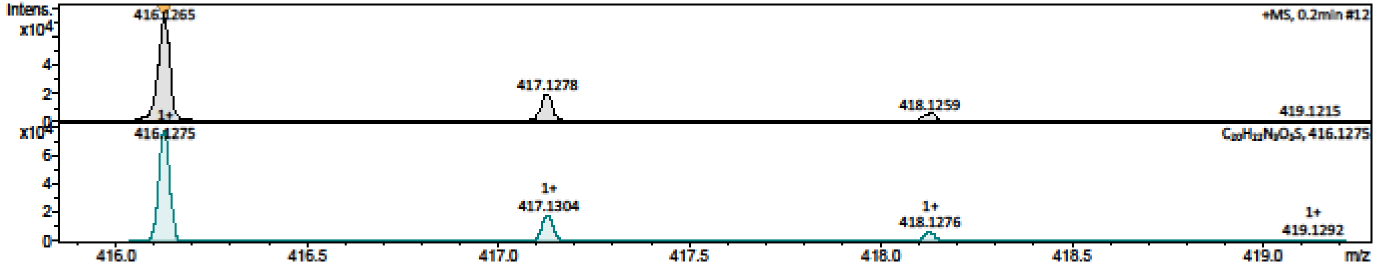

Meas. m/z	#	Ion Formula	m/z	err [ppm]	mSigma	# Sigma	Score	rdb	e⁻ Conf	N-Rule
416.1265	1	C20H22N3O5S	416.1275	-2.4	10.2	1	100.00	11.5	even	ok
	1	C20H25NaO6S	416.1264	-0.2	15.3	1	100.00	8.0	odd	ok

Figure S38: HRMS of **8e**.

Table S1: Computational logP and logS values for thieno[2,3-*b*]pyridines **5a-e**, **6a-e**, **7a-e**, and **8a-e** as calculated by ChemDraw Professional 19.1.1.21. * denotes previously tested parent alcohol-containing thienopyridines.[1]

	LogP	LogS
**5a***	2.93	-4.02
**5b***	3.41	-4.22
**5c***	3.97	-4.92
**5d***	3.92	-5.92
**5e***	2.80	-4.07
**6a**	3.16	-4.79
**6b**	3.64	-4.99
**6c**	4.20	-5.68
**6d**	4.15	-6.19
**6e**	3.03	-4.84
**7a**	4.62	-6.05
**7b**	5.10	-6.25
**7c**	5.66	-6.95
**7d**	5.61	-7.46
**7e**	4.49	-6.10
**8a**	3.74	-5.00
**8b**	4.23	-5.20
**8c**	4.79	-5.90
**8d**	4.74	-6.41
**8e**	3.62	-5.05

Table S2: Melting points of compounds **5a-e**, **6a-e**, **7a-e**, and **8a-e**. * denotes previously tested parent alcohol-containing thienopyridines.[1]

	Melting point (°C)
**5a***	> 230
**5b***	143-145
**5c***	> 230
**5d***	216-218
**5e***	> 230
**6a**	203-205
**6b**	196-198
**6c**	209-211
**6d**	190-192
**6e**	> 230
**7a**	190-192
**7b**	187-189
**7c**	199-201
**7d**	202-204
**7e**	196-198
**8a**	194-196
**8b**	213-215
**8c**	202-204
**8d**	188-190
**8e**	> 230

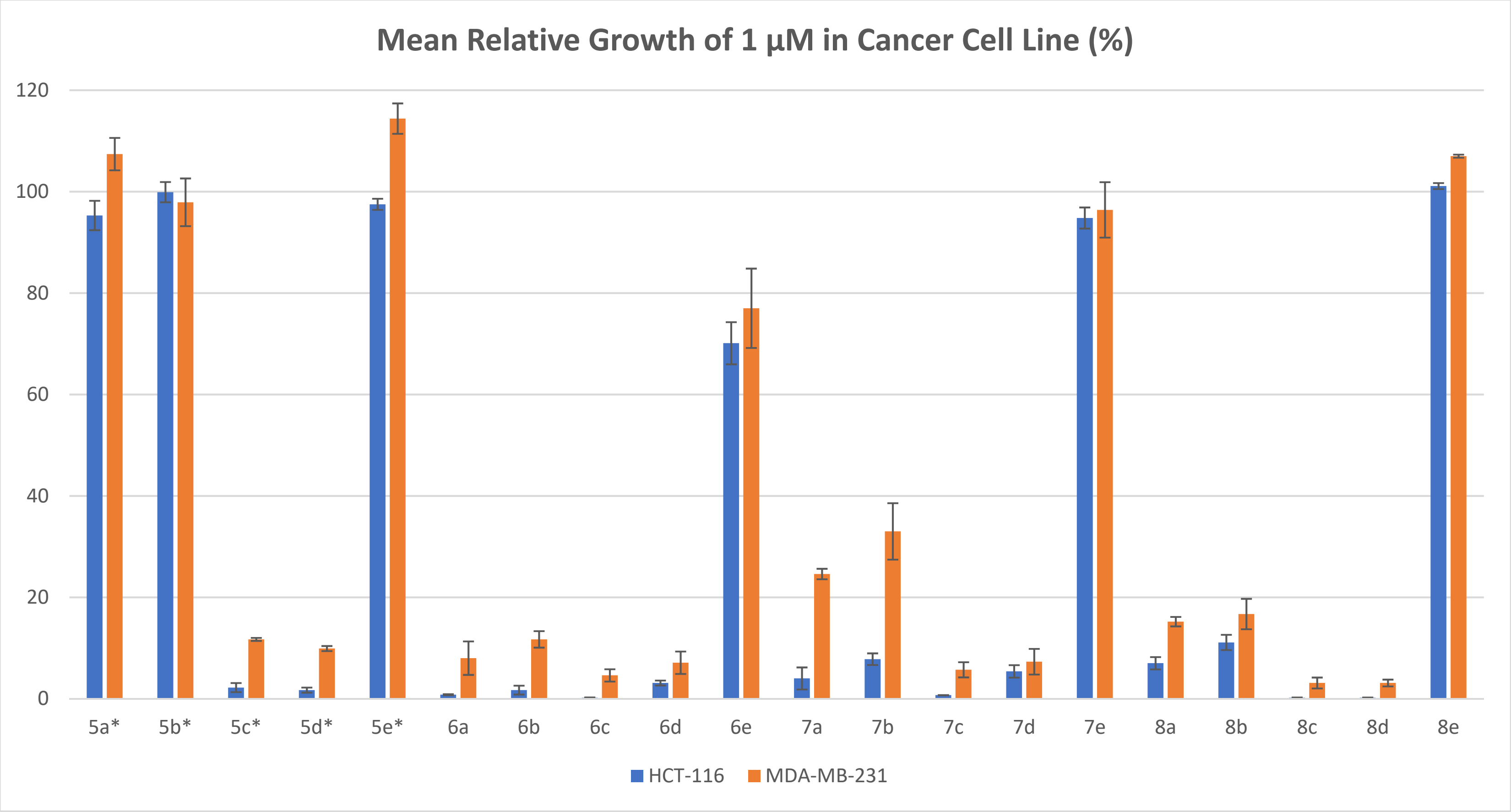

Figure S39: Antiproliferative results of compounds **5a-e**, **6a-e**, **7a-e**, and **8a-e**. * denotes previously tested parent alcohol-containing thienopyridines.[1]
